# The role of seven tumor-associated autoantibodies in the diagnosis, staging and treatment guidance of lung cancer

**DOI:** 10.1186/s12890-024-03060-3

**Published:** 2024-05-21

**Authors:** Heng Ma, Tingting Wu, Qipan Zhang, Qunli Ding

**Affiliations:** Department of Respiratory and Critical Care Medicine, Key Laboratory of Respiratory Disease of Ningbo, The First Affiliated Hospital of Ningbo University, Ningbo, China

**Keywords:** Autoantibody, Diagnosis, Staging, Lung cancer, Treatment guidance

## Abstract

**Background:**

This study assessed the diagnosis, staging and treatment guidance of lung cancer (LC) based on seven tumor-associated autoantibodies (TAAbs) —p53, PGP9.5, SOX2, GBU4-5, MAGE A1, CAGE, and GAGE7.

**Methods:**

ELISA was used to determine the TAAb serum levels in 433 patients diagnosed with LC (161 surgical patients) and 76 patients with benign lung disease (16 surgical patients). The statistical characteristic of the TAAbs was compared among patients with different clinicopathological features. Pre- to postoperative changes in TAAb levels were analyzed to determine their value of LC.

**Results:**

Among all patients, the positive rate of the seven TAAbs was 23.4%, sensitivity was 26.3%, accuracy was 36.3%, specificity was 93.4%, positive predictive value was 95.8%, and negative predictive value was 18.2%; the positive rate for the LC group (26.3%) was significantly higher than that for the benign group (6.6%; *P* < 0.001). Significant differences in the positive rate of the seven autoantibodies according to age (*P* < 0.001), smoking history (*P* = 0.009) and clinical LC stage (*P* < 0.001) were found. Smoking was positively associated with the positive of TAAbs (Τ = 0.118, *P* = 0.008). The positive rates of the seven TAAbs for squamous carcinoma (54.5%), other pathological types (44.4%) and poorly differentiated LC (57.1%) were significantly higher than those for the other types. The positive rate of GBU4-5 was highest among all TAAbs, and the SOX2 level in stage III-IV patients was much higher than that in other stages. For patients undergoing surgery, compared with the preoperative levels, the postoperative levels of the 7 markers, particularly p53 (*P* = 0.027), PGP9.5 (*P* = 0.007), GAGE7 (*P* = 0.014), and GBU4-5 (*P* = 0.002), were significantly different in the malignant group, especially in stage I-II patients, while no clear pre- to postoperative difference was observed in the benign group.

**Conclusions:**

When the seven TAAbs was positive, it was very helpful for the diagnosis of LC. The 7 TAAbs was valuable for staging and guiding treatment of LC in surgical patients.

**Supplementary Information:**

The online version contains supplementary material available at 10.1186/s12890-024-03060-3.

## Background

Lung cancer (LC) is the most frequently occurring cancer and the leading cause of cancer death in men and the third most commonly diagnosed cancer and the leading cause of cancer death in women [1]. LC is classified broadly into non-small cell LC (NSCLC) (85% of total diagnoses) or small cell LC (SCLC) (15% of total diagnoses); among NSCLC classifications, adenocarcinomas are the most common subtype, followed by squamous-cell carcinomas [2]. The current mortality rate of LC remains relatively high, and the 5-year survival rate is unsatisfactory [3].

Early detection and treatment of lung cancer are a promising task to decrease lung mortality [4–6]. In contrast to computed tomography (CT)-guided lung biopsy (an invasive operation with numerous risks), low-dose CT scans (excessive false-positive results making subsequent medical procedures costlier, and repeated CT scanning raises the concern of an increased risk of developing radiation-related cancer) and molecular biology techniques (such as gene sequencing, which possess a low application rate due to their high costs) [4, 7], the assessment of blood tumor biomarkers has the potential for the early diagnosis of LC, as it has advantages including noninvasiveness and convenience of accessibility [8, 9].

Tumor-associated autoantibodies (TAAbs) are produced in the early stage of cancers by the humoral immune response, triggered by abnormal expression of tumor-associated antigens (TAAs). In comparison with other types of biomarkers, serum TAAbs appear earlier and are more stable [10]. They are promising biomarkers that could be applied for the early diagnosis of cancers [11].

A previous study found that a panel consisting of 4 autoantibodies (NOLC1, HMMR, MALAT1 and SMOX) was associated with early stage lung cancer in Chinese patients, and TAAb panels have shown better diagnostic performance than single TAAbs [12]. Given the heterogeneity of human lung cancers, researchers have tried to include more autoantibodies (AABs) to achieve higher sensitivity with a study that used 7 AABs (p53, c-myc, HER2, NY-ESO-1, CAGE, MUC1, and GBU4-5) in European patients with lung cancer (n D 104), a sensitivity of 76% and specificity of 92% were observed [13]; and an audit study of EarlyCDT(R)-Lung (6-AABs or 7-AABs) in 1600 patients also showed high specificity of 83% or 91% [14]. Since there are noticeable differences in the genetic makeup of European and Asian lung cancer patients, this panel of AABs may not be ideal for the Chinese population, and a similar study needs to be performed in Chinese patients to confirm these results, then 7 antigens (p53, PGP9.5, SOX2, GAGE7, GBU4-5, MAGE A1 and CAGE) were identified from 43 cancer-related antigens in a large clinical multicenter study and almost all of the AABs demonstrated good discriminative ability between lung cancer and healthy controls; researchers also compared the sensitivity values for traditional tumor markers, the 7-TAAbs panel showed a higher sensitivity in the early stages of lung cancer [4].

Specifically, p53 is a tumor suppressor gene involved in regulating the cell cycle [15, 16]. PGP9.5 is a ubiquitinase expressed in neural tissue and various malignant tumors, including LC cells [17, 18]. MAGE A1 belongs to the human melanoma antigen family and is a special tumor antigen that is thought to be involved in the occurrence of various tumors [19]. SOX2 is a transcription factor belonging to the SOX family that is involved in the proliferation and development of various cancers, demonstrating increased abundance [20]. CAGE is a cancer-associated gene that is expressed in a variety of cancers but not in normal tissues except the testis [21]. GBU4-5 is another protein described as inducing autoantibodies in LC [22]. Finally, GAGE7 is one of ten members of the GAGE family that have been identified; GAGE2-8 differ from each other mainly by single nucleotide substitutions resulting in amino acid substitutions. GAGE proteins share no homology with any protein of known function, and their functions remain unknown [23].

However, as clinical biomarkers, single functionality is clearly not enough, and more functions need to be explored. Hence, the aim of this study was to verify the diagnostic value of these seven TAAbs and explore their other value of LC.

## Methods

### Sample information

We collected data for 480 patients with pulmonary nodules and 29 patients with nonneoplastic disease from The First Affiliated Hospital of Ningbo University from July 2022 to December 2022; 433 patients were diagnosed with LC (161 surgical patients), and 76 patients had benign lung disease (16 surgical patients). Among all patients, 227 males and 282 females were included, ranging in age from 23 to 91 years, with a median age of 60 years. A total of 433 patients were in the LC group, namely, 192 males and 241 females, aged 23–91 years old, with a median age of 60 years; according to histopathological staging, 344 patients had adenocarcinoma, 44 had squamous cell carcinoma, 11 had small-cell carcinoma, 9 had other types of LC, and 7 had poorly differentiated carcinoma. In terms of TNM staging, 277 patients had stage I cancer, 13 had stage II, 53 had stage III, and 29 had stage IV cancer. There were 76 patients with benign lung disease, namely, 35 males and 41 females, aged 23–89, with a median age of 58.5 years.

This study was reviewed and approved by the ethics committee of The First Affiliated Hospital of Ningbo University. Informed consent was obtained from all participants. The ethical approval number: 2024-089RS.

### Inclusion and exclusion criteria

The inclusion criteria were as follows: (1) pulmonary nodules diagnosed as LC or benign lung disease by pathology examination and (2) other benign diseases with no evidence of malignancy. The exclusion criteria were as follows: (1) unclear LC staging, (2) evidence of active malignancy other than LC within six months and (3) autoimmune disease.

### Serum sample collection and processing

Serum from 5 mL of fasting blood was separated by centrifugation at 3500 r/min (2410 g) for 5 min, completed within 8 h if the specimen could not be detected in time, and stored at 2–8 °C.

### Reagents and equipment

An ELISA was used in the test according to the 7-TAAbs assay kit (Hangzhou Cancer probe Biotech Company). The OD value of each sample was measured with a microplate reader (ST360, Shanghai Kehua Biotechnology Co., Ltd.).

### Enzyme-linked immunosorbent assay (ELISA)

The ELISA kit was used according to the manufacturer’s instructions. The positive reference values of the seven TAAbs were as follows: p53 ≥ 13.1 U/ml, PGP9.5 ≥ 11.1 U/ml, SOX2 ≥ 10.3 U/ml, GAGE7 ≥ 14.4 U/ml, GBU4-5 ≥ 7.0 U/ml, MAGE A1 ≥ 11.9 U/ml, and CAGE ≥ 7.2 U/ml. If one of the seven autoantibodies was positive, the patient was said to be positive for the 7 TAAbs; if all seven autoantibodies were negative, the patient was considered negative. The optimal cutoff values for the 7 AABs were defined as an optical density (OD) value greater than either the mean plus 2 standard deviations (SDs) or the mean plus 3 SDs of the normal cohort in the training set and the cutoff values were optimized using a Monte Carlo direct search method to find a set of antigen-specific cutoffs yielding the maximum sensitivity for a fixed specificity of 90%; The more stringent cut-off point (3 SDs) was applied to the PGP9.5, SOX2, p53, GAGE7 and CAGE autoantibody assays, incorporating, on average, 99% of the distribution of the data. An OD value greater than the mean plus 2 SDs of the normal population was applied to the GBU4-5 and MAGEA1 autoantibody assays [4].

### Statistical analysis

SPSS version 26.0 software was used for data analysis. Chi-square analysis was used to compare the positive rate of the 7 TAAbs between groups. Kendall correlation analysis was used to find the correlation between smoking and the seven TAAbs. The paired sample T test was used to compare the changes in the levels of TAAbs preoperatively and postoperatively. A *P* value < 0.05 was considered to indicate statistical significance. GraphPad Prism 10.0.2 software was used for image processing.

## Results

### Positive rate of the seven autoantibodies for different characteristics and different groups

In terms of age, there was an obvious difference in the positive rate (χ2 = 19.463, *P* < 0.001) of the seven autoantibodies: 31.3% in the older group (≥ 60 years) and 14.8% in the younger group (< 60 years). There was no significant difference in the positive rate (χ2 = 0.381, *P* = 0.599) of the seven TAAbs between the sexes (24.7% male, 22.3% female). The positive rate of the 7 TAAbs among smokers (32.7%) was significantly higher than that in nonsmokers (20.7%) (χ2 = 7.11, *P* = 0.009), and smoking was positively associated with the positive of TAAbs (Τ = 0.118, *P* = 0.008). Regarding clinical stage, the positive rate of the seven TAAbs for stage III-IV cancer (54.7% in stage III, 51.7% in stage IV) was significantly higher than that for stages I (18.8%) and II (23.1%; χ2 = 39.599, *P* < 0.001). Regarding the different types of LC, the positive rates of the seven TAAbs for squamous carcinoma (54.5%), other pathological types (44.4%) and poorly differentiated LC (57.1%) were significantly higher than those for other types (Table 1).

Among all patients, the positive rate of the seven autoantibodies was 23.4%, sensitivity was 26.3%, accuracy was 36.3%, specificity was 93.4%, positive predictive value was 95.8%, and negative predictive value was 18.2%.

In the LC group, the positive rate of the seven autoantibodies (26.3%) was significantly higher than that in the benign group (6.6%; χ2 = 14.077, *P* < 0.001) (Table 1). Notably, in the benign group, 60% of all positive patients had a history of chronic obstructive pulmonary disease (COPD).

**Table 1:** Comparison of the positive rate of the seven TAAbs among the patients

Features	Negative	Positive	x2	*P*
Age (years)				
≥ 60	182	83(31.3%)	19.463	< 0.001
< 60	208	36(14.8%)		
Sex				
Male	171	56(24.7%)	0.381	0.599
Female	219	63(22.3%)		
Smoking history				
Yes	76	37(32.7%)	7.11	0.009
No	314	82(20.7%)		
Pathological type				
Benign	71	5(6.6%)	14.077	< 0.001
Malignant	319	114(26.3%)		
Precancerous	13	5(27.8%)		
Adenocarcinoma	271	73(21.2%)		
Squamous	20	24(54.5%)		
SCLC	7	4(36.4%)		
Other pathological types	5	4(44.4%)		
Poorly differentiated	3	4(57.1%)		
Clinical stage				
I	225	52(18.8%)	39.599	< 0.001
II	10	3(23.1%)		
III	24	29(54.7%)		
IV	14	15(51.7%)		

### Diagnostic efficacy of the different markers for different types and clinical stages of lung cancer

For precancer, adenocarcinoma and other pathological types, the positive rate of GBU4-5 was highest among all markers (11.1% for precancer, 8.1% for adenocarcinoma, 22.2% for other pathological types). SOX2, GBU4-5 and MAGE A1 accounted for the highest proportion (18.2%) of squamous carcinomas. For SCLC and poorly differentiated cancer, SOX2 was most commonly observed (18.2% and 28.6%, respectively). In stage I-IV cancer, the positive rates of GBU4-5 (8.3%), MAGE A1 (15.4%), GBU4-5 and SOX2 (both 18.9%), and SOX2 (27.6%) were the highest, respectively (Table 2).

**Table 2:** Baseline characteristics of the patients

Features	Total (*n*)	P53,*n*(%)	PGP9.5,*n*(%)	SOX2,*n*(%)	GAGE7,*n*(%)	GBU4-5,*n*(%)	MAGE A1,*n*(%)	CAGE, *n*(%)
Age (years)	509							
≥ 60	265	4(1.5)	11(4.2)	23(8.7)	15(5.7)	35(13.2)	10(3.8)	6(2.3)
<60	244	0(0)	1(0.4)	10(4.1)	9(3.7)	10(4.1)	3(1.2)	6(2.5)
Sex								
Male	227	4(1.8)	8(3.5)	23(10.1)	8(3.5)	17(7.5)	12(5.3)	2(0.9)
Female	282	0(0)	4(1.4)	10(3.5)	16(5.7)	28(9.9)	1(0.4)	10(3.5)
Smoking history								
Yes	113	2(1.8)	3(2.7)	16(14.2)	6(5.3)	11(9.7)	8(7.1)	1(0.9)
No	396	2(0.5)	9(2.3)	17(4.3)	18(4.5)	34(8.6)	5(1.3)	11(2.8)
Pathological type								
Benign	76	0(0)	1(1.3)	2(2.6)	0(0)	3(3.9)	0(0)	0(0)
Malignant	433	4(0.9)	11(2.5)	31(7.2)	24(5.5)	42(9.7)	13(3.0)	12(2.8)
Precancerous	18	0(0)	0(0)	1(5.6)	1(5.6)	2(11.1)	0(0)	1(5.6)
Adenocarcinoma	344	2(0.6)	5(1.5)	17(4.9)	18(5.2)	28(8.1)	4(1.2)	10(2.9)
Squamous	44	1(2.3)	4(9.1)	8(18.2)	4(9.1)	8(18.2)	8(18.2)	0(0)
SCLC	11	1(9.1)	0(0)	2(18.2)	1(9.1)	1(9.1)	0(0)	0(0)
Other pathological types	9	0(0)	1(11.1)	1(11.1)	0(0)	2(22.2)	0(0)	0(0)
Poorly differentiated	7	0(0)	1(14.3)	2(28.6)	0(0)	1(14.3)	1(14.3)	1(14.3)
Clinical stage								
I	277	0(0)	3(1.1)	11(4.0)	15(5.4)	23(8.3)	1(0.4)	6(2.2)
II	13	0(0)	1(7.7)	0(0)	1(7.7)	0(0)	2(15.4)	0(0)
III	53	3(5.7)	4(7.5)	10(18.9)	4(7.5)	10(18.9)	8(15.1)	1(1.9)
IV	29	0(0)	1(3.4)	8(27.6)	2(6.9)	1(3.4)	2(6.9)	2(6.9)

### Role of the levels of the seven TAAbs in surgical patients

**Fig. 1:**
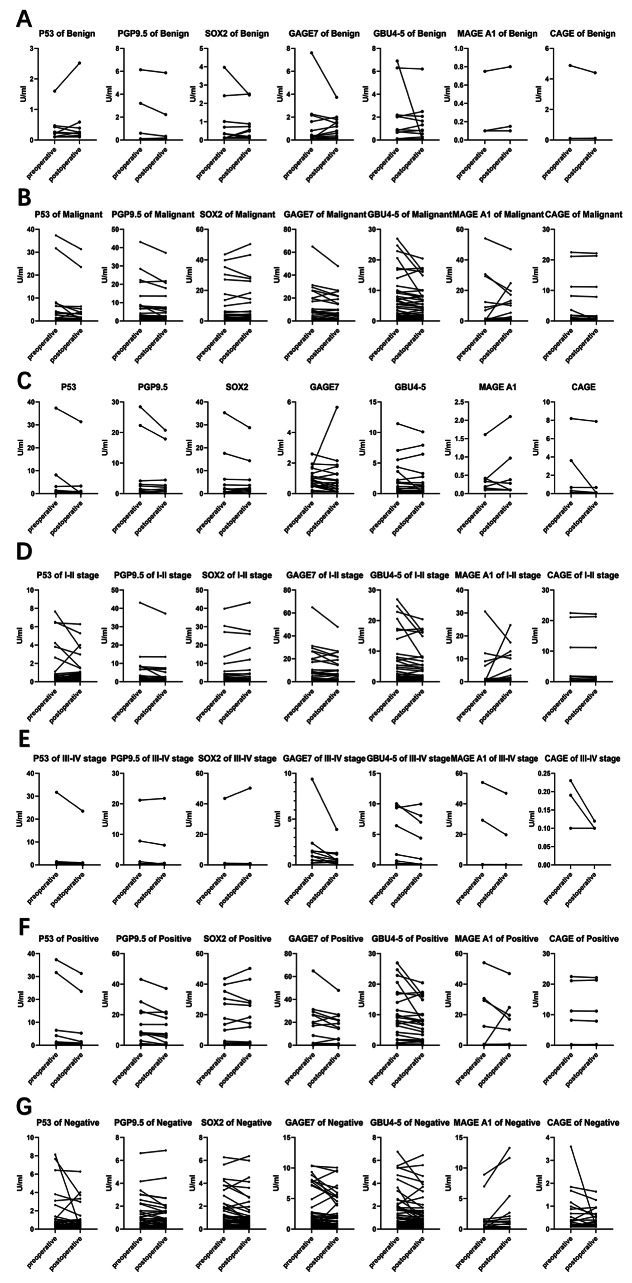
Comparison of the preoperative and postoperative levels of each marker **A**: Pre- and postoperative levels of each marker in benign patients. **B**: Pre- and postoperative levels of each marker in malignant patients. **C**: Pre- and postoperative levels of each marker in patients with precancerous and preinvasive lesions. **D**: Pre- and postoperative levels of each marker in stage I-II lung cancer (LC) patients. **E**: Pre- and postoperative levels of each marker in stage III-IV LC patients. **F**: Pre- and postoperative levels of each marker in LC patients positive for the 7 TAAbs. **G**: Pre- and postoperative levels of each marker in LC patients negative for the 7 TAAbs

When comparing preoperative and postoperative values in patients undergoing surgery, there was no clear difference in any of the seven markers in the benign group (*P* > 0.05). In contrast, a significant difference in the levels of the markers was found in the malignant group, including p53 (*P* = 0.027), PGP9.5 (*P* = 0.007), GAGE7 (*P* = 0.014), and GBU4-5 (*P* = 0.002). In a subgroup analysis of the malignant group, we divided the patients by clinical stage and whether they were positive for the 7 TAAbs. For precancerous and preinvasive lesions, there was no clear difference in any of the seven markers (*P* > 0.05). For stage I-II cancer, obvious differences in 3 markers, namely, PGP9.5 (*P* = 0.034), GAGE7 (*P* = 0.03), and GBU4-5 (*P* = 0.011), were found. For stage III-IV cancer, a significant difference in GBU4-5 (*P* = 0.049) was found. Among patients positive for the 7 TAAbs, a clear pre- to postoperative difference in PGP9.5 (*P* = 0.014) and GBU4-5 (*P* = 0.019) was found. For the negative group, obvious differences in SOX2 (*P* = 0.007), GAGE7 (*P* = 0.046), GBU4-5 (*P* = 0.003) and MAGE A1 (*P* = 0.04) were found (Table 3) (Fig. 1).

**Table 3:** Comparison of the levels of the seven TAAbs among surgical patients

	P53	PGP9.5	SOX2	GAGE7	GBU4-5	MAGE A1	CAGE
**Benign (*n*** **= 16)**							
preoperative	0.2631 ± 0.37636	0.7025 ± 1.64385	0.6144 ± 1.07812	1.0462 ± 1.89998	1.4556 ± 2.14516	0.1406 ± 0.1625	0.3988 ± 1.195
postoperative	0.3406 ± 0.59620	0.6125 ± 1.49761	0.5562 ± 0.79348	0.8931 ± 1.01793	1.0581 ± 1.57809	0.15 ± 0.17416	0.3694 ± 1.07484
*P*	0.24	0.18	0.592	0.587	0.37	0.083	0.344
**Malignant (** ***n*** ** = 161)**							
preoperative	0.9521 ± 3.97973	1.4417 ± 4.94694	1.9398 ± 6.41946	2.5919 ± 7.24029	2.1261 ± 4.60727	1.0487 ± 5.49842	0.5658 ± 2.6426
postoperative	0.7525 ± 3.14608	1.2114 ± 4.22252	1.8993 ± 6.60443	2.1806 ± 5.78999	1.7202 ± 3.67907	1.1168 ± 4.84851	0.5359 ± 2.61552
*P*	0.027	0.007	0.614	0.014	0.002	0.732	0.202
Precancerous and preinvasive (*n* = 25)							
preoperative	2.254 ± 7.4977	2.6532 ± 6.97219	2.966 ± 7.6446	0.7904 ± 0.65736	1.848 ± 2.7198	0.1996 ± 0.30798	0.5972 ± 1.7328
postoperative	1.6204 ± 6.2279	2.1156 ± 5.30436	2.5184 ± 6.2542	0.7972 ± 1.18876	1.6224 ± 2.66223	0.2332 ± 0.42935	0.434 ± 1.55543
*P*	0.119	0.135	0.145	0.969	0.195	0.336	0.255
I-II stage (*n* = 125)							
preoperative	0.4811 ± 1.14884	1.073 ± 4.26052	1.5211 ± 5.22626	3.0238 ± 8.13091	2.0571 ± 4.92381	0.6346 ± 3.08482	0.5988 ± 2.90009
postoperative	0.4357 ± 0.90017	0.897 ± 3.68553	1.5099 ± 5.41842	2.5742 ± 6.49554	1.643 ± 3.84693	0.8494 ± 3.20852	0.5945 ± 2.8873
*P*	0.382	0.034	0.858	0.03	0.011	0.365	0.687
III-IV stage (*n* = 11)							
preoperative	3.3455 ± 9.42432	2.8791 ± 6.49316	4.3645 ± 13.00436	1.7791 ± 2.5987	3.5427 ± 4.33514	7.6836 ± 17.69532	0.12 ± 0.04539
postoperative	2.3809 ± 7.01241	2.7291 ± 6.59575	4.9164 ± 15.06978	0.8527 ± 1.07298	2.82 ± 3.82409	6.1645 ± 14.73634	0.1018 ± 0.00603
*P*	0.216	0.387	0.4	0.086	0.049	0.172	0.169
Positive (*n* = 36)							
preoperative	2.5047 ± 8.00358	4.8953 ± 9.65383	6.4011 ± 12.5587	7.2419 ± 13.87435	6.8028 ± 7.90542	3.6389 ± 11.2089	1.845 ± 5.40247
postoperative	1.9619 ± 6.39576	3.9881 ± 8.28589	6.5133 ± 12.93565	5.9956 ± 10.94976	5.4981 ± 6.22263	3.4411 ± 9.52054	1.8289 ± 5.37483
*P*	0.068	0.014	0.746	0.069	0.019	0.82	0.294
Negative (*n* = 125)							
preoperative	0.505 ± 1.20597	0.4471 ± 0.90096	0.6549 ± 1.10126	1.2527 ± 2.28506	0.7793 ± 1.25356	0.3027 ± 1.01899	0.1974 ± 0.40093
postoperative	0.4042 ± 0.82367	0.4118 ± 0.8628	0.5704 ± 1.04419	1.0819 ± 1.99315	0.6322 ± 1.09498	0.4474 ± 1.65191	0.1635 ± 0.21856
*P*	0.205	0.098	0.007	0.046	0.003	0.04	0.257

Note: Precancerous and preinvasive: precancerous and preinvasive tumors of surgical patients diagnosed with lung cancer (LC). I-II stage: stage I-II tumors of surgical patients diagnosed with LC. III-IV stage: stage III-IV tumors of surgical patients diagnosed with LC. Positive: Surgical patients diagnosed with LC and positive for the seven tumor-associated autoantibodies (TAAbs). Negative: Surgical patients diagnosed with LC and negative for the 7 TAAbs.

## Discussion

LC is the most common tumor worldwide; yet more methods need to be developed to diagnose the disease early, staging and guiding treatment. Although early detection of the 7 TAAbs in hematological tests is necessary, it is insufficient for improving the survival rate of patients. In recent years, studies on the efficacy of TAAbs have shown varied but analogous results with increasingly abundant clinical evidence.

In a systematic review [24], studies of various TAAbs in different countries and ethnic groups were recorded. Twelve articles reported on autoantibodies against p53 and found sensitivities ranging from 12.6 to 40.3% and specificities ranging from 94.9 to 100%. TAAb panels supplied relatively high sensitivities, and some panels even yielded promising specificities (both > 90%) [25, 26]. A study by Boyle et al. reported a sensitivity of 37.0% for the antigens in the panel of six TAAbs they used, including p53, CAGE, GBU4-5, and SOX2 [27].

In a trial of more than 15,000 people [28], researchers found that the 7-TAAb panel demonstrated its potential as a powerful diagnostic tool for LC detection in a real-world cohort, particularly when combined with LDCT, and it showed a greater sensitivity for detecting ground-glass nodules. They highlight the clinical utility of the 7-TAAb panel in facilitating early detection of LC and that it may have significant implications for improving patient outcomes in this population.

In our study, the positive rate of the 7 TAAbs in elderly patients was obviously higher than that in younger patients, so for the early diagnosis of LC, patients older than 60 seem to be more suitable for the application of a 7-TAAb panel. Therefore, it is more suitable for LC screening in elderly individuals.

The positive rate of the seven TAAbs in patients with a smoking history was much higher than that in nonsmokers and smoking was positively associated with the positive of TAAbs, which may be closely related to smoking as a risk factor for LC. Thus, the 7-TAAb panel may be more suitable for LC screening in smokers.

Regarding the early diagnosis of LC by the seven TAAbs, the positive rate in the malignant group was significantly higher than that in the benign group, consistent with the findings of many studies [29–33], which showed that higher positive rates were observed in the later stage of LC. Combined with promising specificity and positive predictive value, the above results indicate that when the seven TAAbs was positive for patients suspected of lung cancer, it may have good prompt effect in the diagnosis.

In a systematic review and meta-analysis of 11 studies, researchers found that both COPD and emphysema seemed to increase the risk of developing LC [34]. In the benign group, we found that 60% of all positive patients had a COPD diagnosis, consistent with the findings of another study [35]. It seems that such patients are at higher risk of developing LC, but clinical screening with larger samples is needed to evaluate this hypothesis.

Among all 7 TAAbs, the positive rate of GBU4-5 was much higher than that of the other TAAbs in early-stage cancer, suggesting that GBU4-5 is the earliest diagnostic marker to demonstrate substantial changes in level in LC. On the other hand, the positive rate of SOX2 was significantly higher than that of the other markers in patients with stage III-IV disease, similar to previously reported results [20]. SOX2 may thus be a marker of poor prognosis.

Among the different types of LC, especially adenocarcinoma and squamous cell carcinoma, which are the most common types, there are clear differences in the positive rates of the seven autoantibodies. It may indicate that squamous carcinoma cells had faster and stronger humoral immune response to the seven TAAbs than adenocarcinomas, especially in SOX2, GBU4-5 and MAGE A1. The seven autoantibodies have a higher diagnostic value for squamous cell carcinoma. Based on the above data, for patients with high probability of squamous cell carcinoma indicated by clinical history and imaging in the future, the seven TAAbs may be of good benefit to the diagnosis and the change of its value may be of great value for the development of squamous cell carcinoma, the guidance of subsequent treatment and the evaluation of efficacy.

Among surgical patients, our results demonstrated the benefit of comparing the pre- and postoperative levels of LC autoantibodies, regardless of whether the patients were positive or negative for the 7 TAAbs. The possible effect of surgical resection on TAAbs serum concentrations has already excluded when 93 patients’ serum samples collected before and after surgery and the results showed that the serum concentrations of each TAAbs did not change significantly after surgery [4]. This is the most innovative part of this study since, to date, no other study has investigated this topic. The pre- to postoperative change in the levels of the 7 TAAbs may have guiding significance in staging and postoperative treatment of LC. The reason may be that immediately after the complete surgical removal of the lesions in the early stage of LC, a significant decline in the levels of the 7 TAAbs can be detected. Among all markers, changes in the levels of p53, PGP9.5, GAGE7 and GBU4-5 are worthy of attention.

In this research, we used ELISA to detect the pre- and postoperative levels of 7 TAAbs in patients with different features, and we summarized important characteristics on the basis of data differences. The results of the study were used to screen for the specific value of the seven tumor-associated autoantibodies in distinguishing different features of LC and in determining the value of these autoantibodies as biomarkers, enriching the tools available to clinicians for diagnosing, staging and guiding the postoperative treatment of LC.

However, our study only preliminarily suggested the relationship between autoantibodies and lung cancer as well as identified valuable markers. More in-depth studies with larger sample sizes are needed to prove their universality.

## Conclusions

When the seven TAAbs was positive, it was very helpful for the diagnosis of LC. The 7 TAAbs was valuable for staging and guiding treatment of LC in surgical patients.

### Electronic supplementary material

Below is the link to the electronic supplementary material.

Supplementary Material 1

Supplementary Material 2

Supplementary Material 3

Supplementary Material 4

Supplementary Material 5

Supplementary Material 6

Supplementary Material 7

Supplementary Material 8

Supplementary Material 9

Supplementary Material 10

Supplementary Material 11

Supplementary Material 12

Supplementary Material 13

Supplementary Material 14

Supplementary Material 15

Supplementary Material 16

Supplementary Material 17

Supplementary Material 18

Supplementary Material 19

Supplementary Material 20

Supplementary Material 21

Supplementary Material 22

Supplementary Material 23

Supplementary Material 24

Supplementary Material 25

Supplementary Material 26

Supplementary Material 27

Supplementary Material 28

Supplementary Material 29

Supplementary Material 30

Supplementary Material 31

Supplementary Material 32

Supplementary Material 33

Supplementary Material 34

Supplementary Material 35

Supplementary Material 36

Supplementary Material 37

Supplementary Material 38

Supplementary Material 39

Supplementary Material 40

Supplementary Material 41

Supplementary Material 42

Supplementary Material 43

Supplementary Material 44

Supplementary Material 45

Supplementary Material 46

Supplementary Material 47

Supplementary Material 48

Supplementary Material 49

Supplementary Material 50

Supplementary Material 51

## Data Availability

Data is provided within supplementary information files.

## Abbreviations

LC: lung cancer
TAAbs: tumor-associated autoantibodies
NSCLC: non-small cell lung cancer
SCLC: small cell lung cancer
CT: computed tomography
TAAs: tumor-associated antigens
AABs: autoantibodies
ELISA: enzyme-linked immunosorbent assay
OD: optical density
SDs: standard deviations
COPD: chronic obstructive pulmonary disease
