# Supplementary figures and images for: The role of seven tumor-associated autoantibodies in the diagnosis, staging and treatment guidance of lung cancer
Source: BMC Pulm Med. 2024 May 21;24:250. doi: 10.1186/s12890-024-03060-3 (PMC11106964; doi:10.1186/s12890-024-03060-3)

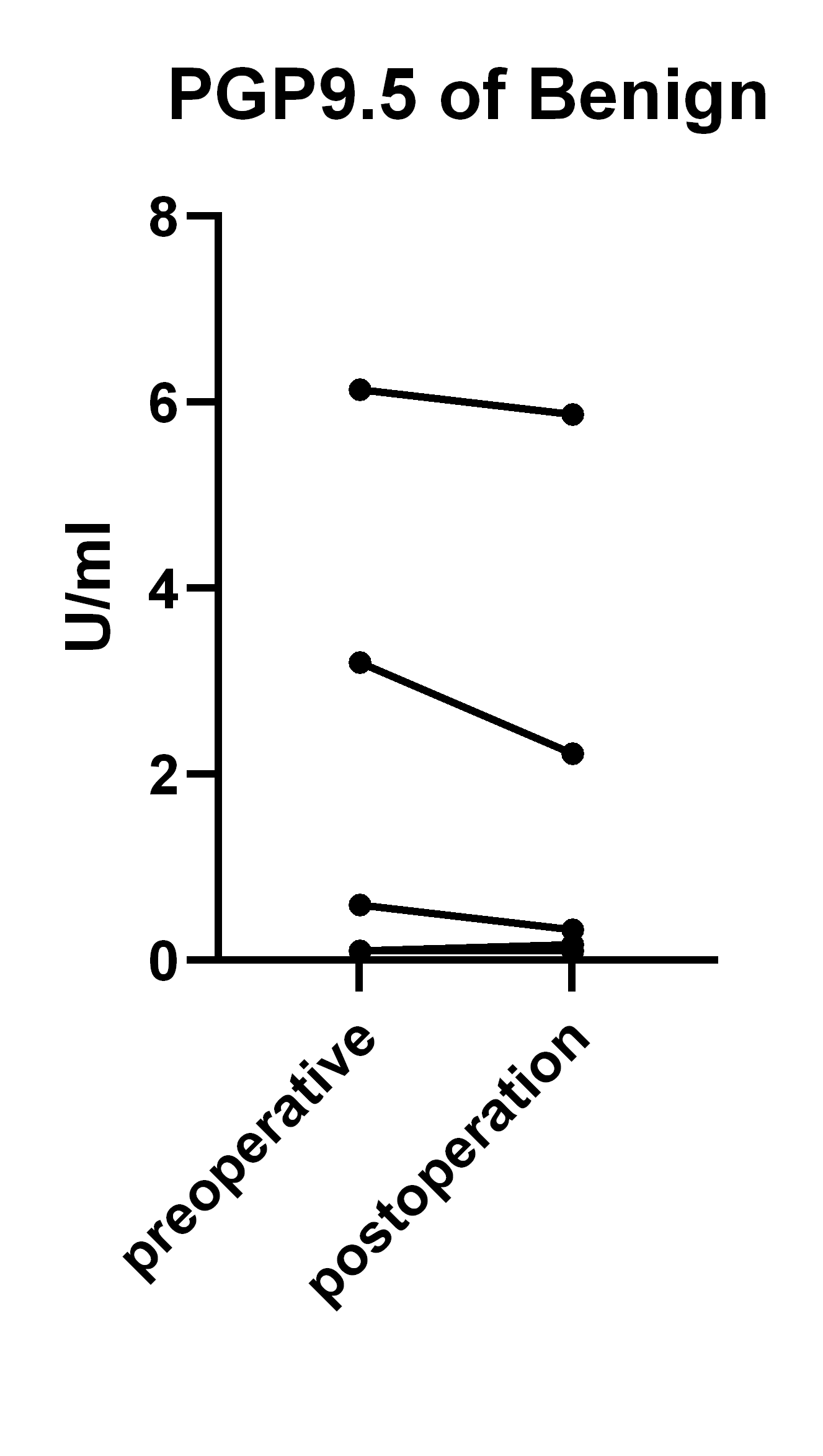

Supplement: Supplementary file 1 — Supplementary Material 1 [file 12890_2024_3060_MOESM1_ESM.png]

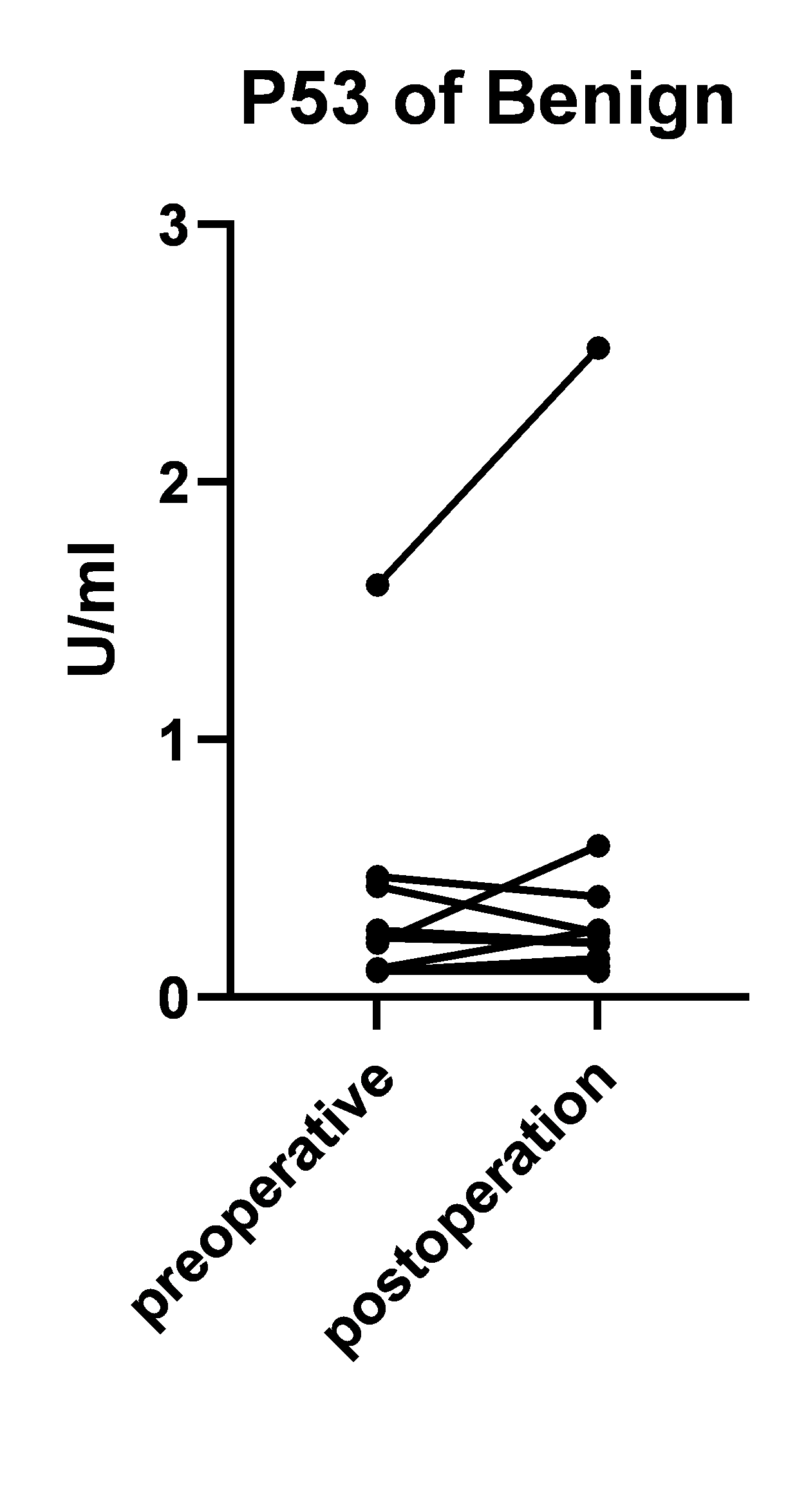

Supplement: Supplementary file 2 — Supplementary Material 2 [file 12890_2024_3060_MOESM2_ESM.png]

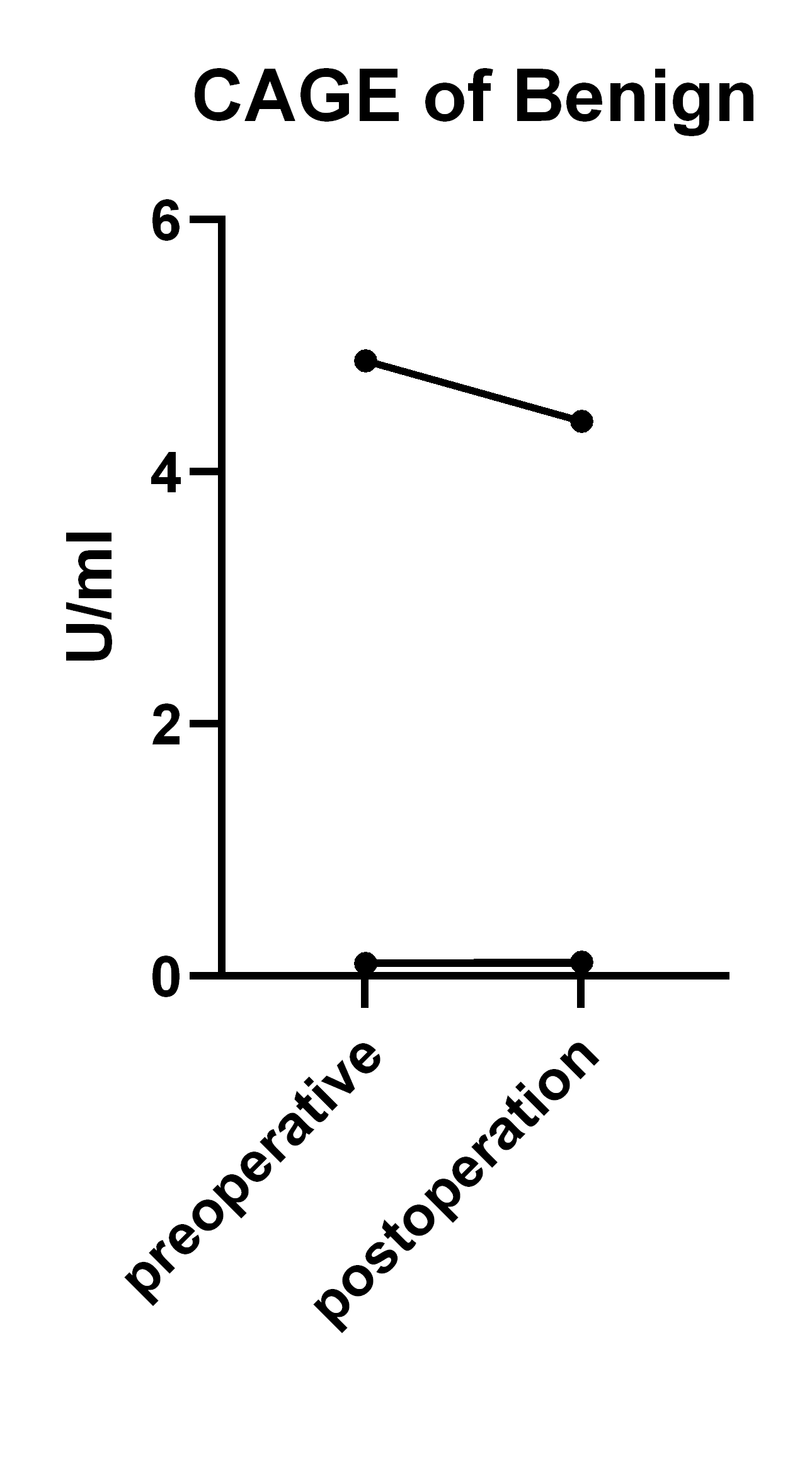

Supplement: Supplementary file 3 — Supplementary Material 3 [file 12890_2024_3060_MOESM3_ESM.png]

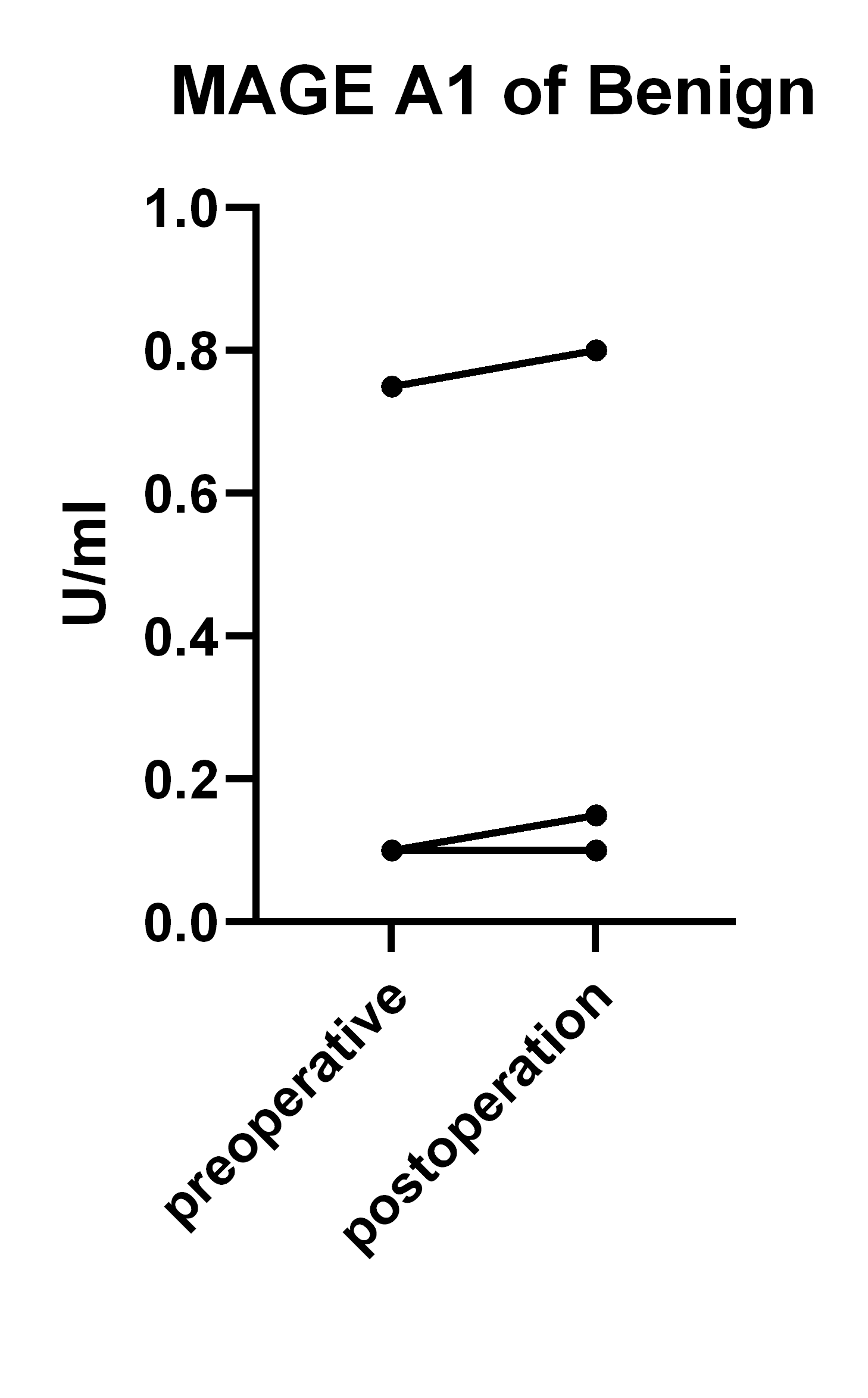

Supplement: Supplementary file 4 — Supplementary Material 4 [file 12890_2024_3060_MOESM4_ESM.png]

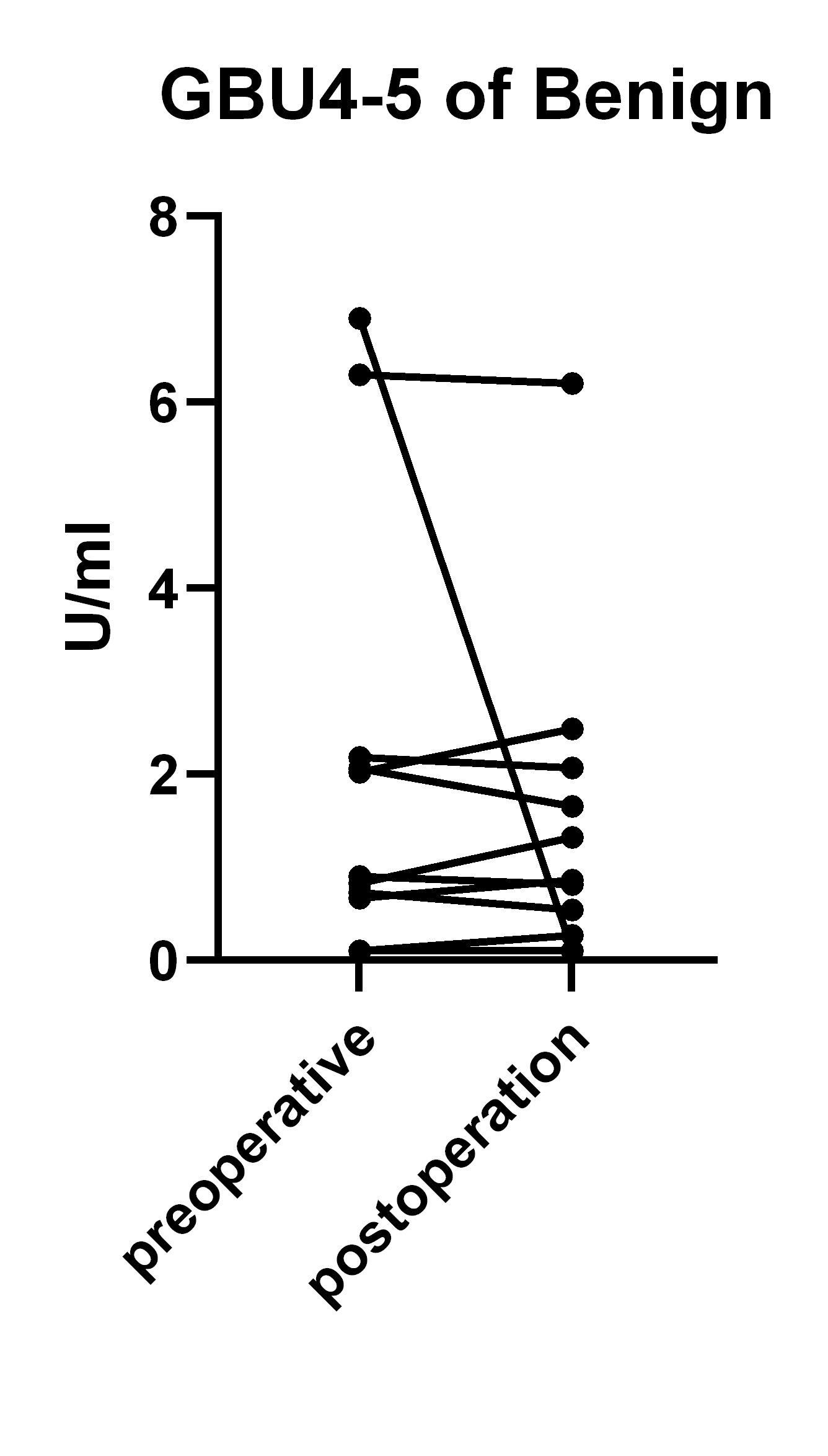

Supplement: Supplementary file 5 — Supplementary Material 5 [file 12890_2024_3060_MOESM5_ESM.png]

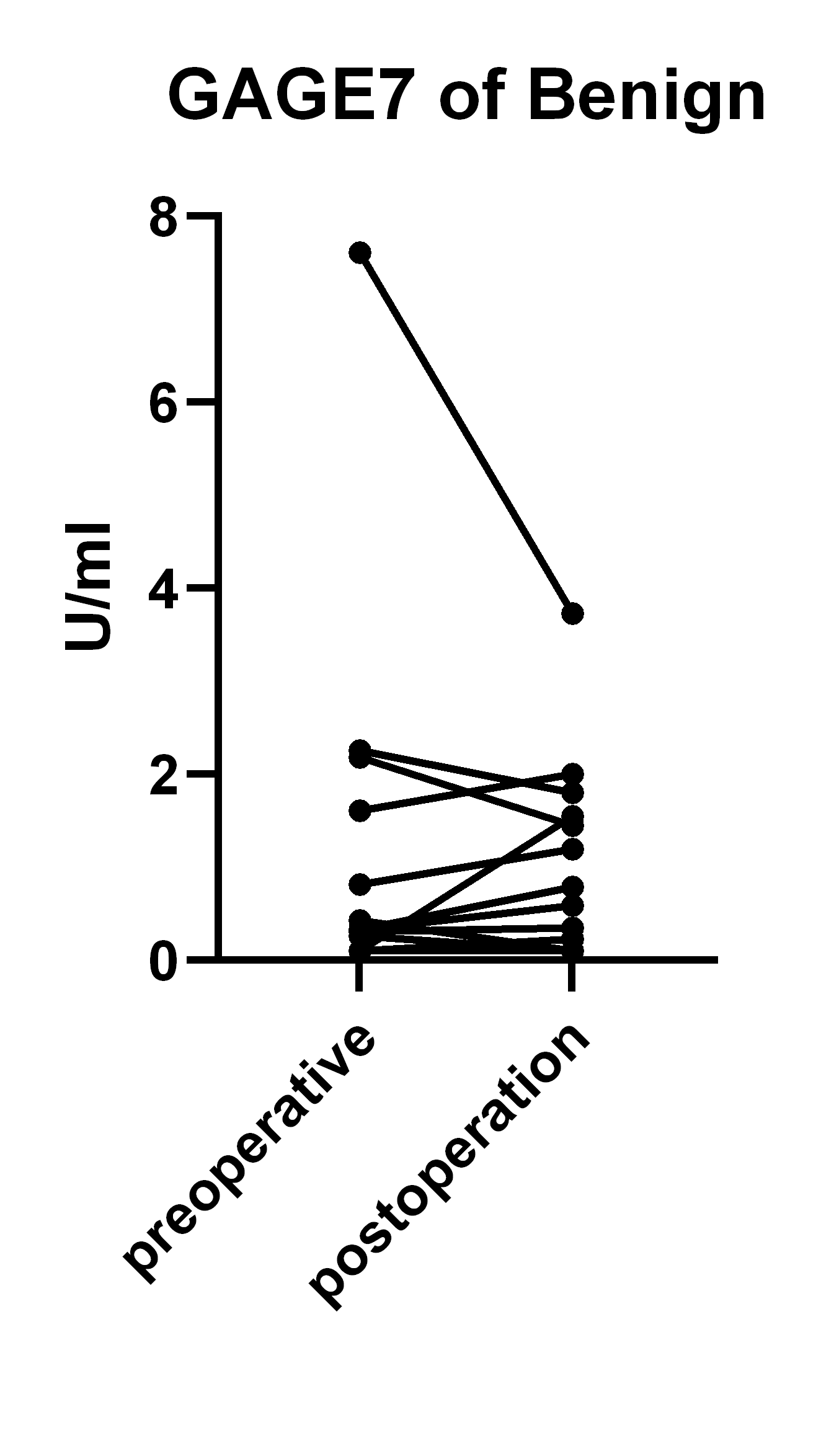

Supplement: Supplementary file 6 — Supplementary Material 6 [file 12890_2024_3060_MOESM6_ESM.png]

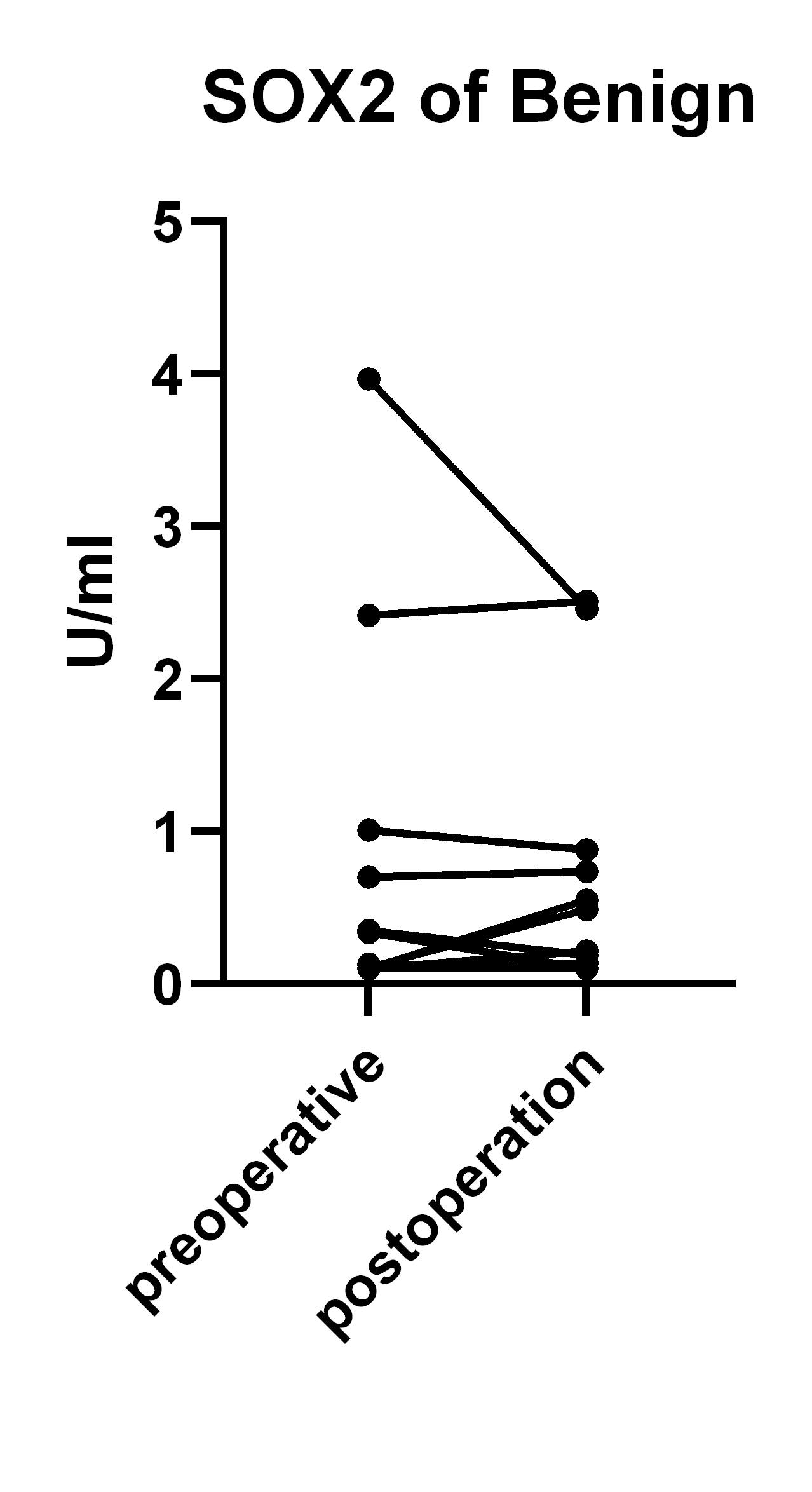

Supplement: Supplementary file 7 — Supplementary Material 7 [file 12890_2024_3060_MOESM7_ESM.png]

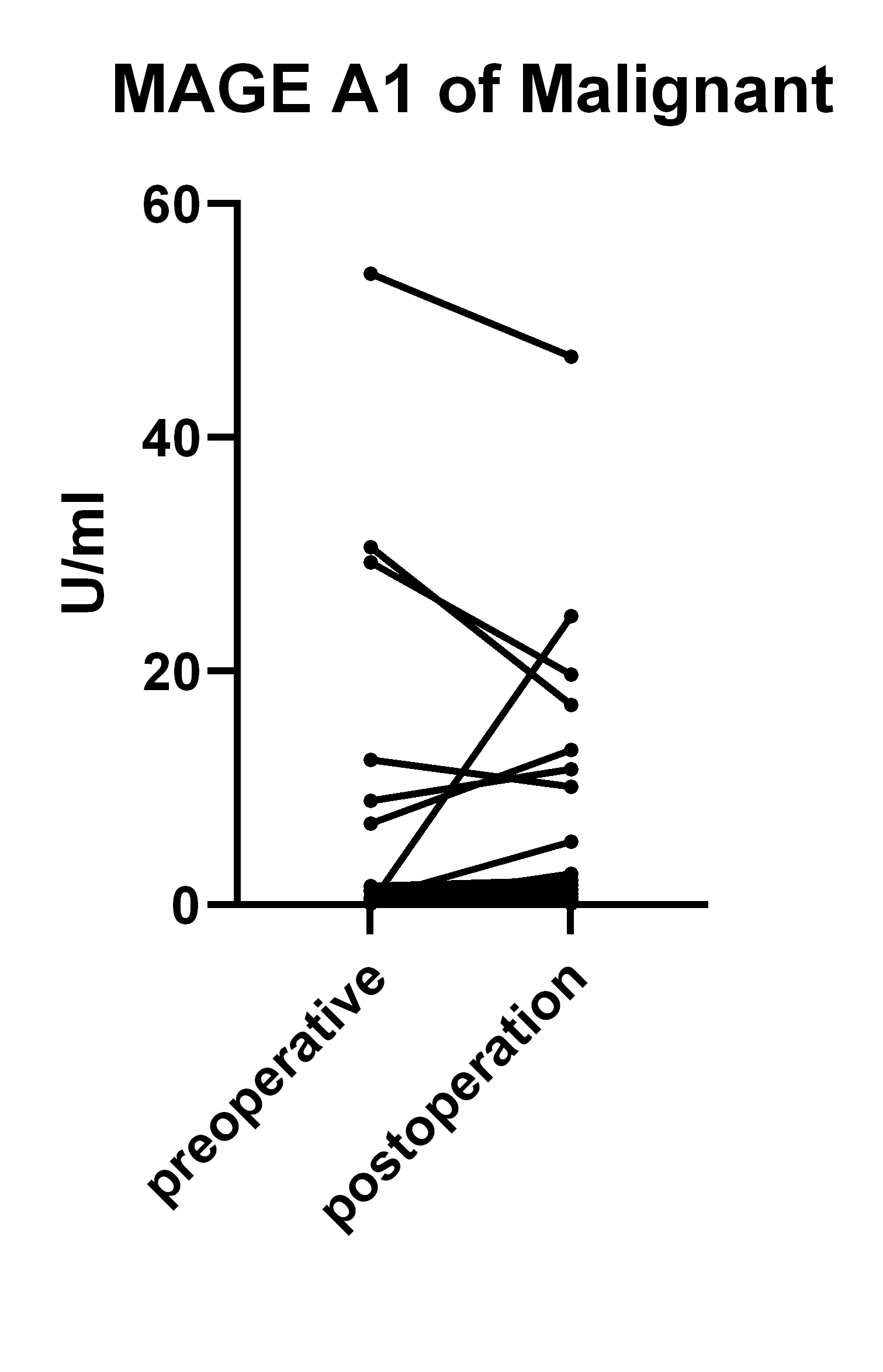

Supplement: Supplementary file 8 — Supplementary Material 8 [file 12890_2024_3060_MOESM8_ESM.png]

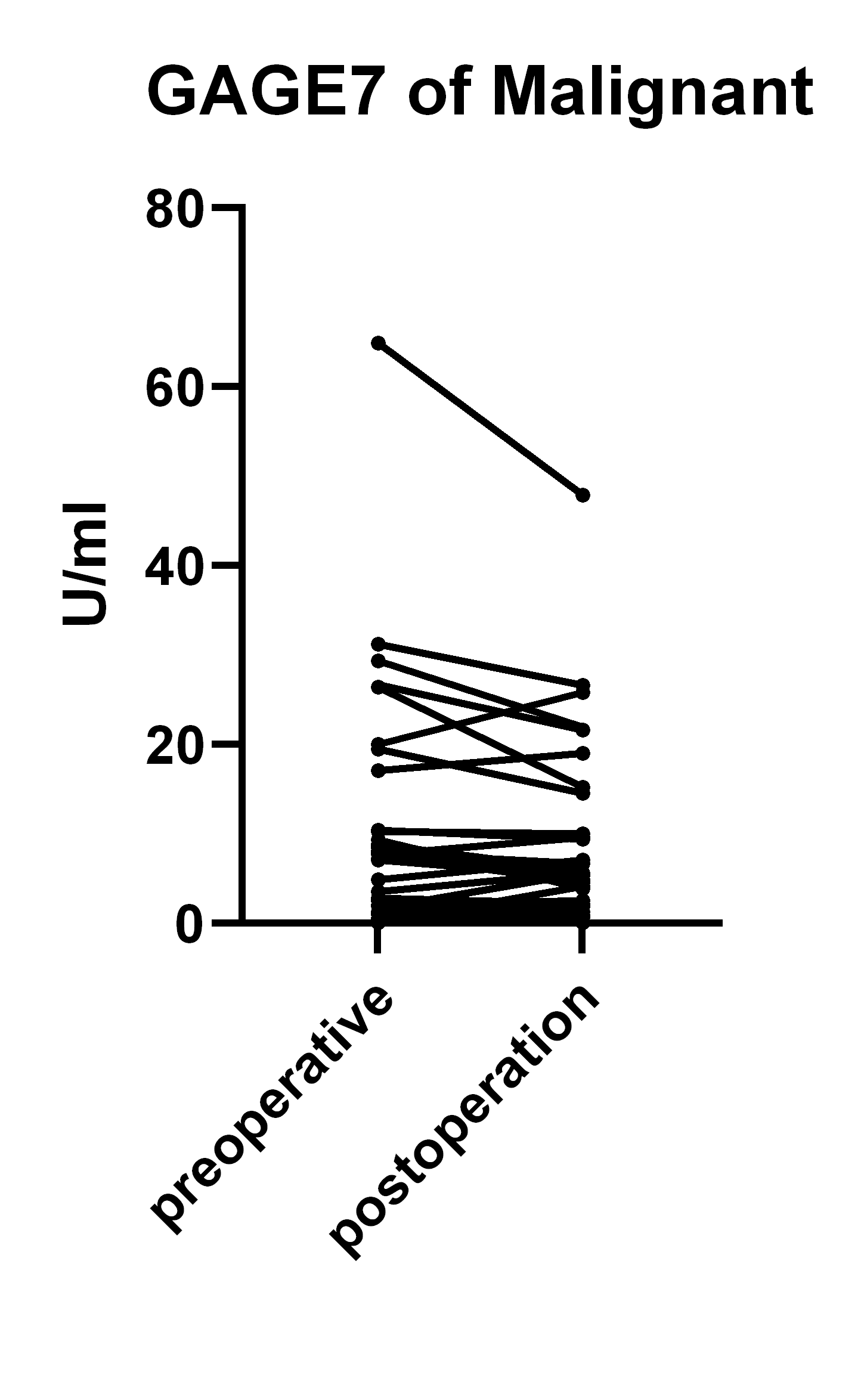

Supplement: Supplementary file 9 — Supplementary Material 9 [file 12890_2024_3060_MOESM9_ESM.png]

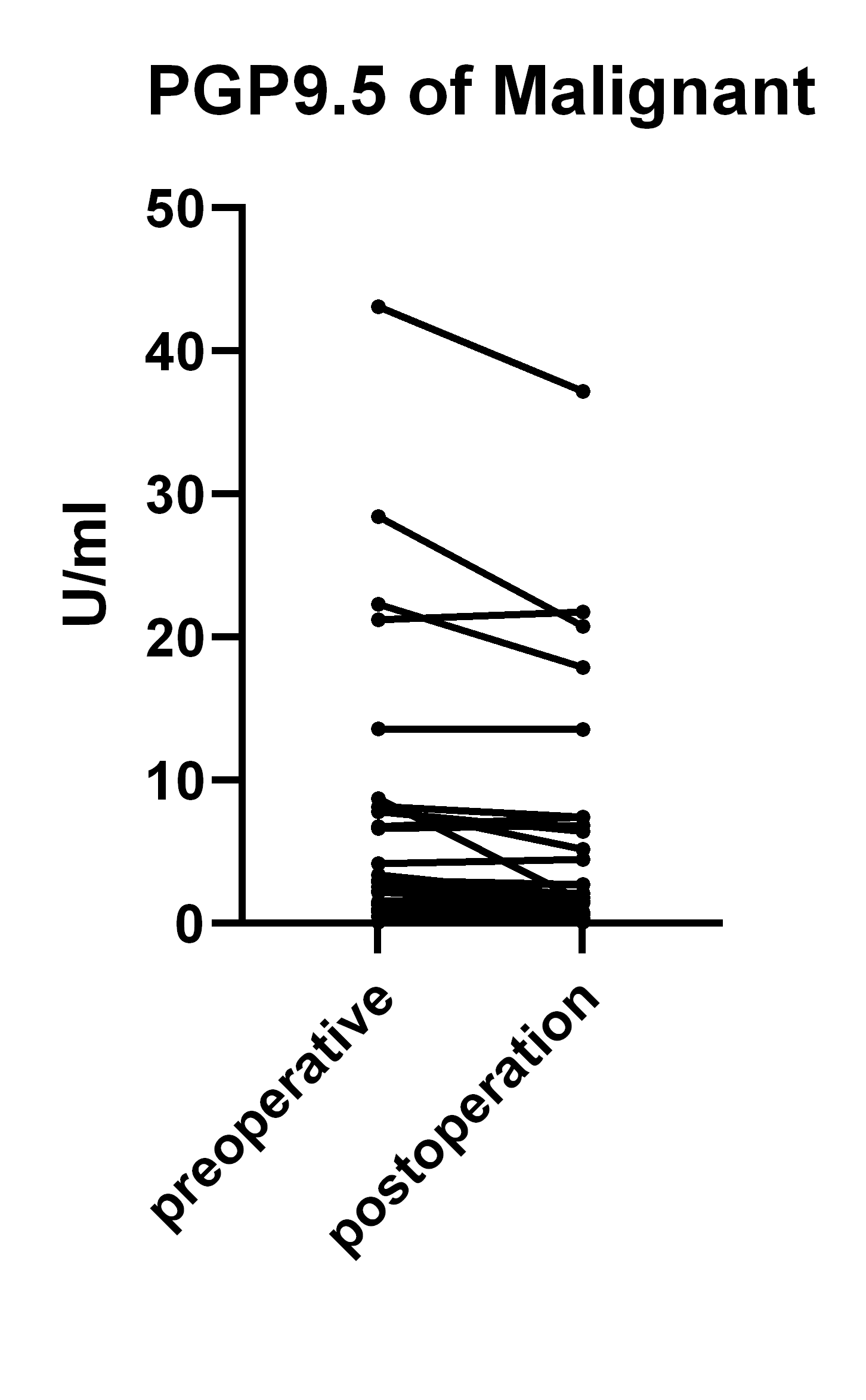

Supplement: Supplementary file 10 — Supplementary Material 10 [file 12890_2024_3060_MOESM10_ESM.png]

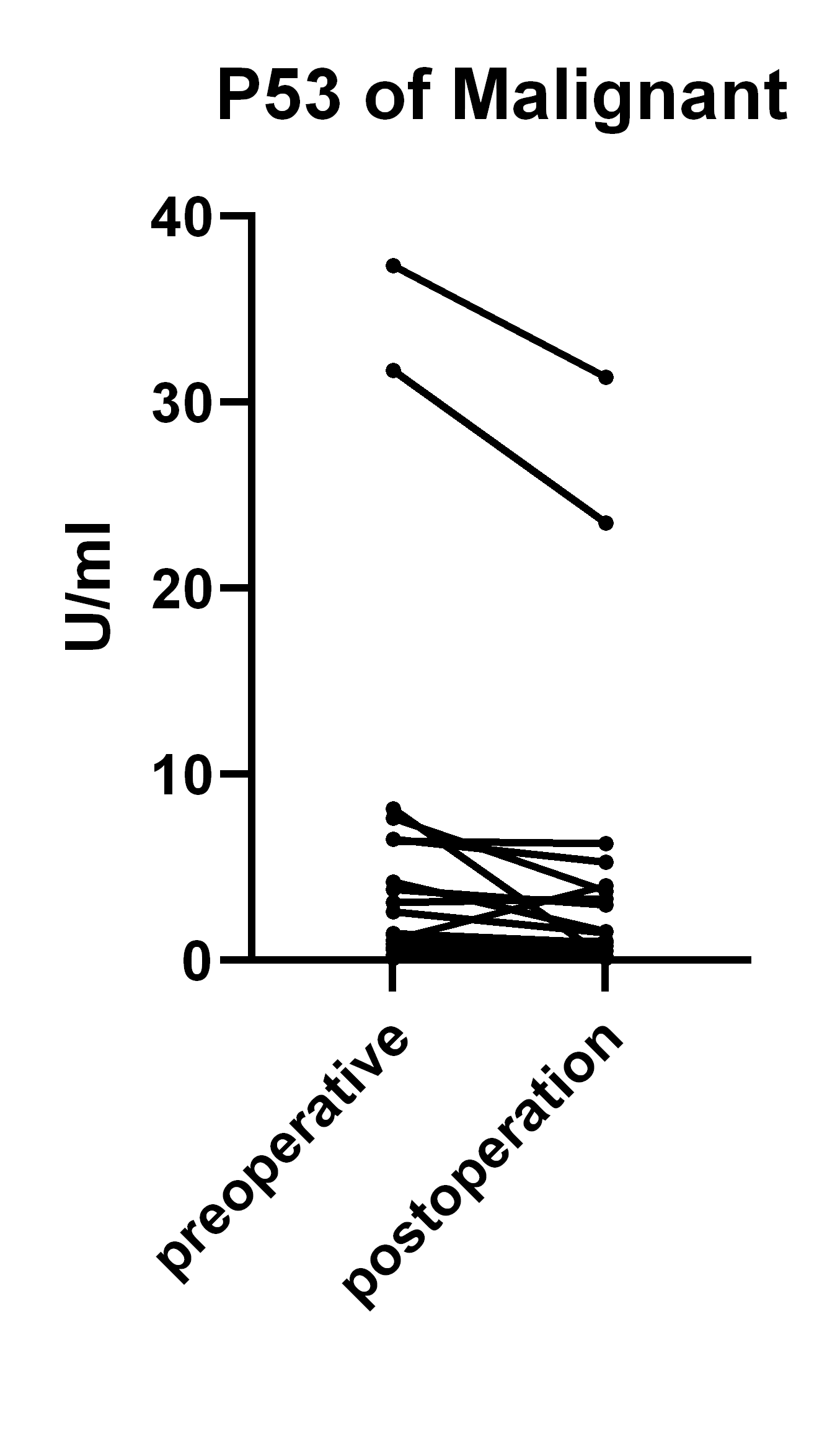

Supplement: Supplementary file 11 — Supplementary Material 11 [file 12890_2024_3060_MOESM11_ESM.png]

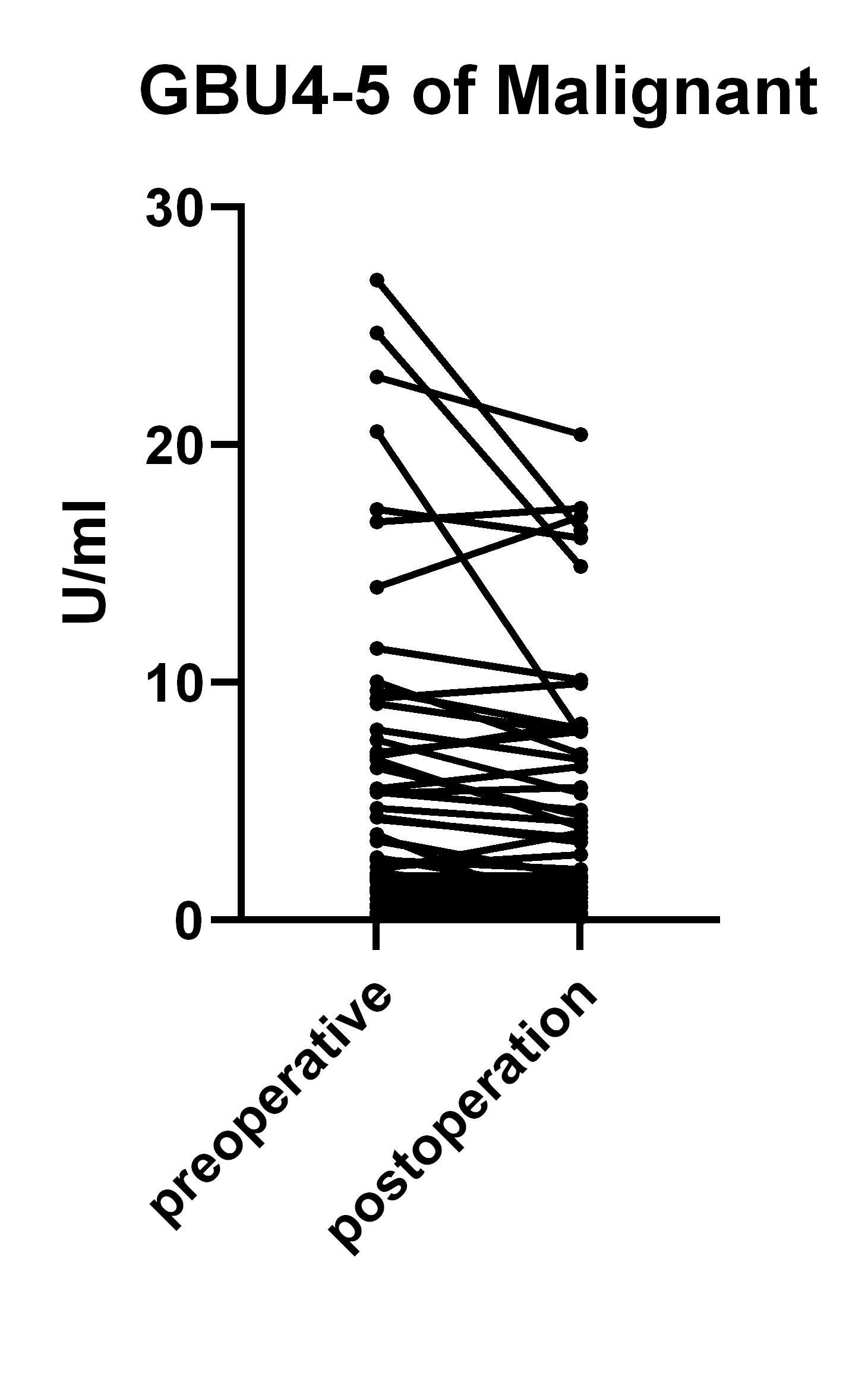

Supplement: Supplementary file 12 — Supplementary Material 12 [file 12890_2024_3060_MOESM12_ESM.png]

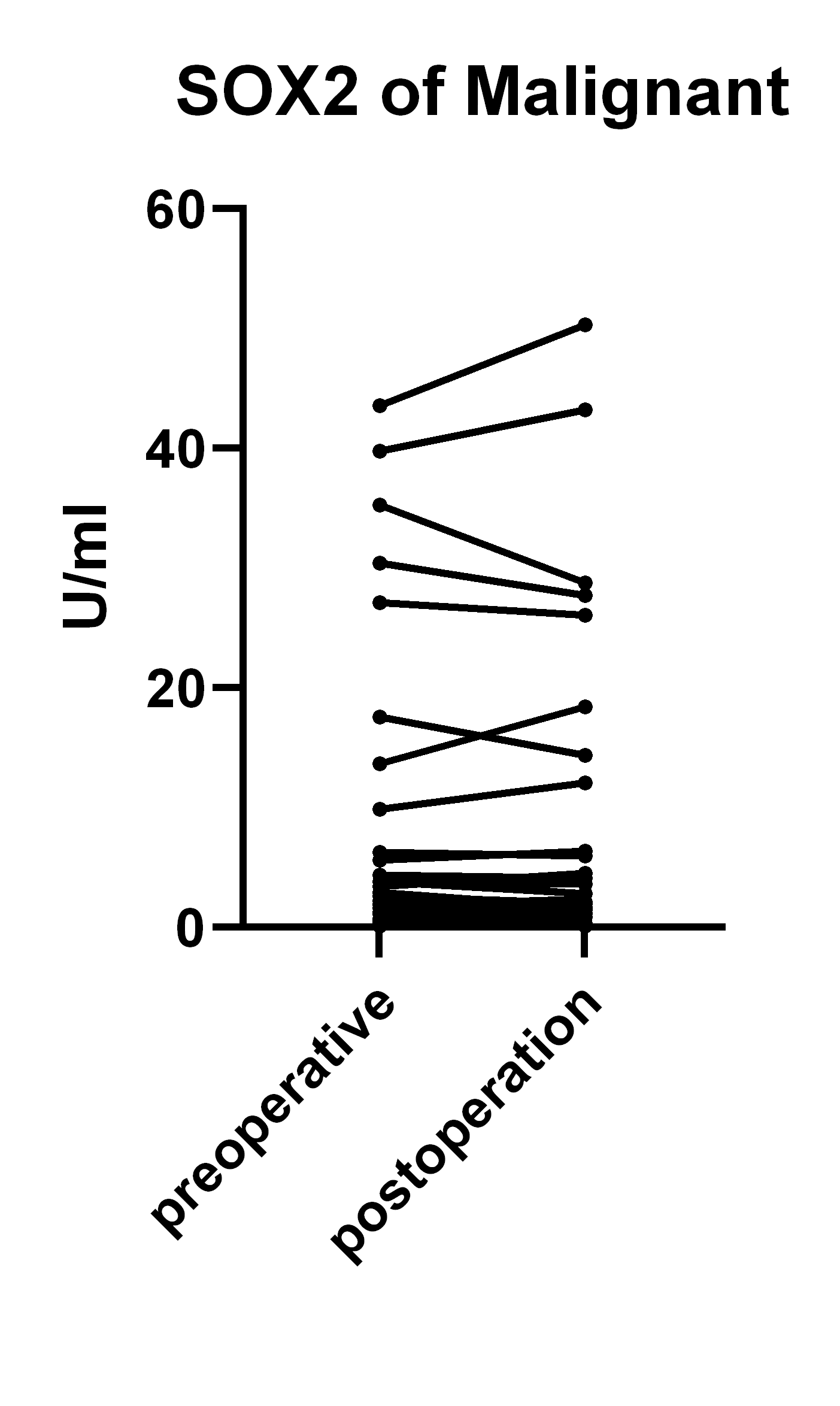

Supplement: Supplementary file 13 — Supplementary Material 13 [file 12890_2024_3060_MOESM13_ESM.png]

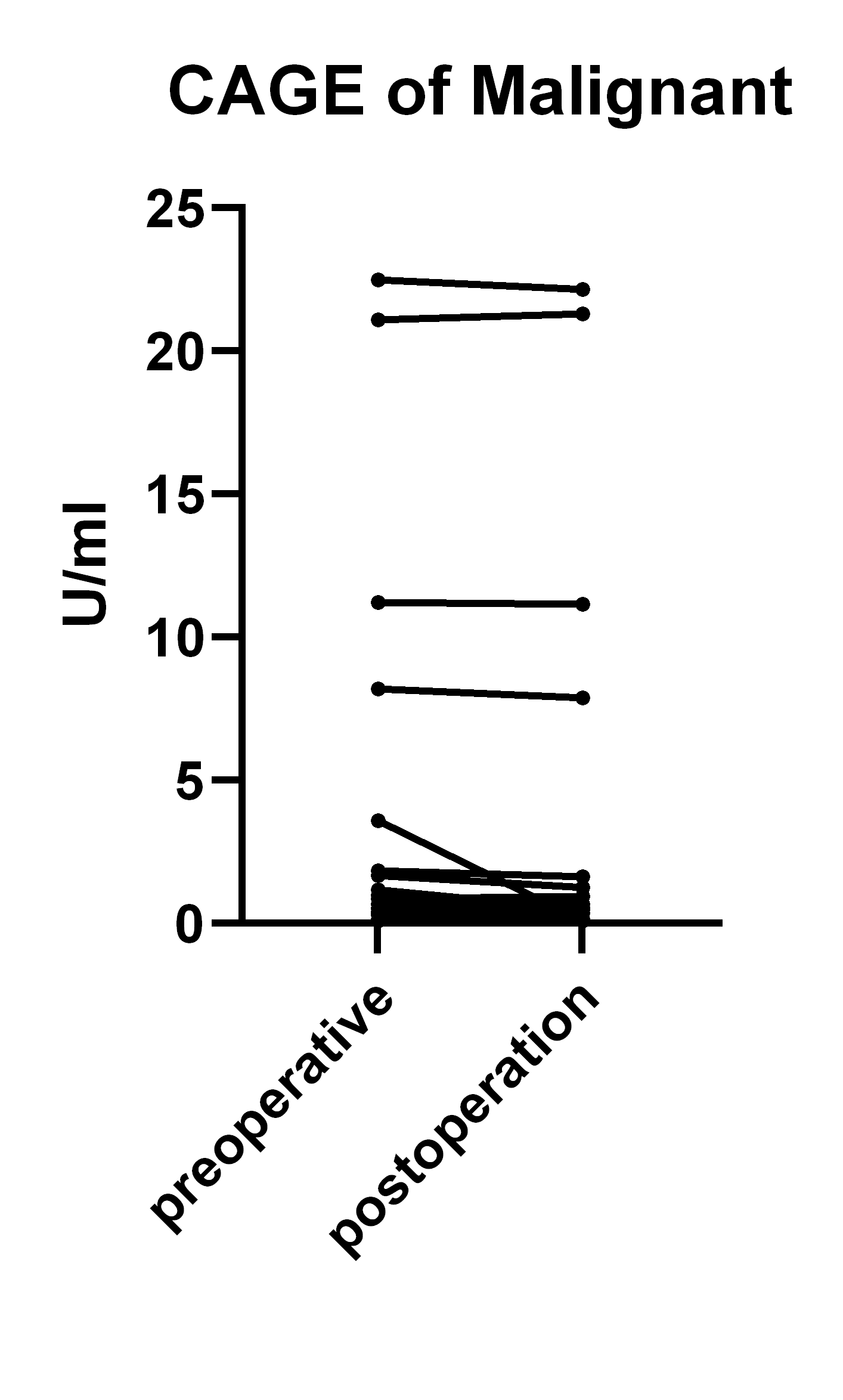

Supplement: Supplementary file 14 — Supplementary Material 14 [file 12890_2024_3060_MOESM14_ESM.png]

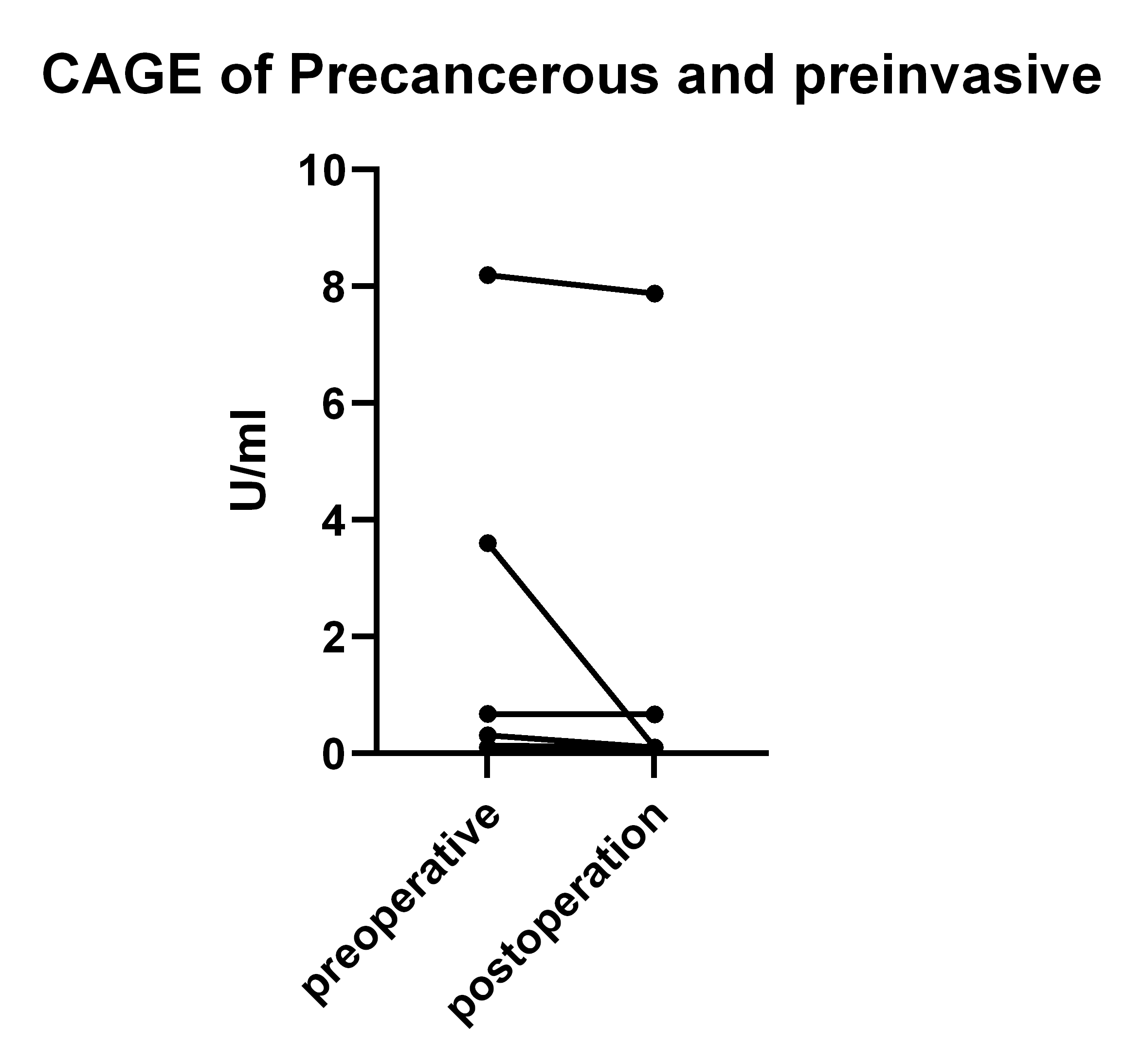

Supplement: Supplementary file 15 — Supplementary Material 15 [file 12890_2024_3060_MOESM15_ESM.png]

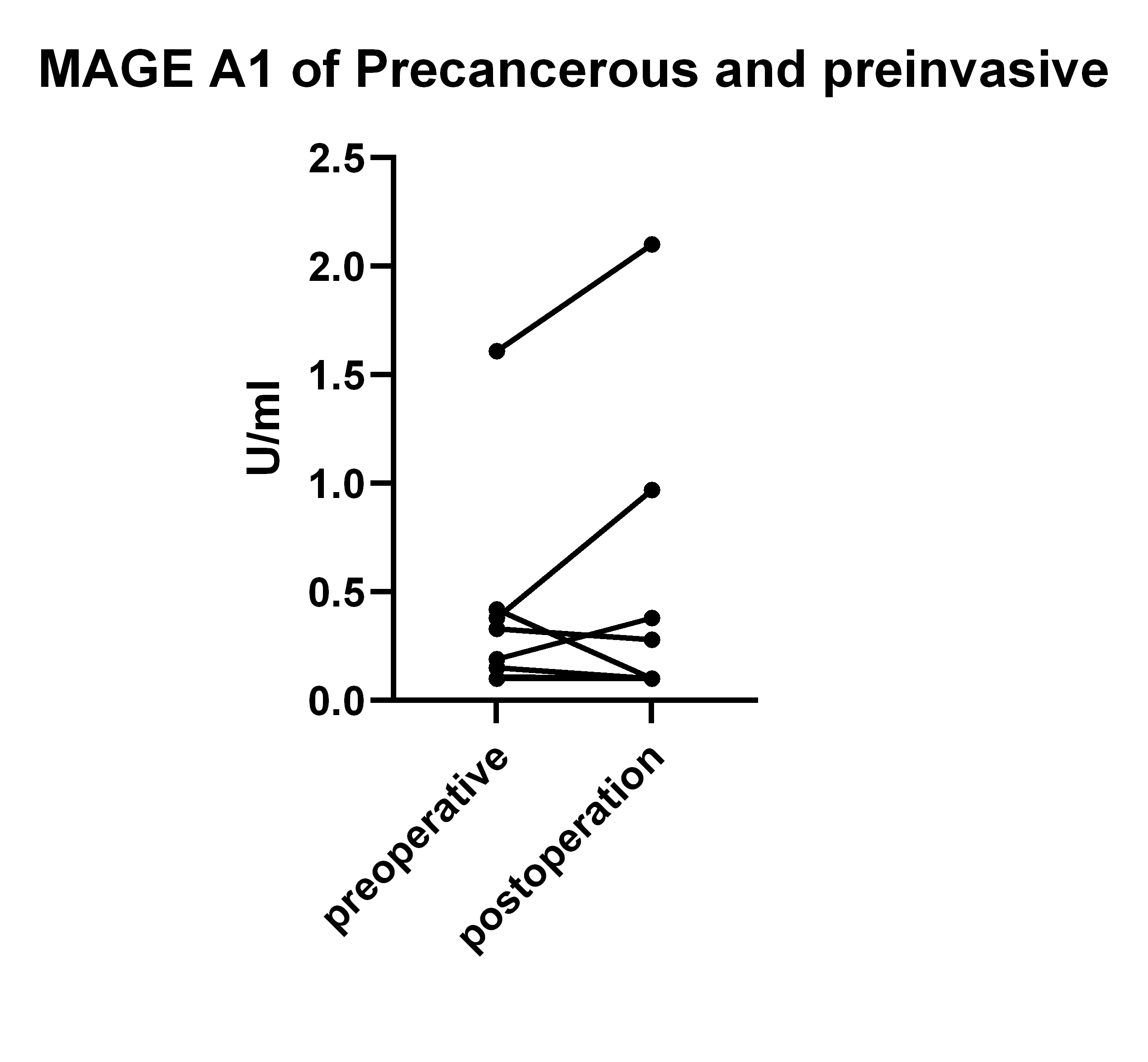

Supplement: Supplementary file 16 — Supplementary Material 16 [file 12890_2024_3060_MOESM16_ESM.png]

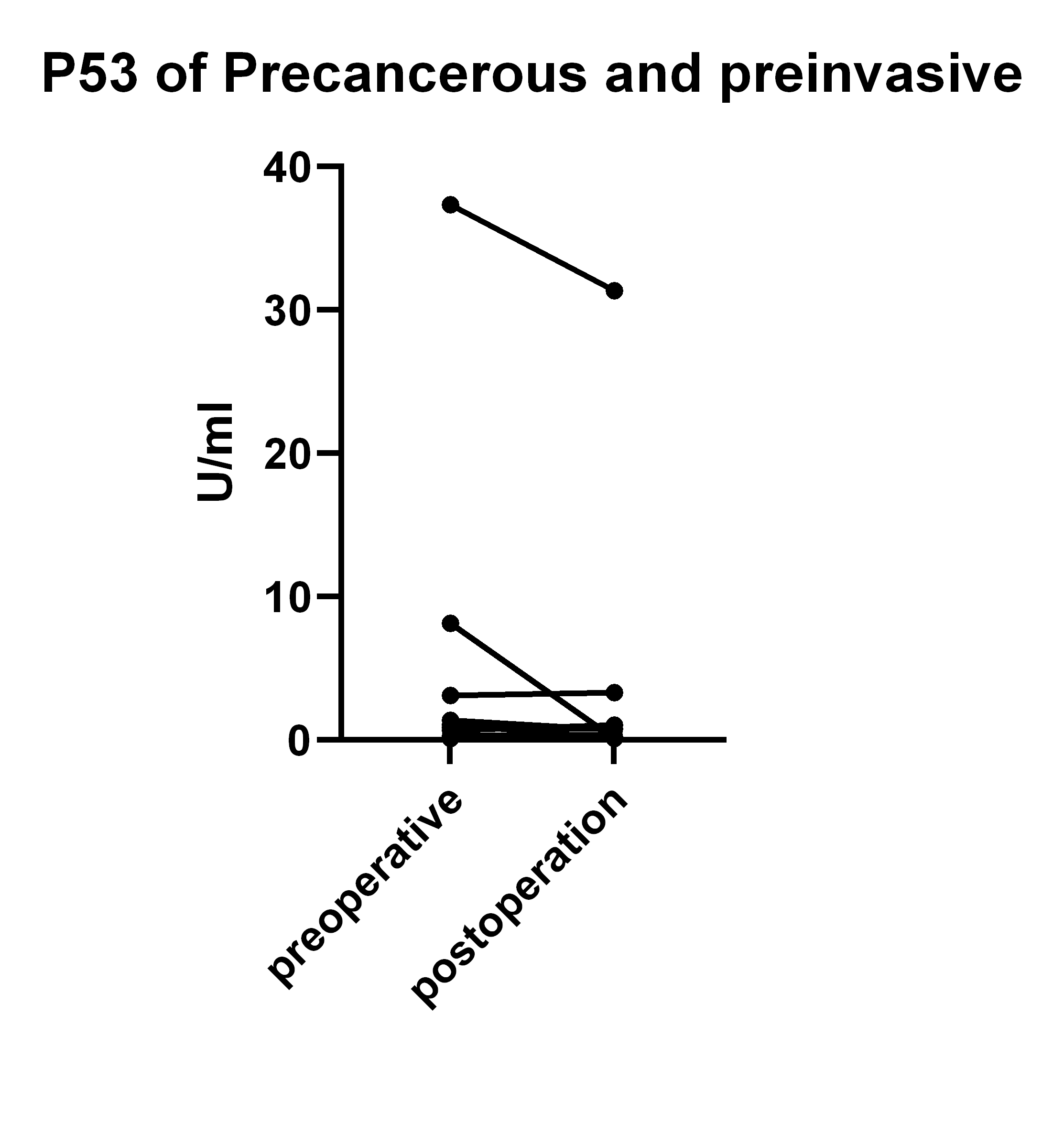

Supplement: Supplementary file 17 — Supplementary Material 17 [file 12890_2024_3060_MOESM17_ESM.png]

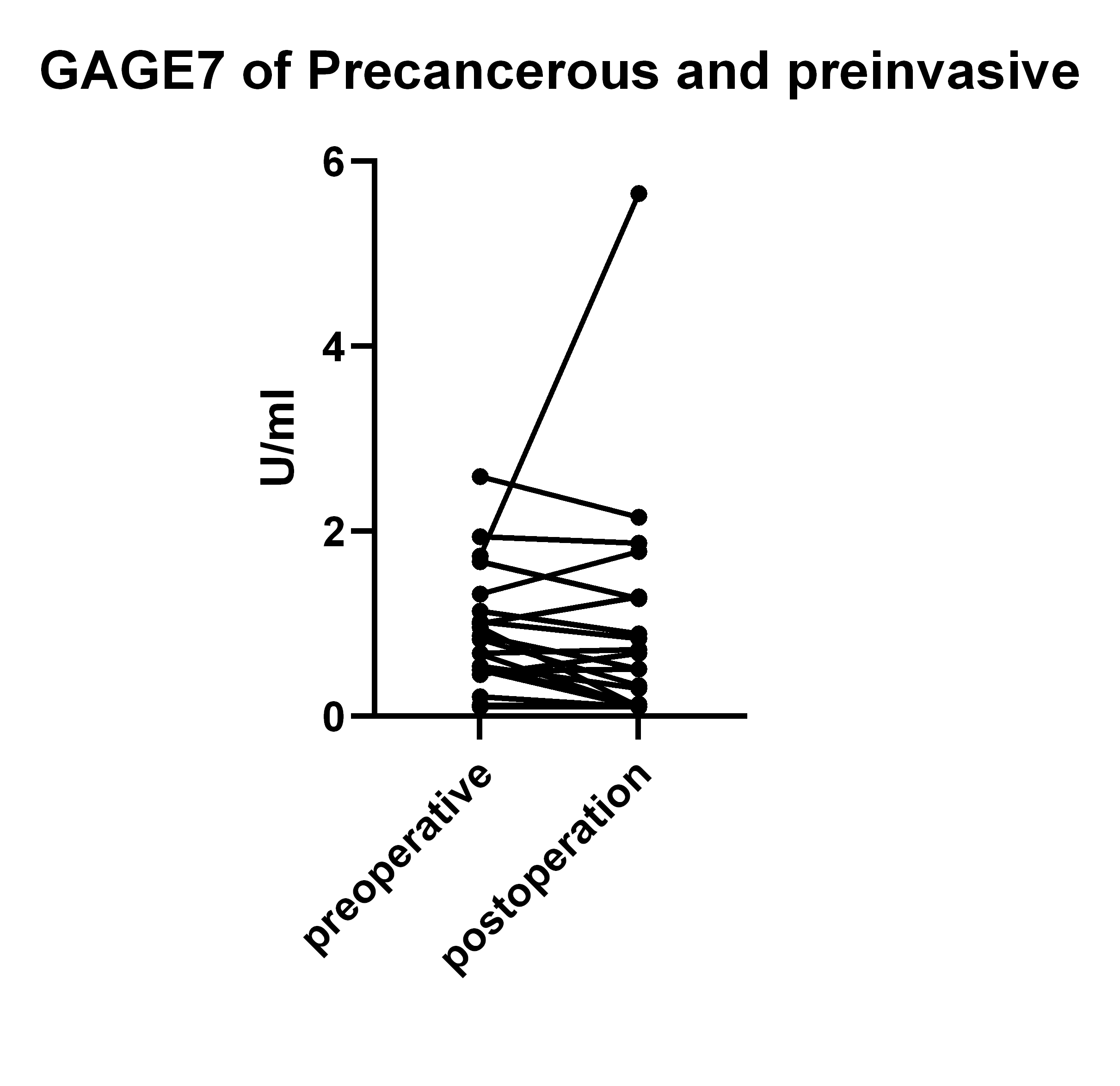

Supplement: Supplementary file 18 — Supplementary Material 18 [file 12890_2024_3060_MOESM18_ESM.png]

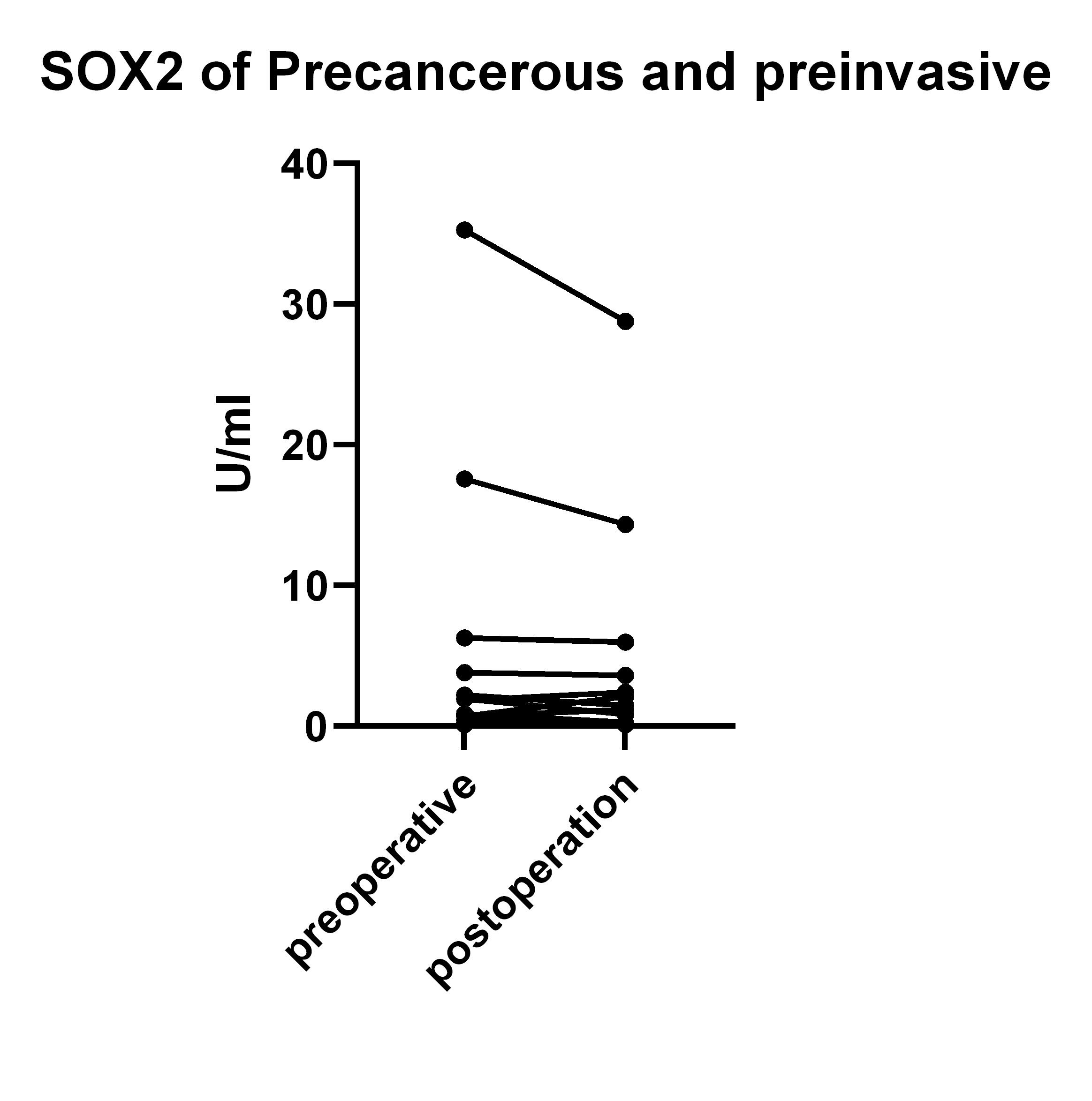

Supplement: Supplementary file 19 — Supplementary Material 19 [file 12890_2024_3060_MOESM19_ESM.png]

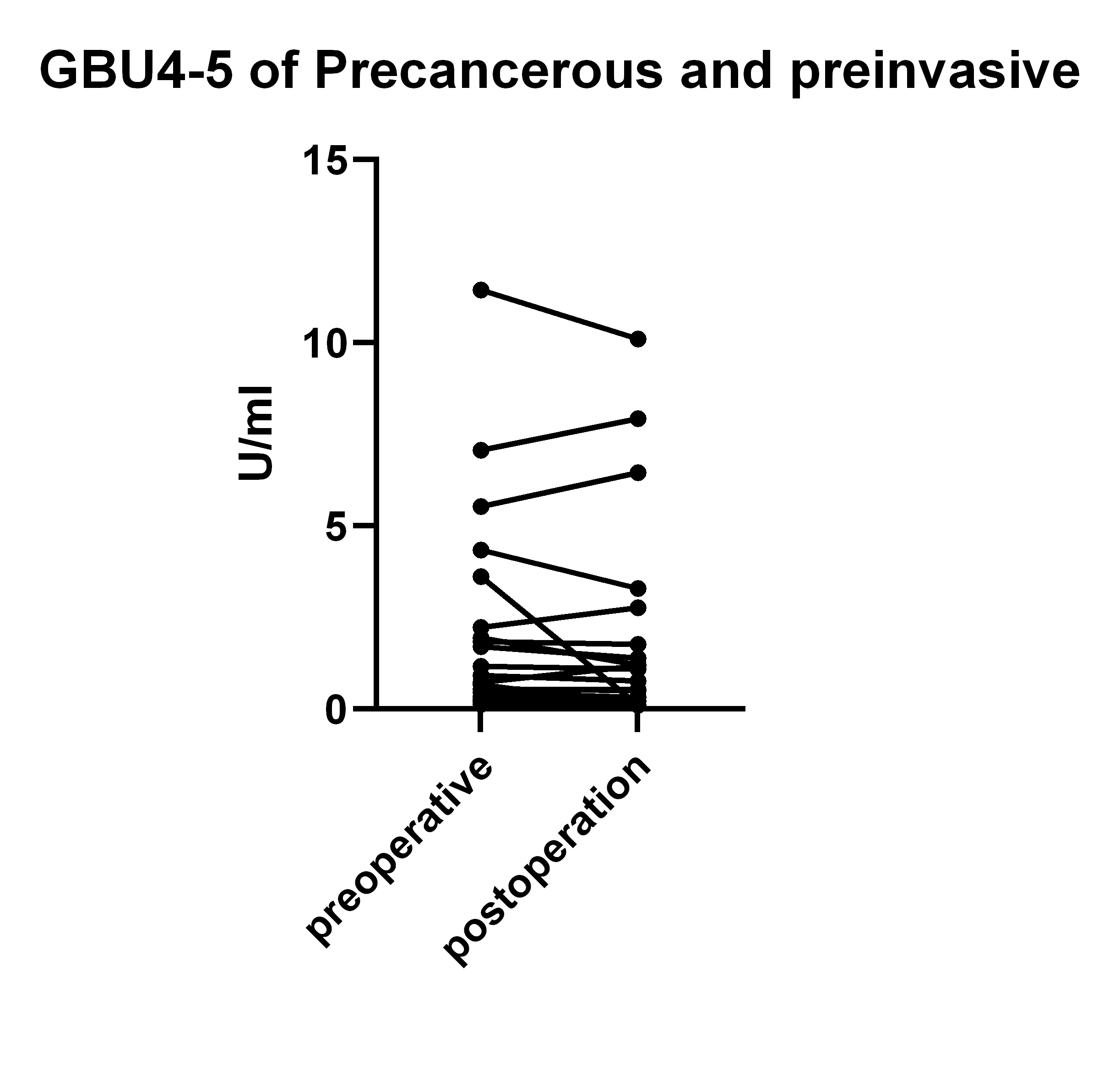

Supplement: Supplementary file 20 — Supplementary Material 20 [file 12890_2024_3060_MOESM20_ESM.png]

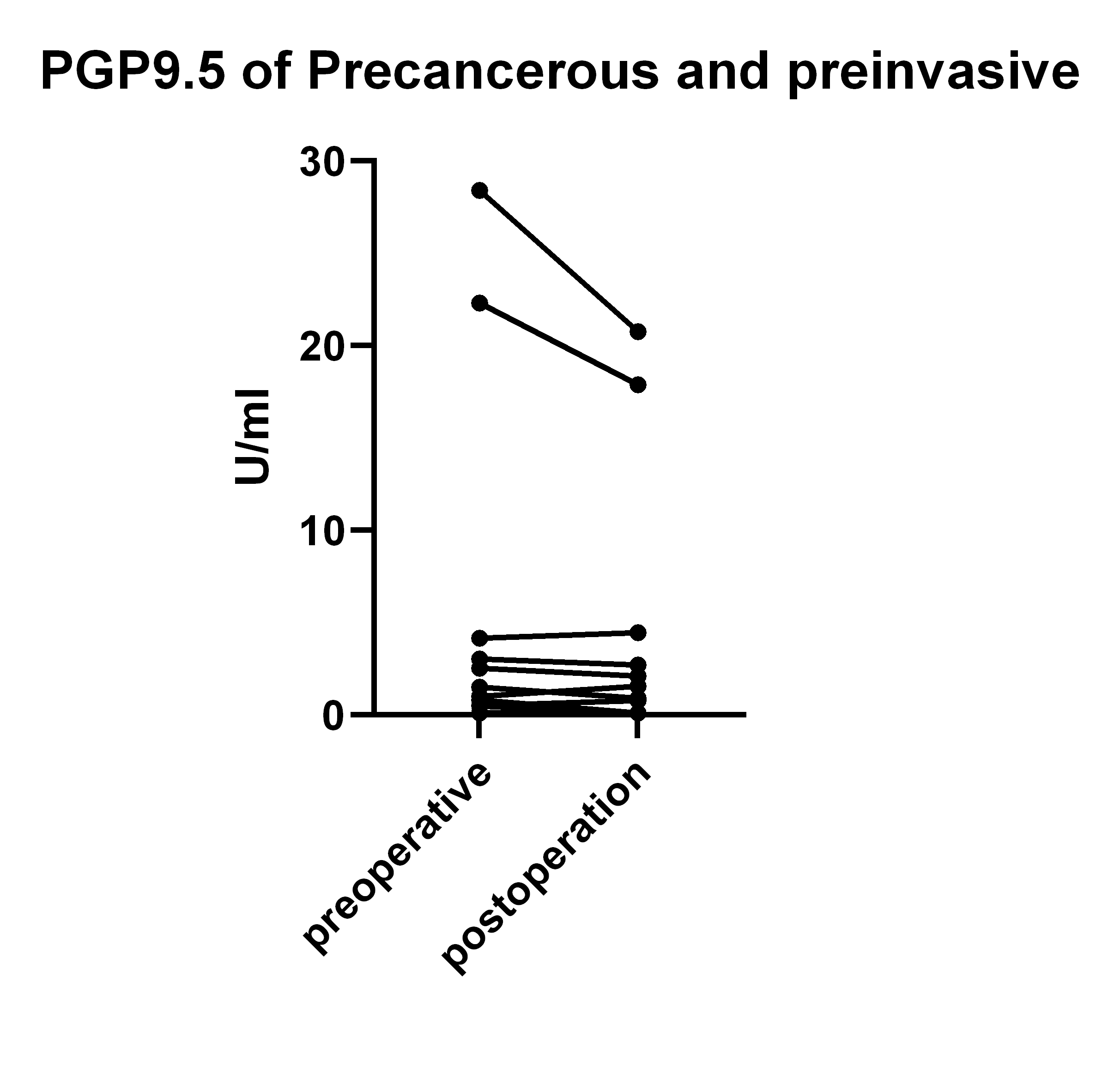

Supplement: Supplementary file 21 — Supplementary Material 21 [file 12890_2024_3060_MOESM21_ESM.png]

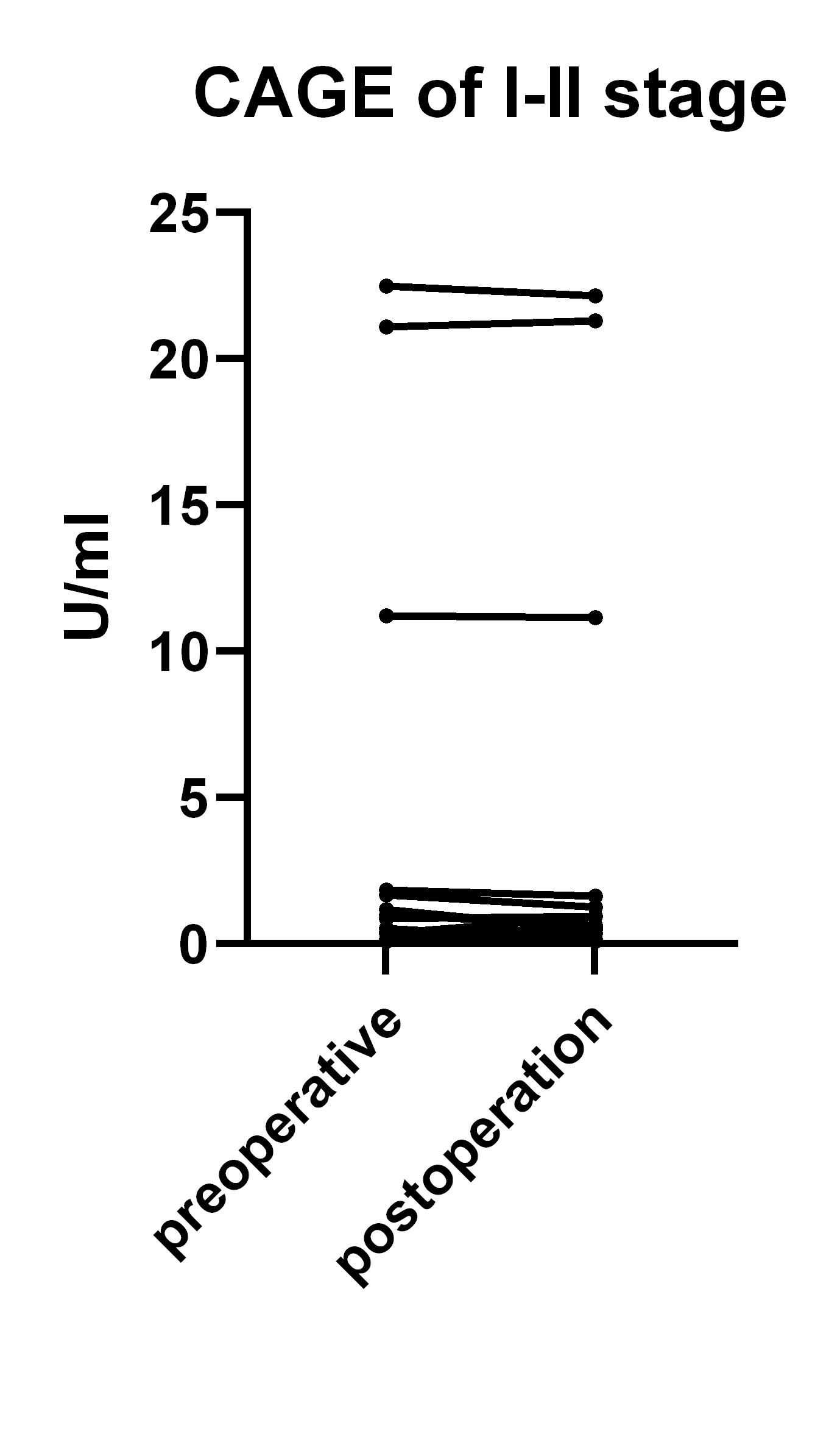

Supplement: Supplementary file 22 — Supplementary Material 22 [file 12890_2024_3060_MOESM22_ESM.png]

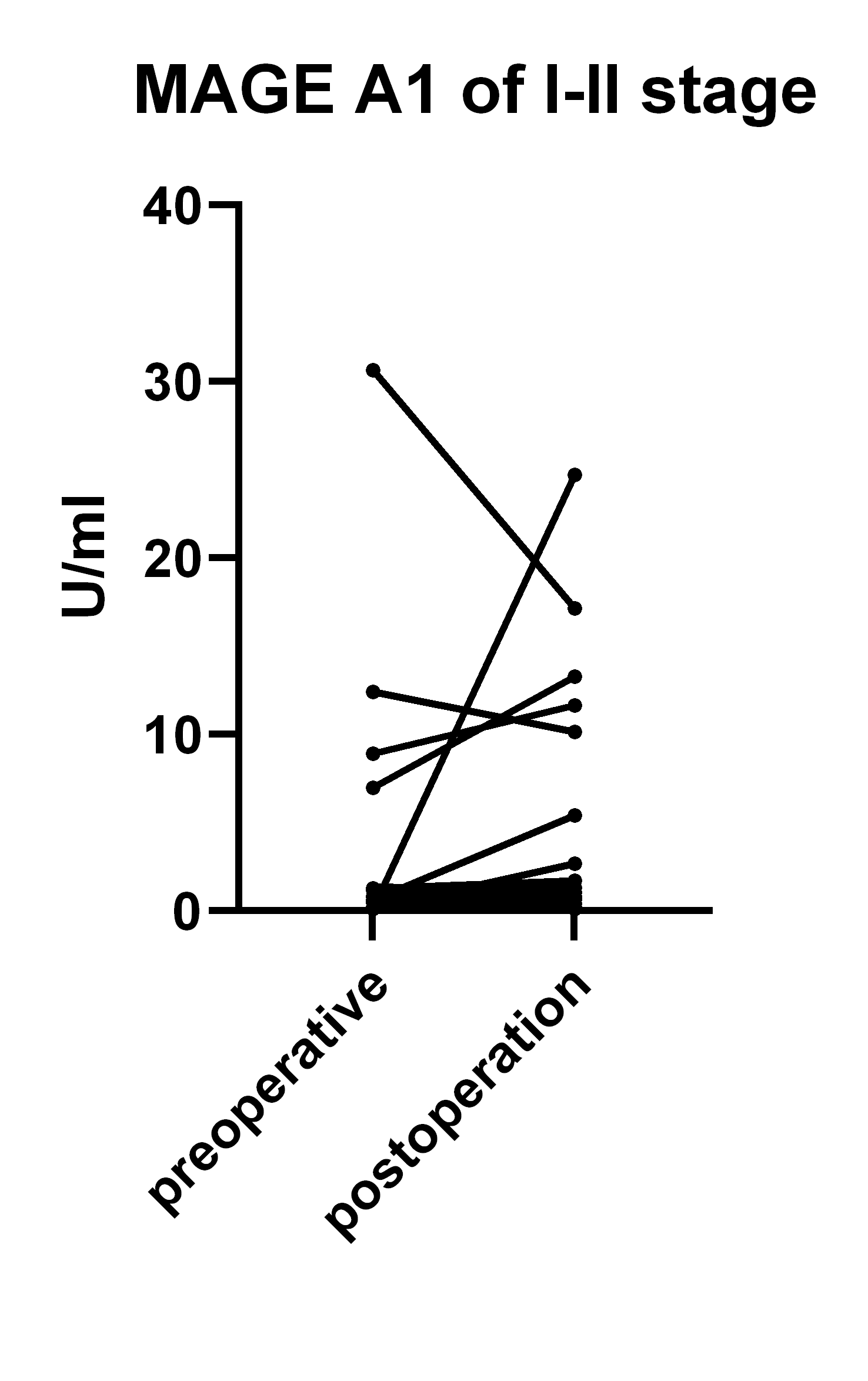

Supplement: Supplementary file 23 — Supplementary Material 23 [file 12890_2024_3060_MOESM23_ESM.png]

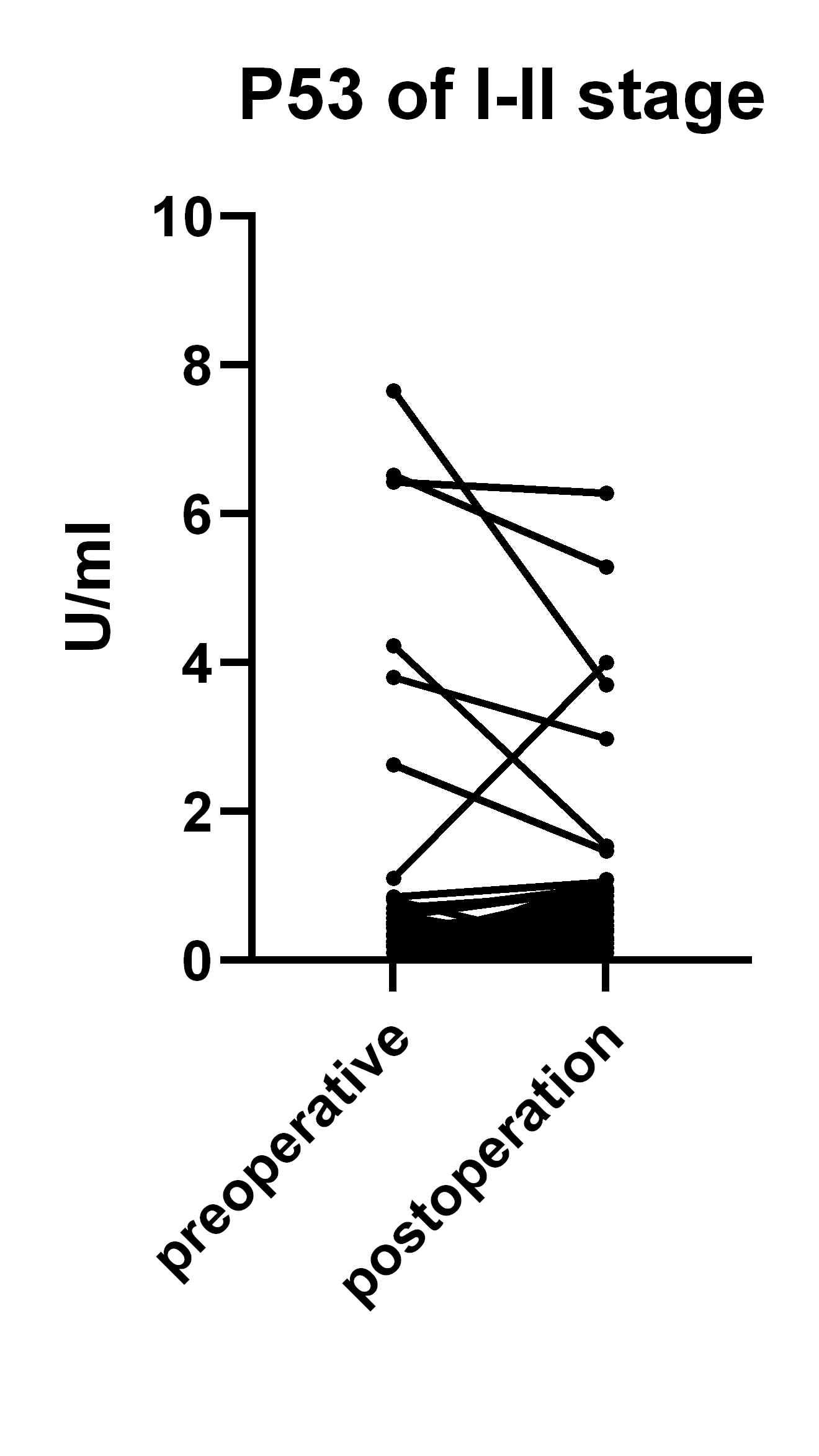

Supplement: Supplementary file 24 — Supplementary Material 24 [file 12890_2024_3060_MOESM24_ESM.png]

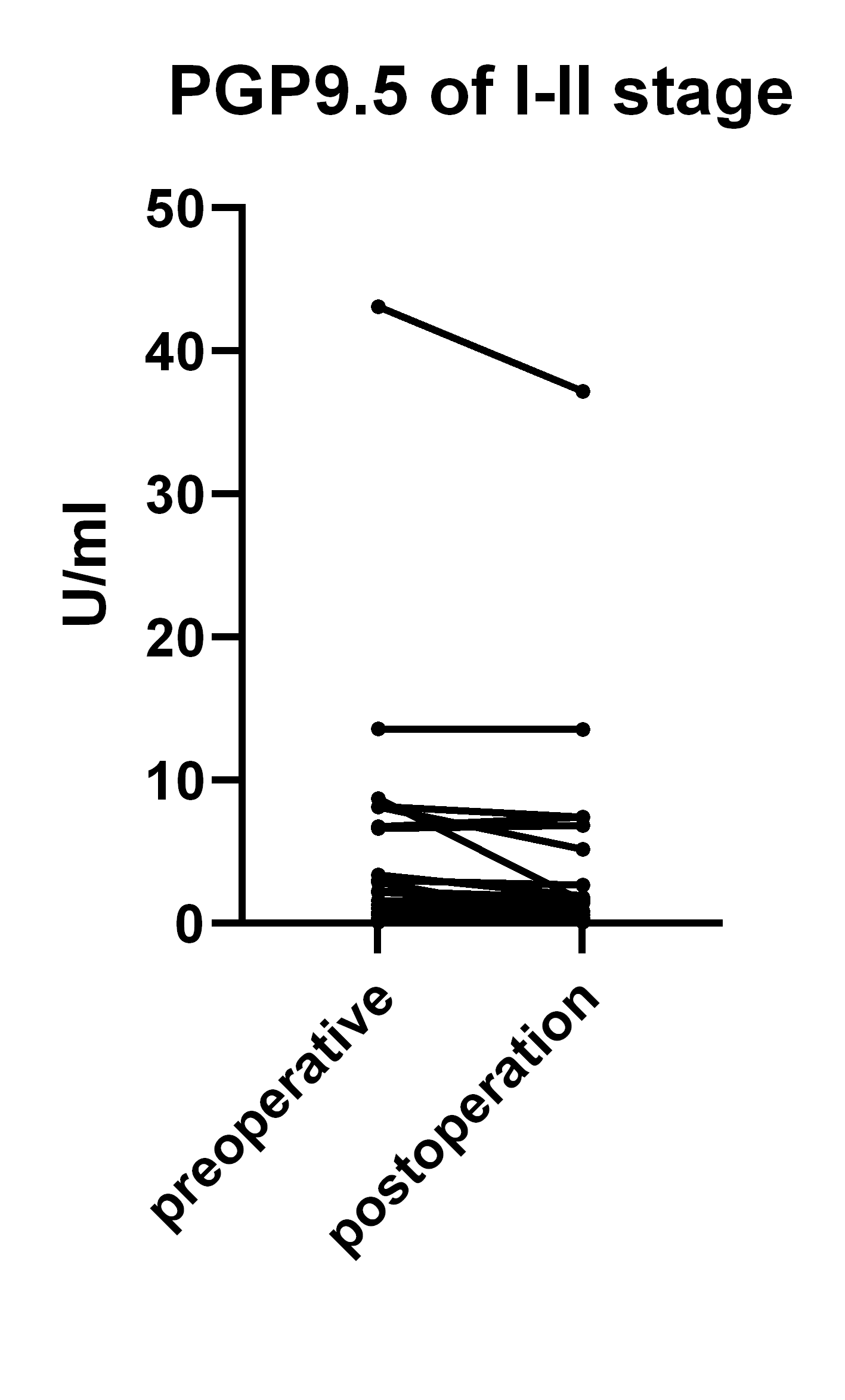

Supplement: Supplementary file 25 — Supplementary Material 25 [file 12890_2024_3060_MOESM25_ESM.png]

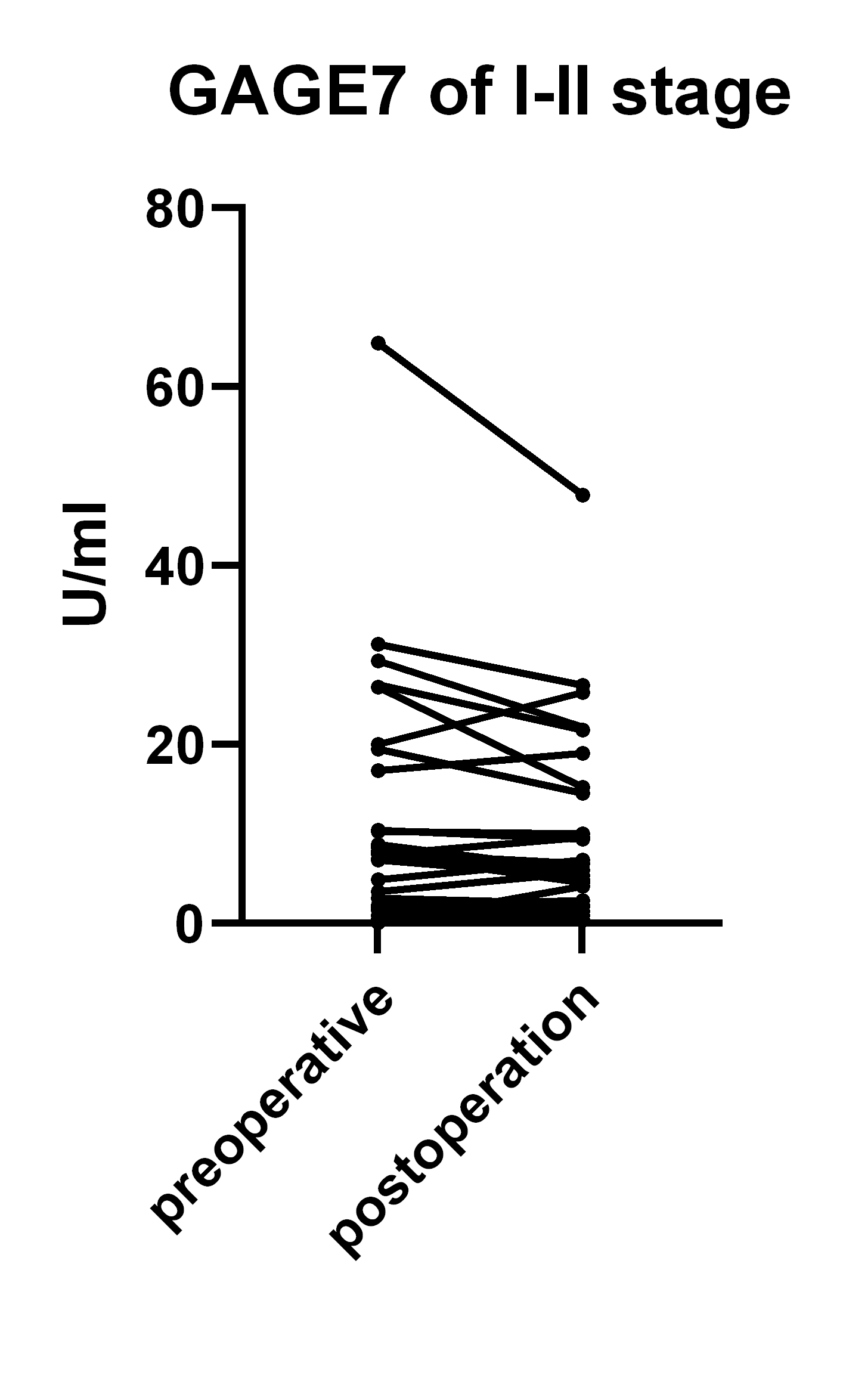

Supplement: Supplementary file 26 — Supplementary Material 26 [file 12890_2024_3060_MOESM26_ESM.png]

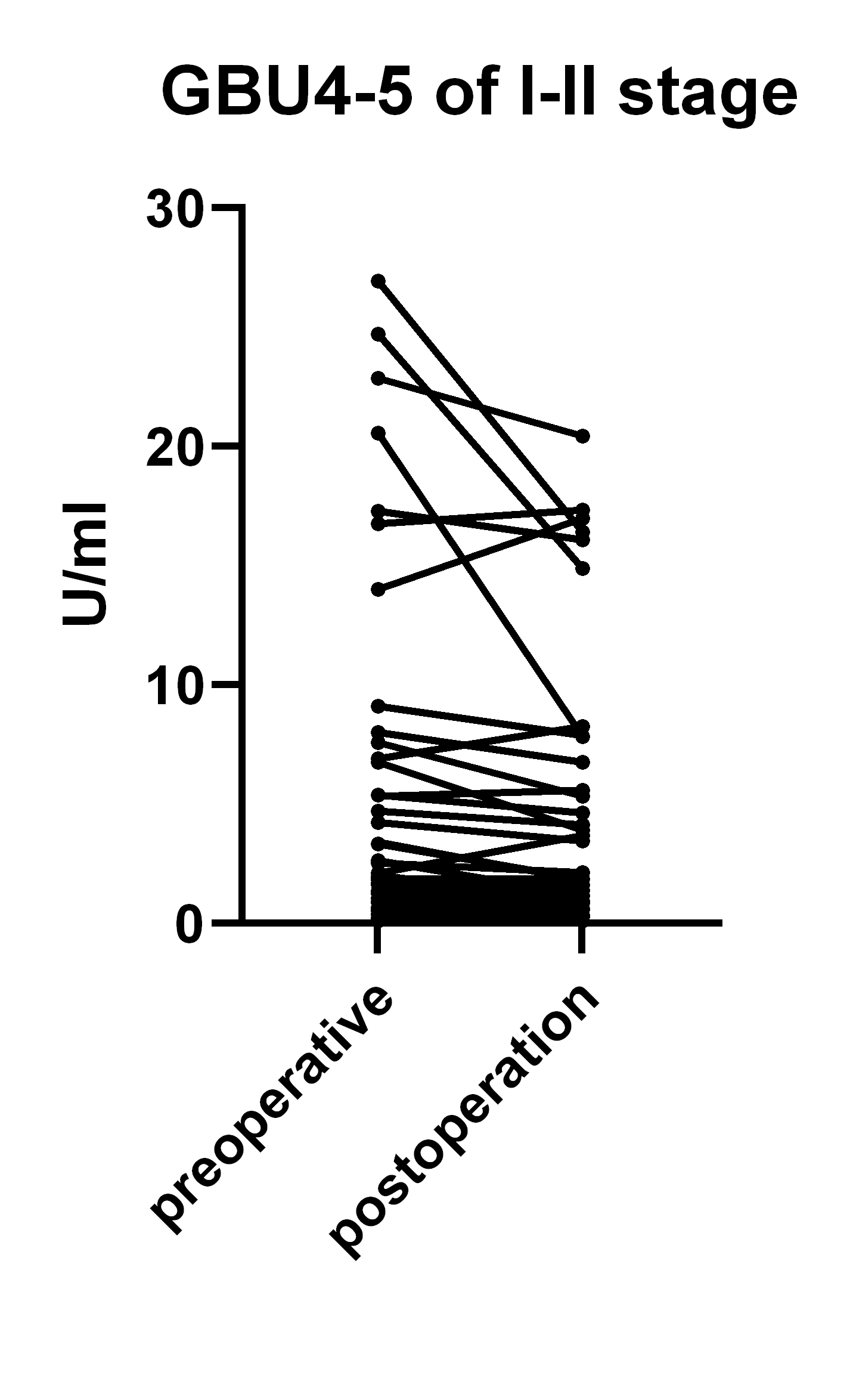

Supplement: Supplementary file 27 — Supplementary Material 27 [file 12890_2024_3060_MOESM27_ESM.png]

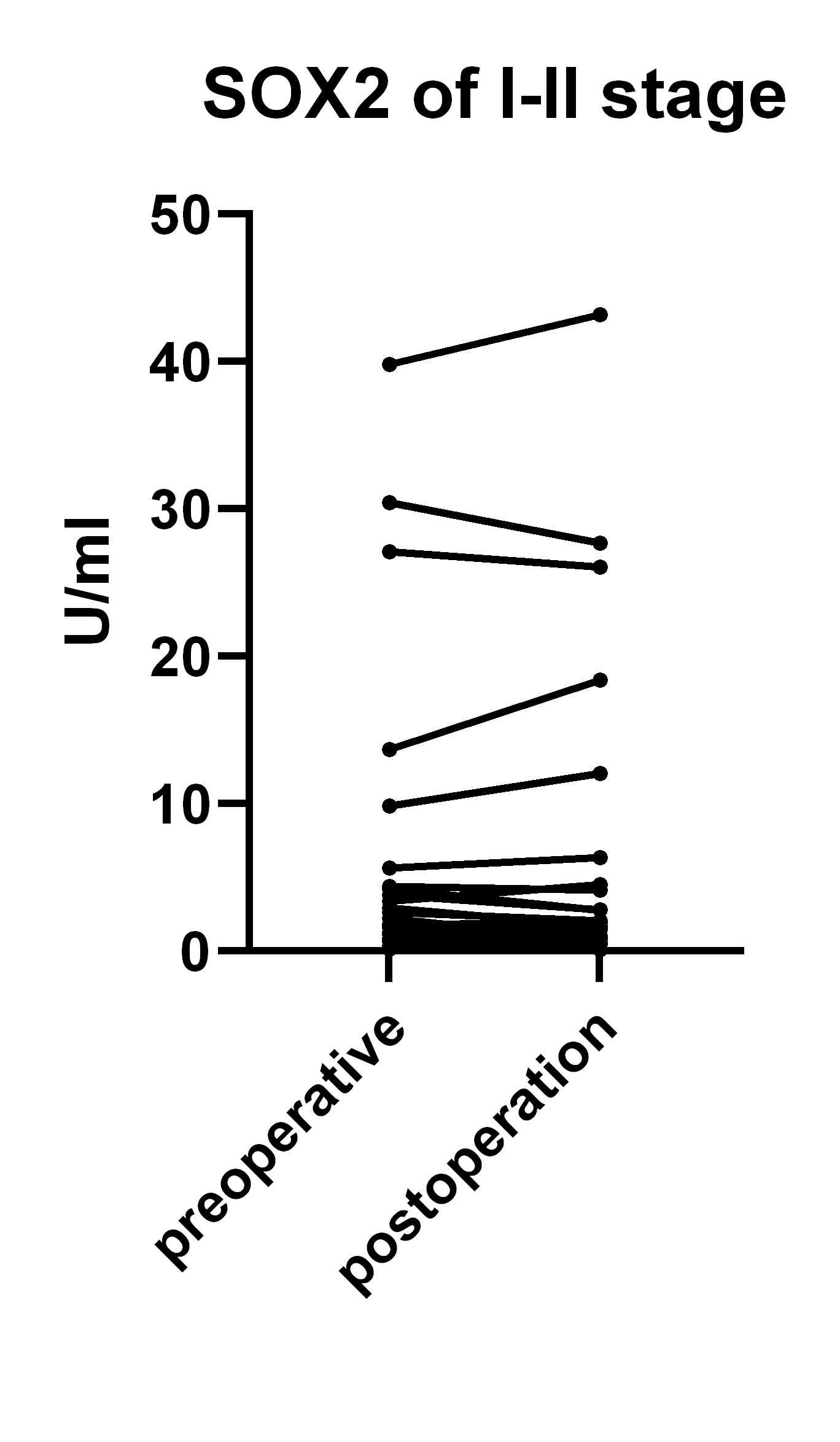

Supplement: Supplementary file 28 — Supplementary Material 28 [file 12890_2024_3060_MOESM28_ESM.png]

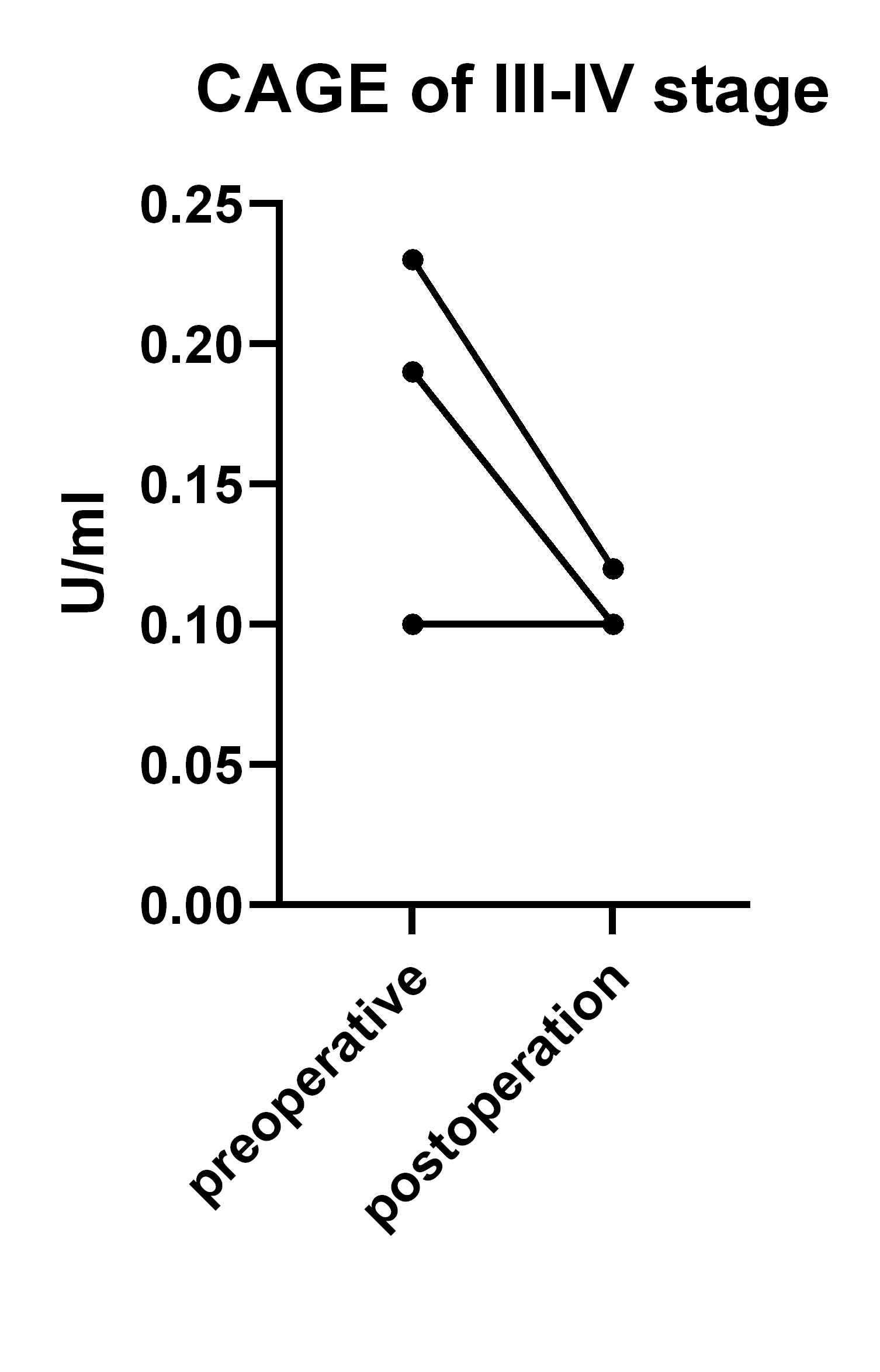

Supplement: Supplementary file 29 — Supplementary Material 29 [file 12890_2024_3060_MOESM29_ESM.png]

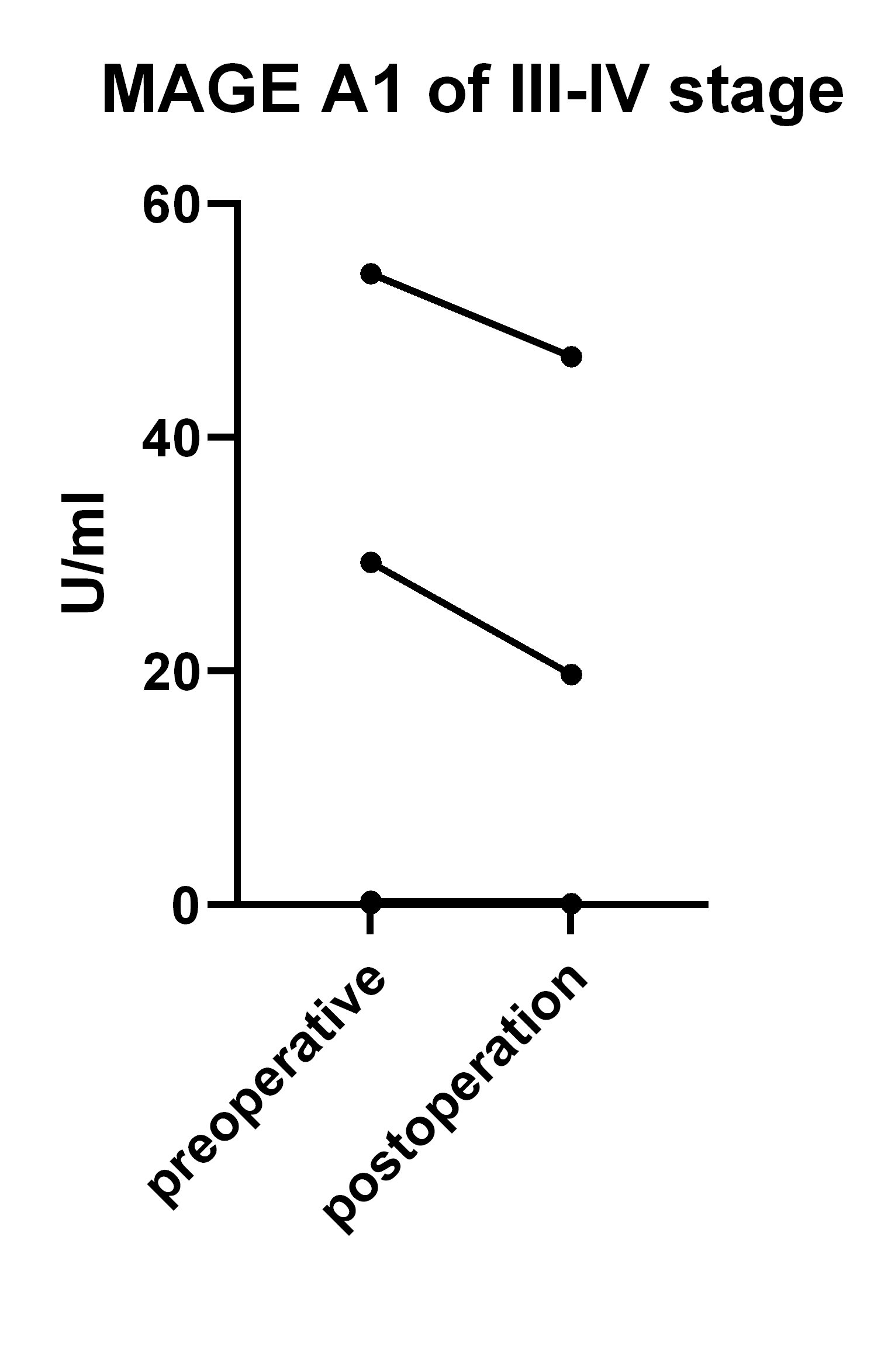

Supplement: Supplementary file 30 — Supplementary Material 30 [file 12890_2024_3060_MOESM30_ESM.png]

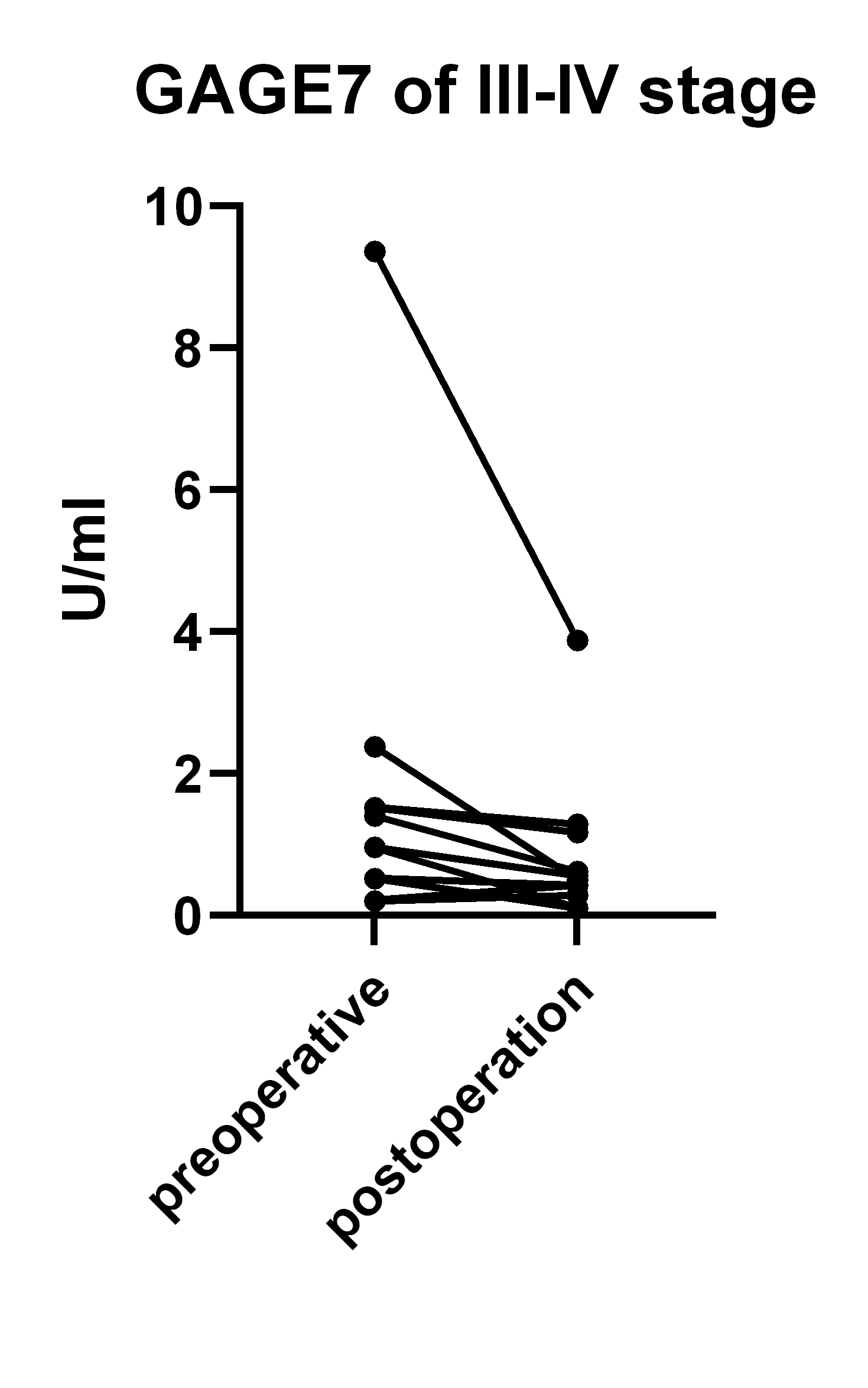

Supplement: Supplementary file 31 — Supplementary Material 31 [file 12890_2024_3060_MOESM31_ESM.png]

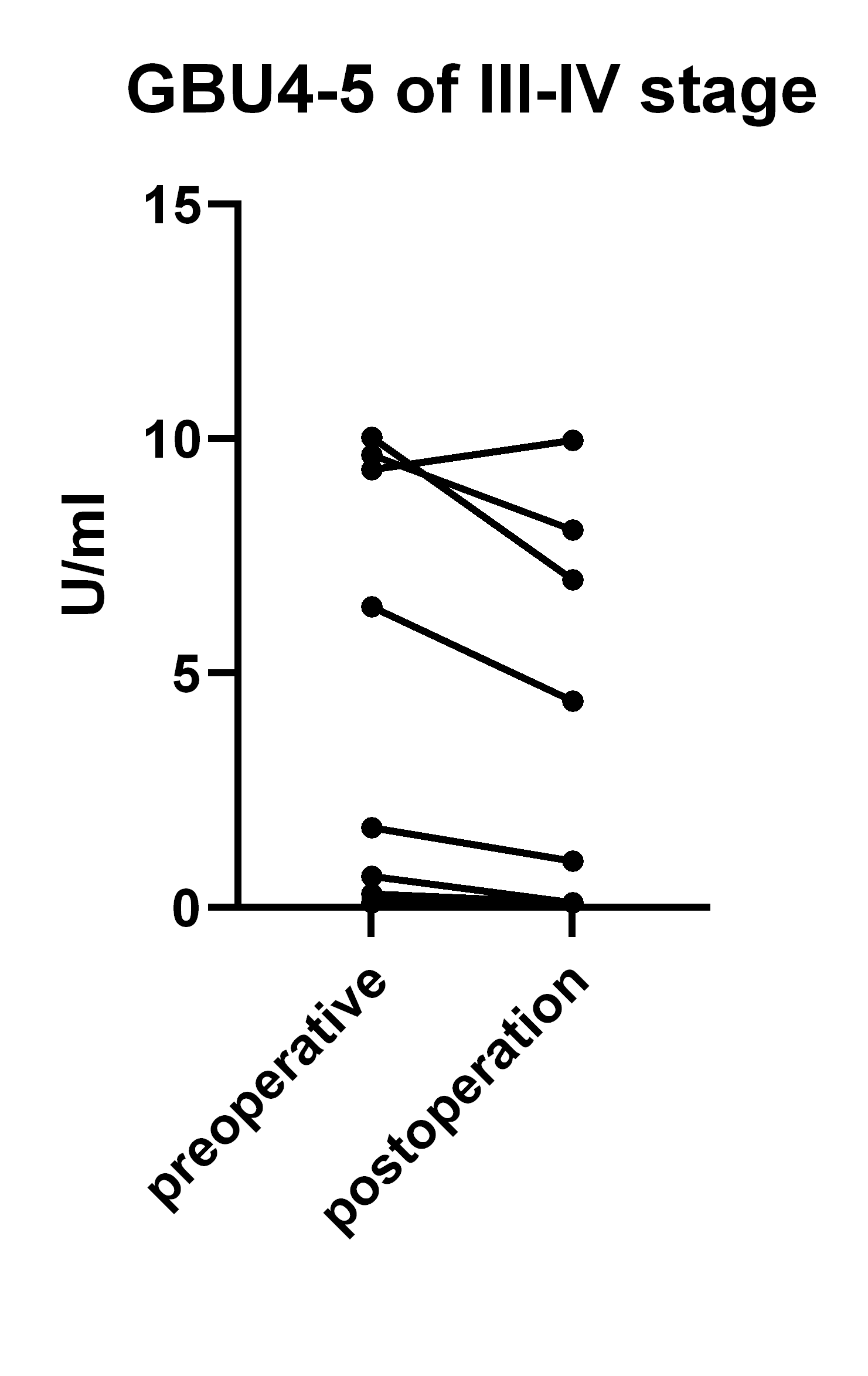

Supplement: Supplementary file 32 — Supplementary Material 32 [file 12890_2024_3060_MOESM32_ESM.png]

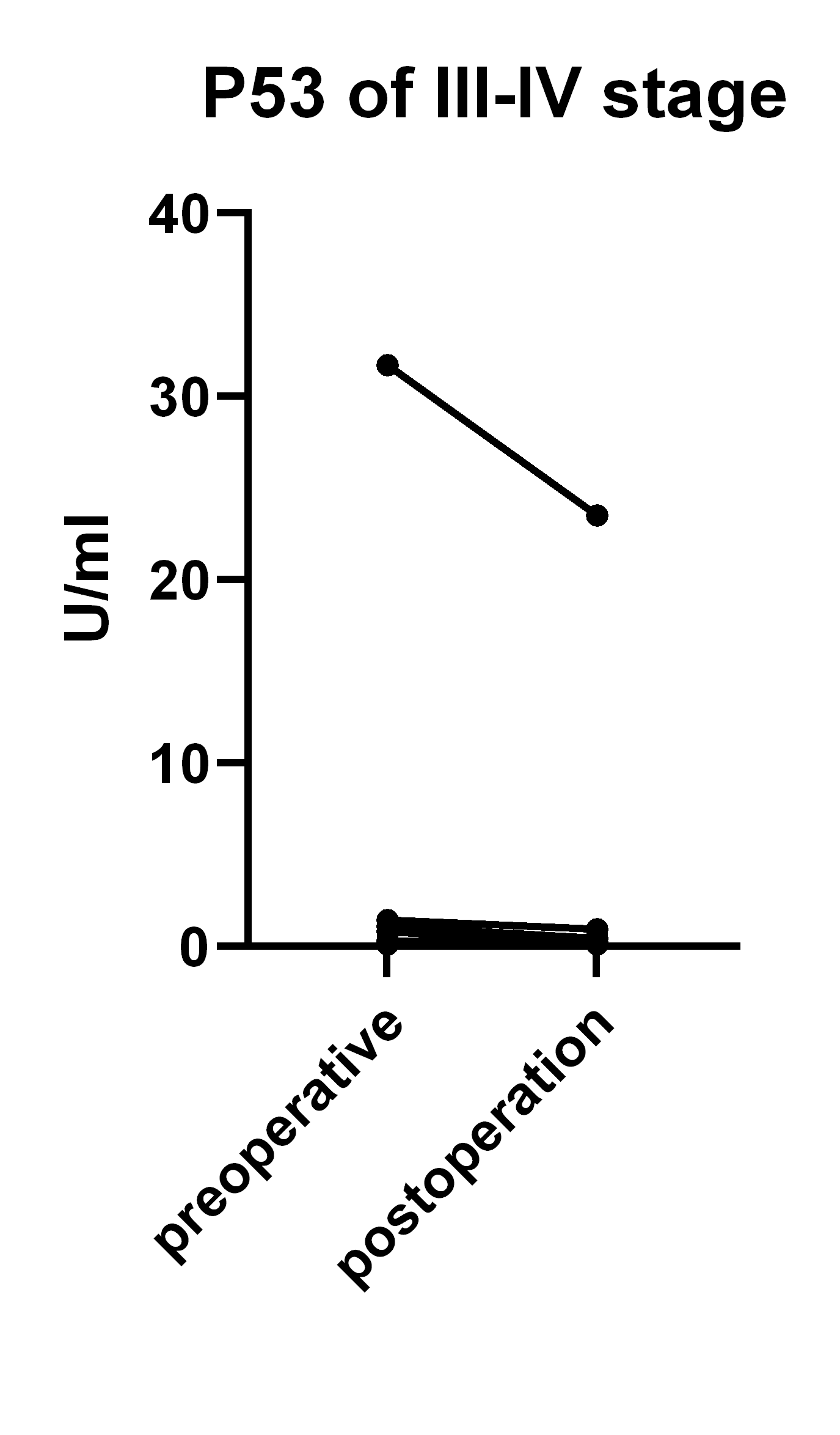

Supplement: Supplementary file 33 — Supplementary Material 33 [file 12890_2024_3060_MOESM33_ESM.png]

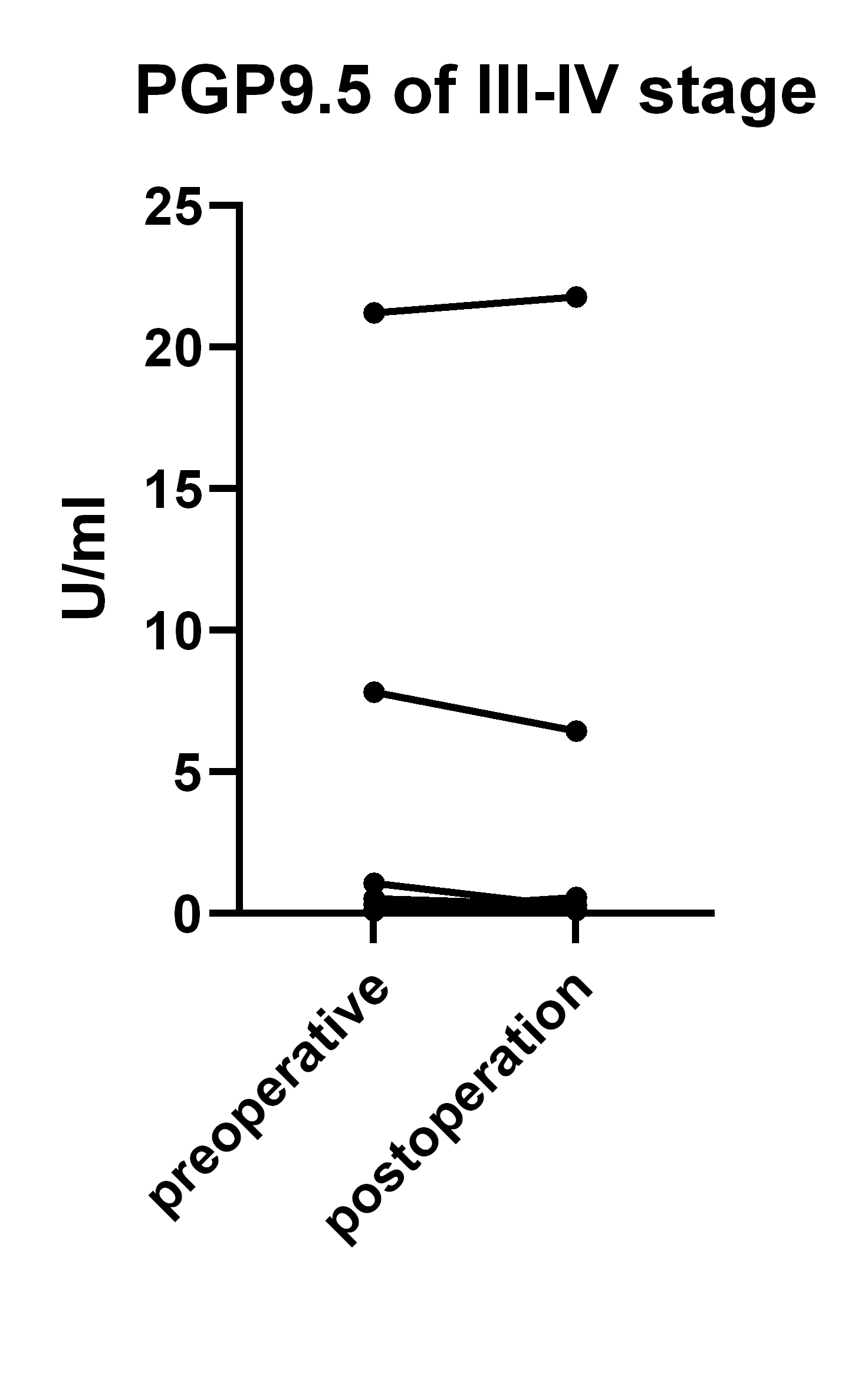

Supplement: Supplementary file 34 — Supplementary Material 34 [file 12890_2024_3060_MOESM34_ESM.png]

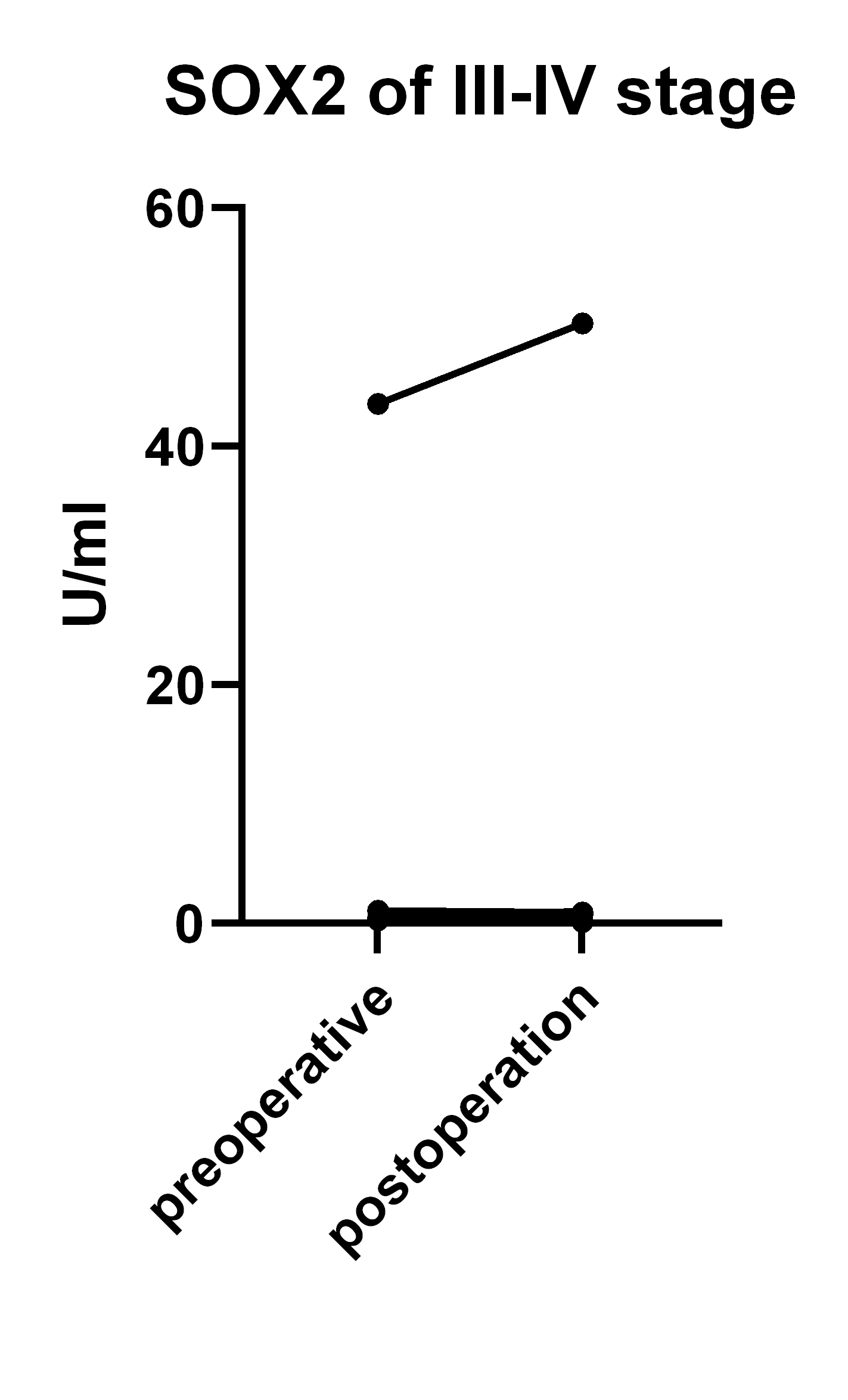

Supplement: Supplementary file 35 — Supplementary Material 35 [file 12890_2024_3060_MOESM35_ESM.png]

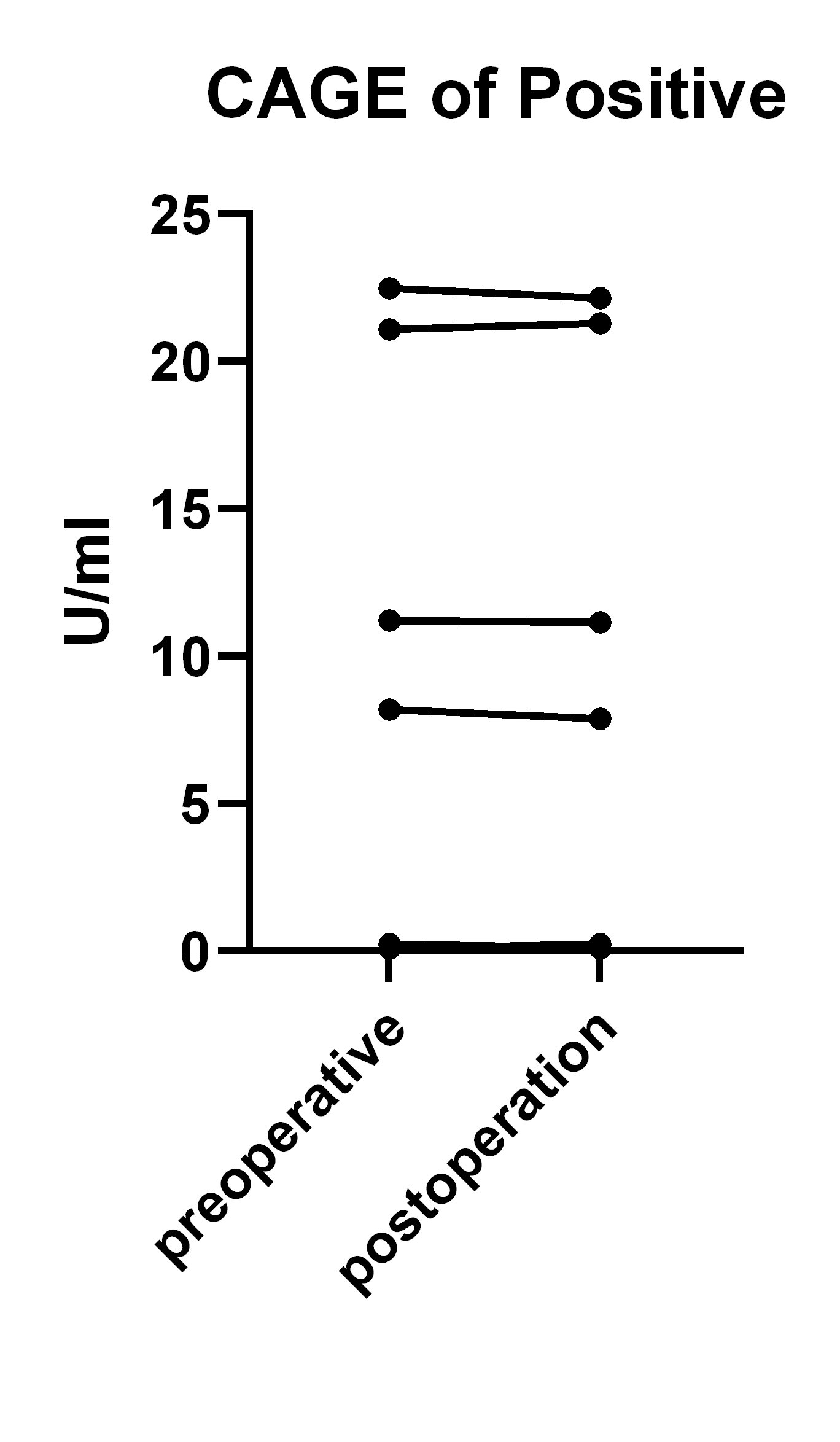

Supplement: Supplementary file 36 — Supplementary Material 36 [file 12890_2024_3060_MOESM36_ESM.png]

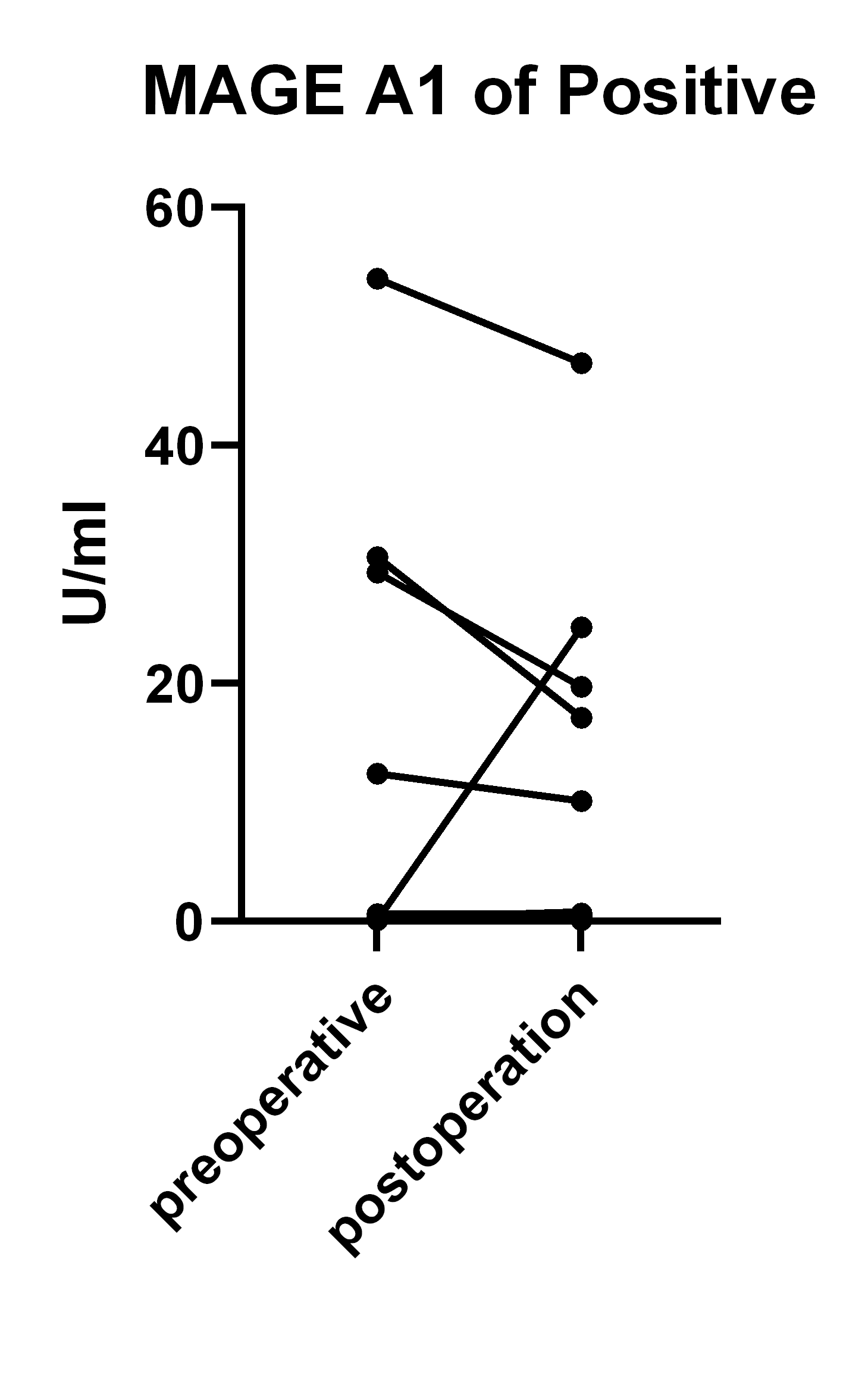

Supplement: Supplementary file 37 — Supplementary Material 37 [file 12890_2024_3060_MOESM37_ESM.png]

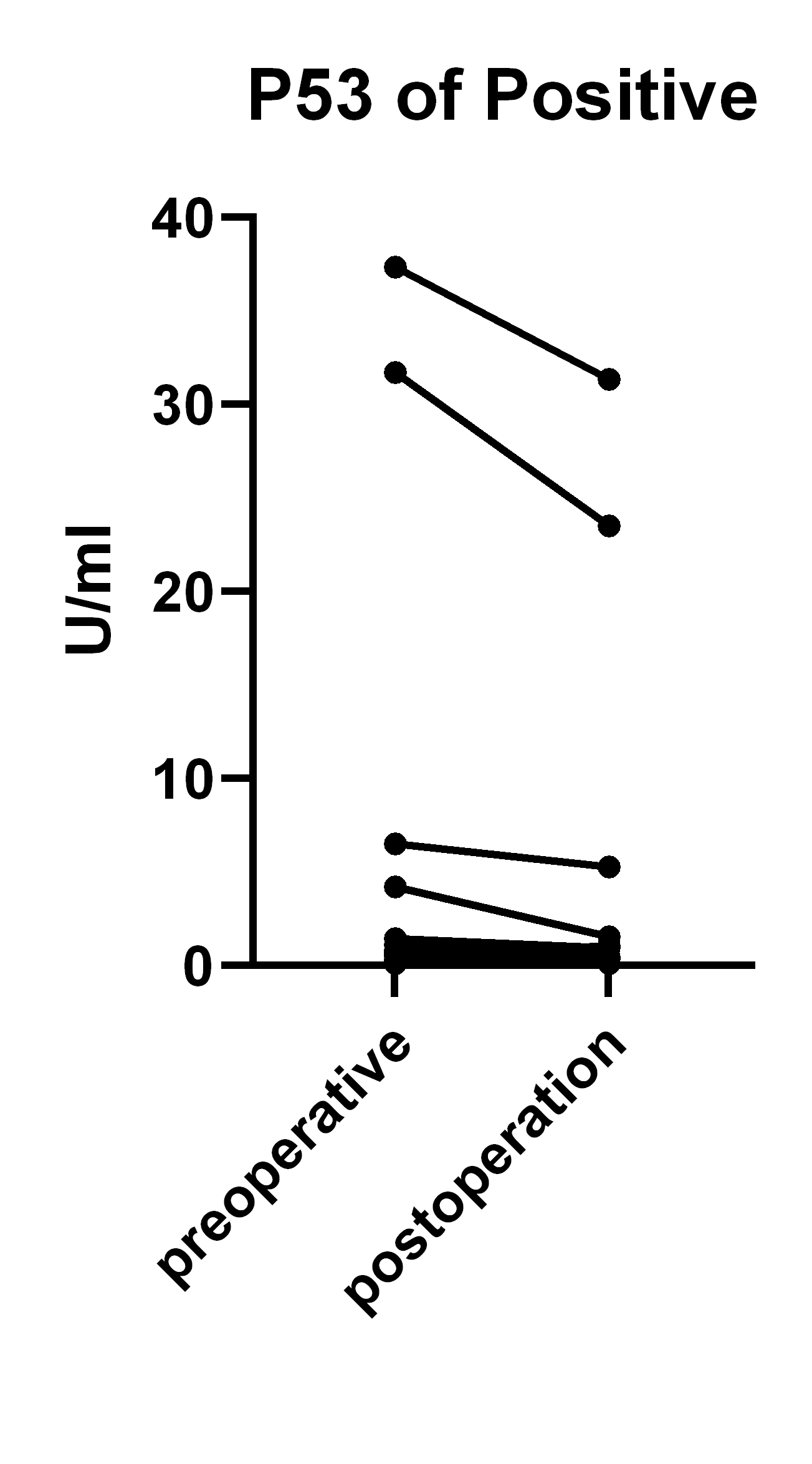

Supplement: Supplementary file 38 — Supplementary Material 38 [file 12890_2024_3060_MOESM38_ESM.png]

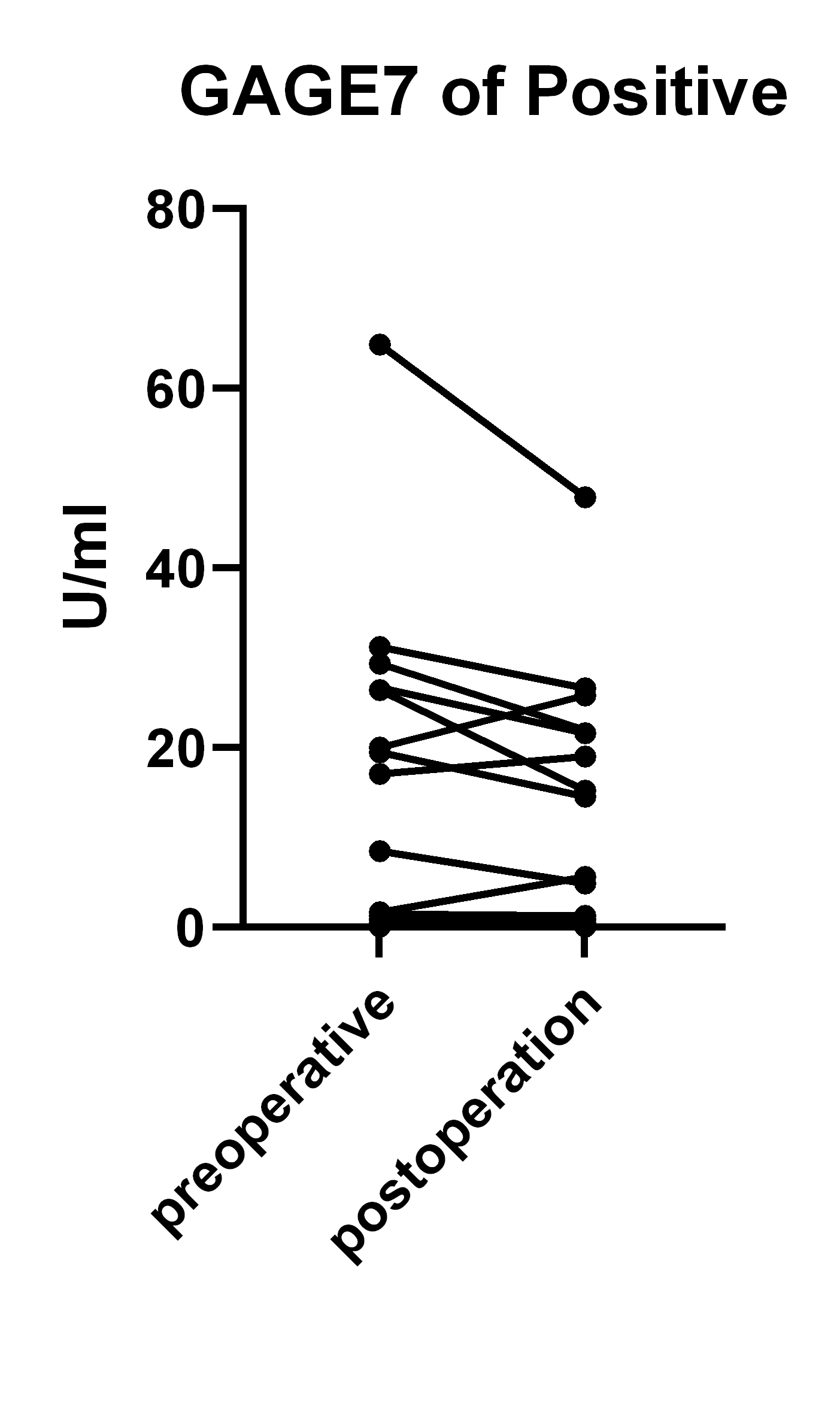

Supplement: Supplementary file 39 — Supplementary Material 39 [file 12890_2024_3060_MOESM39_ESM.png]

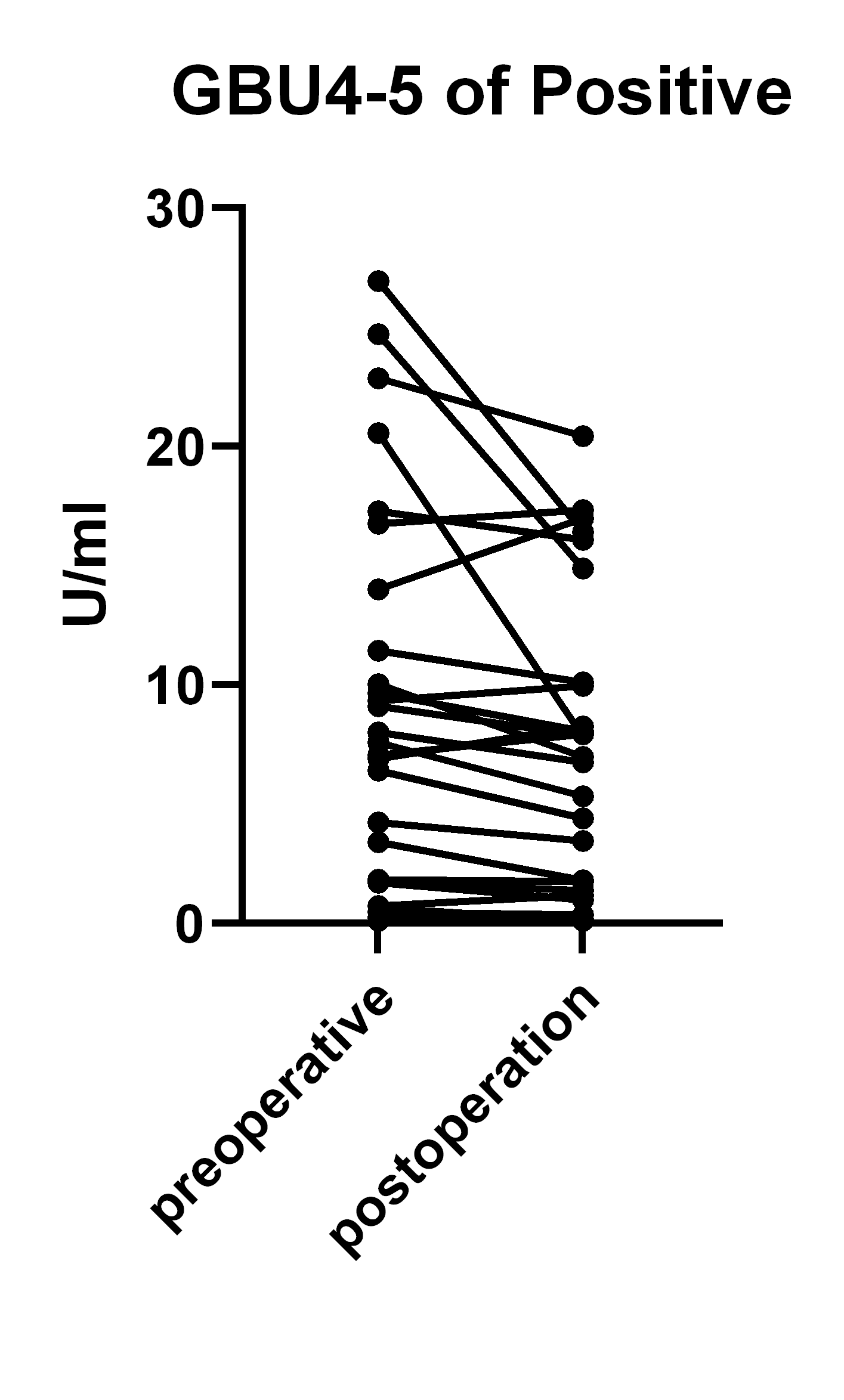

Supplement: Supplementary file 40 — Supplementary Material 40 [file 12890_2024_3060_MOESM40_ESM.png]

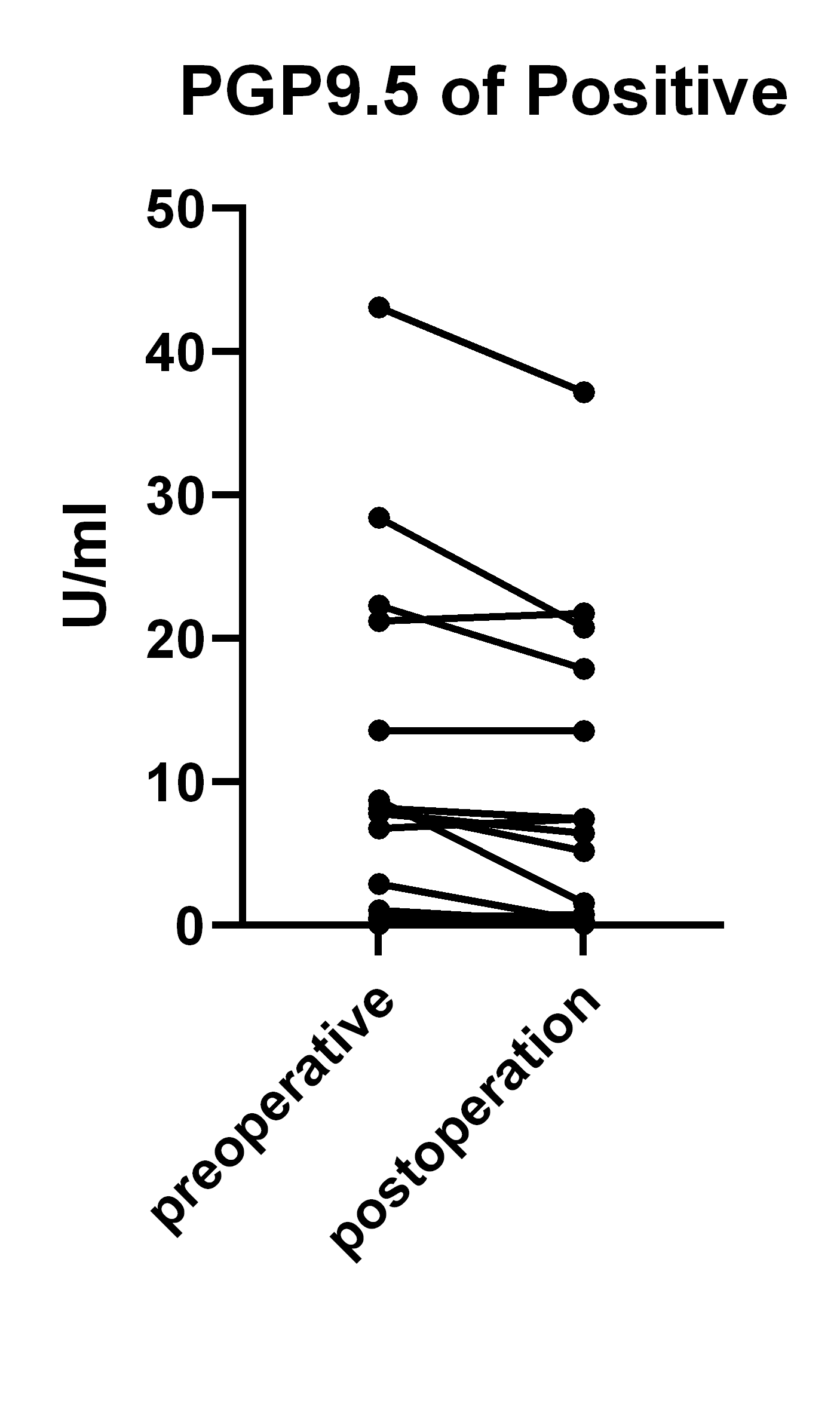

Supplement: Supplementary file 41 — Supplementary Material 41 [file 12890_2024_3060_MOESM41_ESM.png]

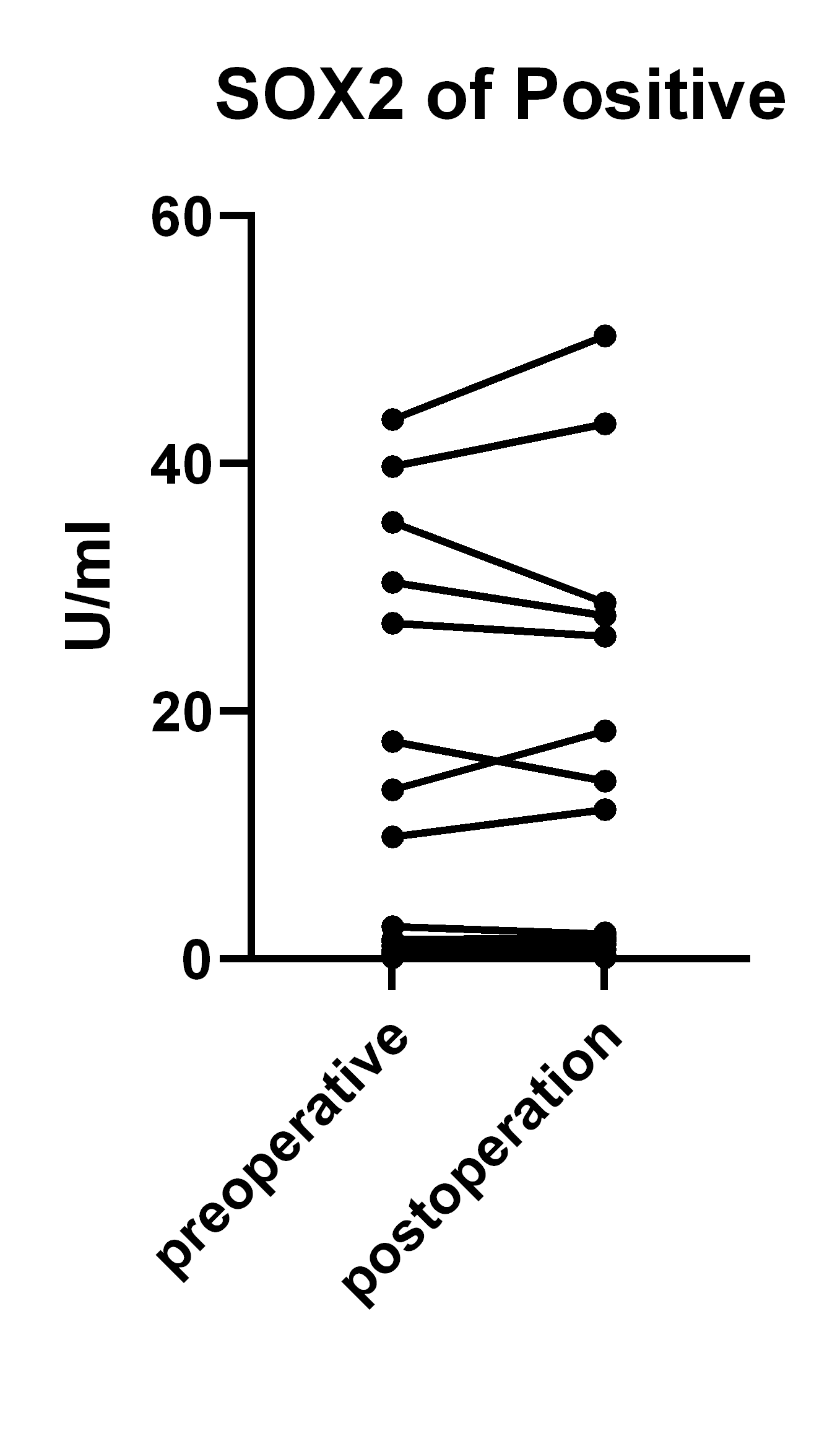

Supplement: Supplementary file 42 — Supplementary Material 42 [file 12890_2024_3060_MOESM42_ESM.png]

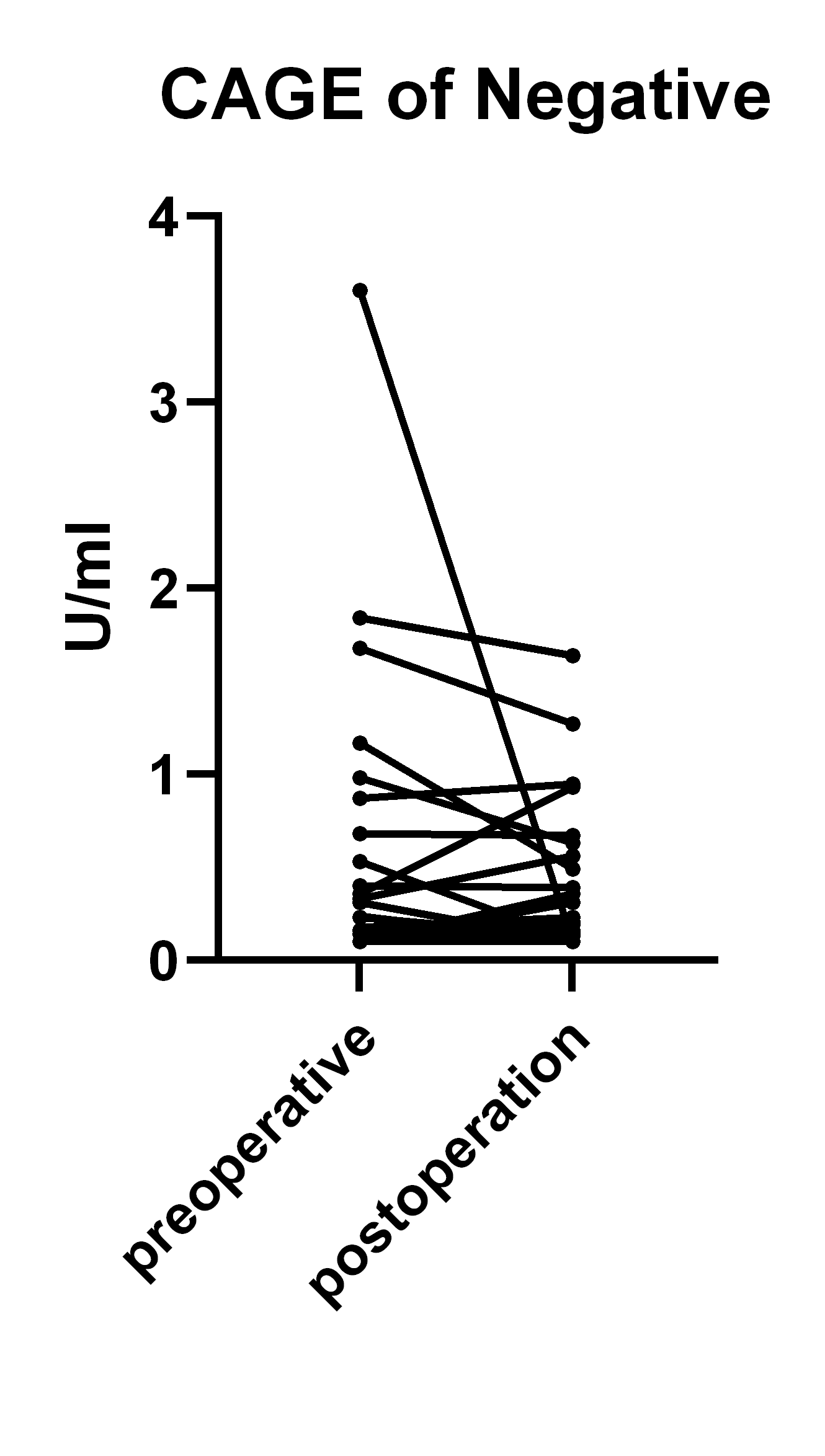

Supplement: Supplementary file 43 — Supplementary Material 43 [file 12890_2024_3060_MOESM43_ESM.png]

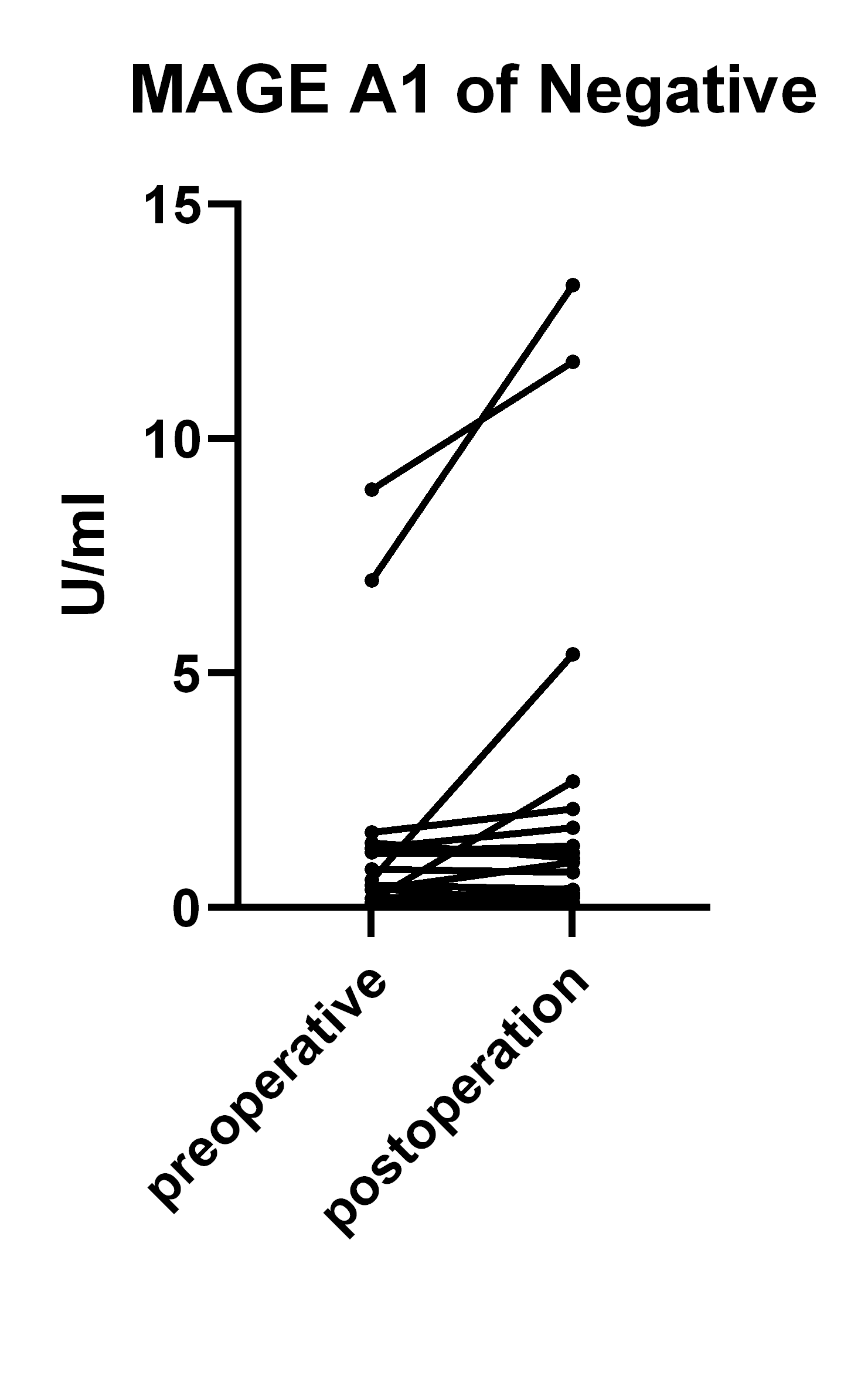

Supplement: Supplementary file 44 — Supplementary Material 44 [file 12890_2024_3060_MOESM44_ESM.png]

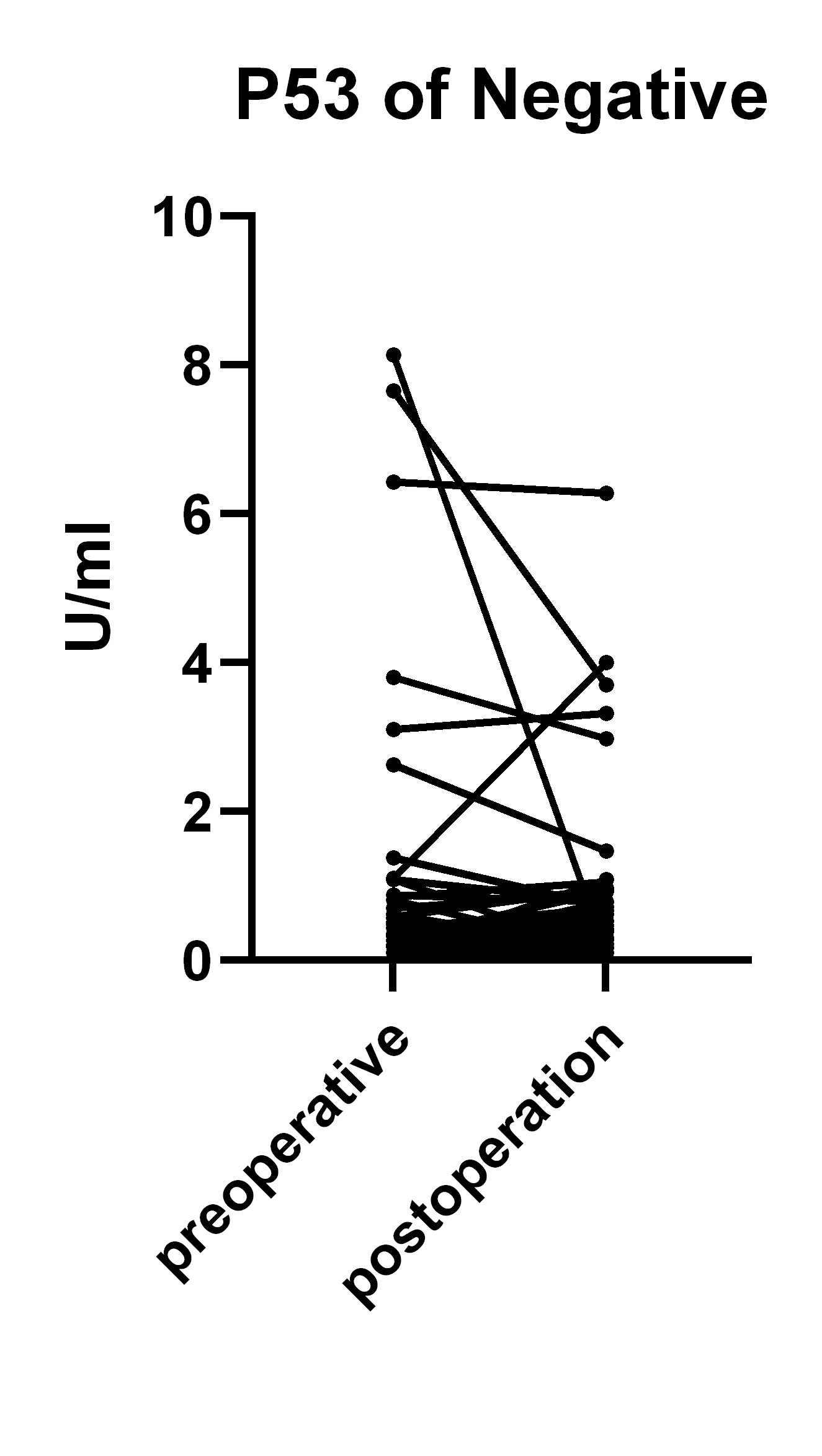

Supplement: Supplementary file 45 — Supplementary Material 45 [file 12890_2024_3060_MOESM45_ESM.png]

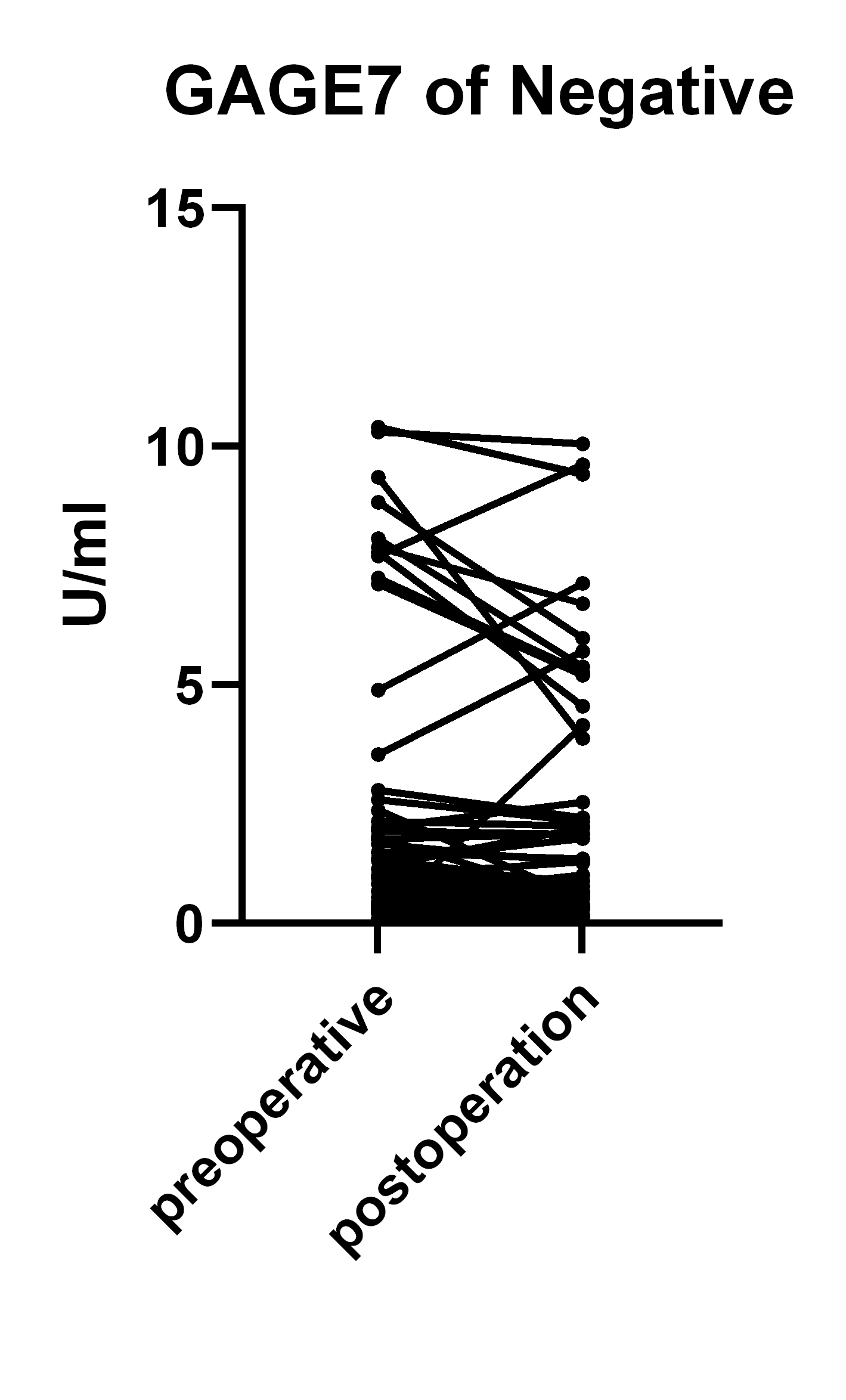

Supplement: Supplementary file 46 — Supplementary Material 46 [file 12890_2024_3060_MOESM46_ESM.png]

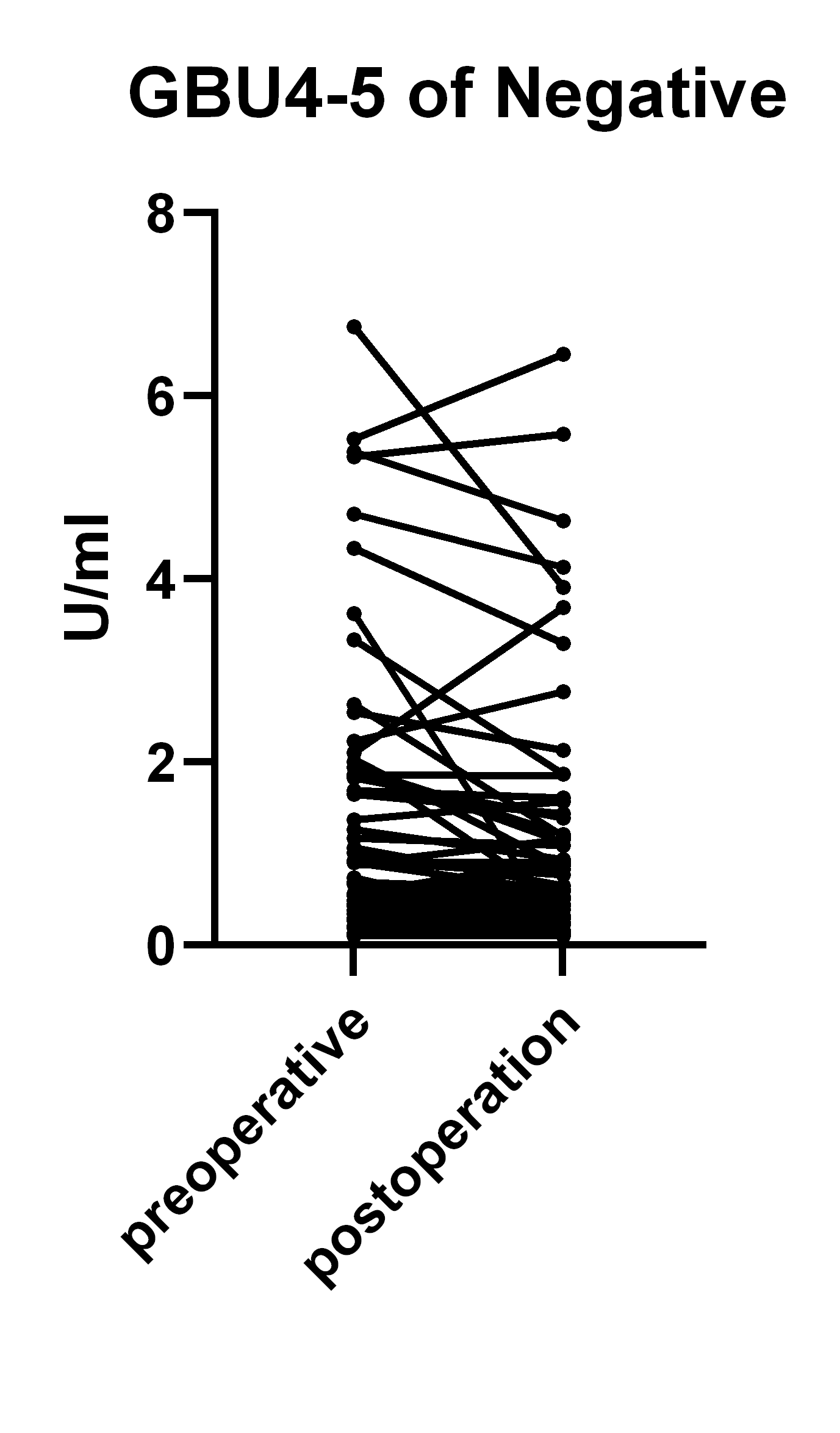

Supplement: Supplementary file 47 — Supplementary Material 47 [file 12890_2024_3060_MOESM47_ESM.png]

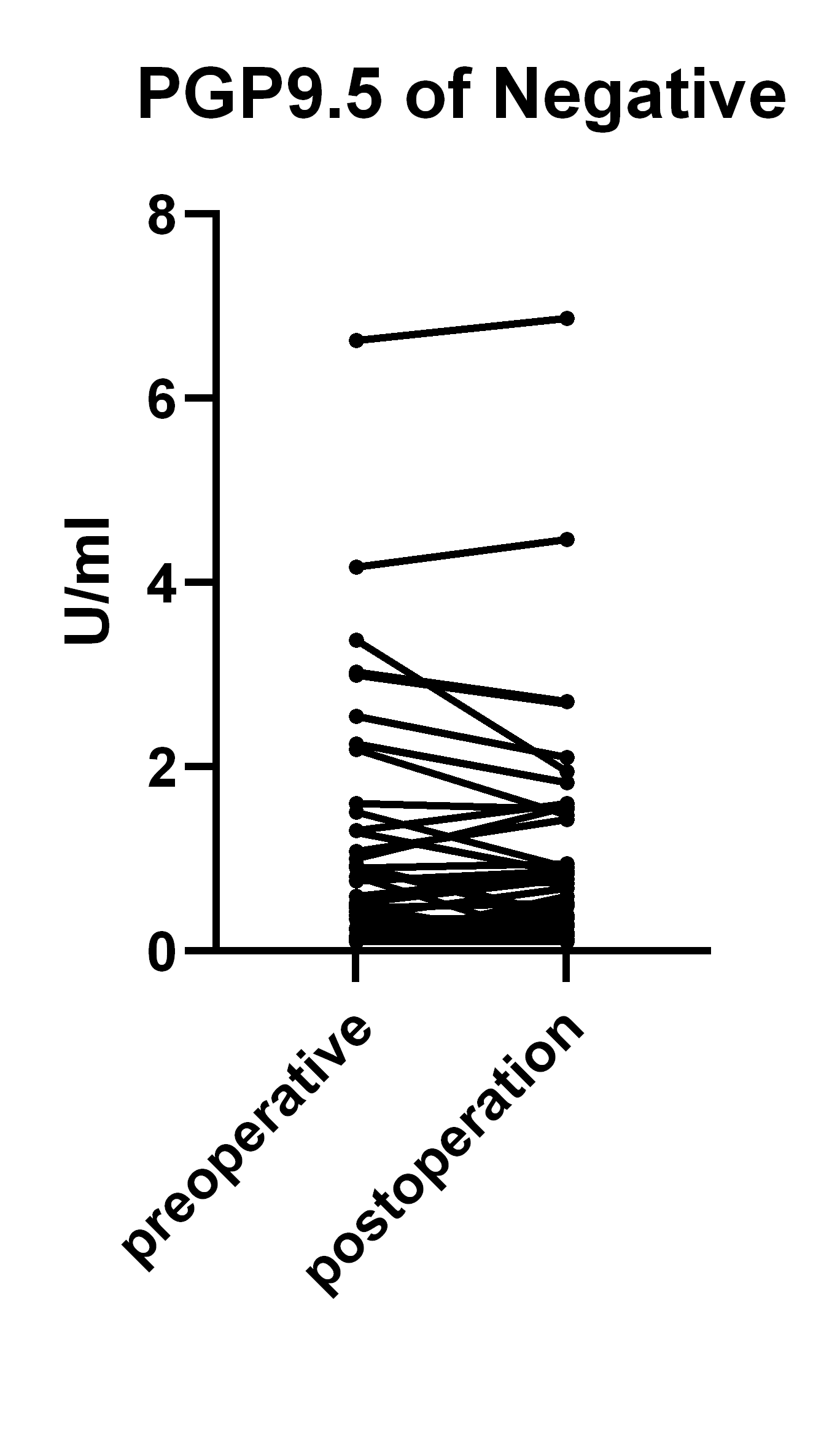

Supplement: Supplementary file 48 — Supplementary Material 48 [file 12890_2024_3060_MOESM48_ESM.png]

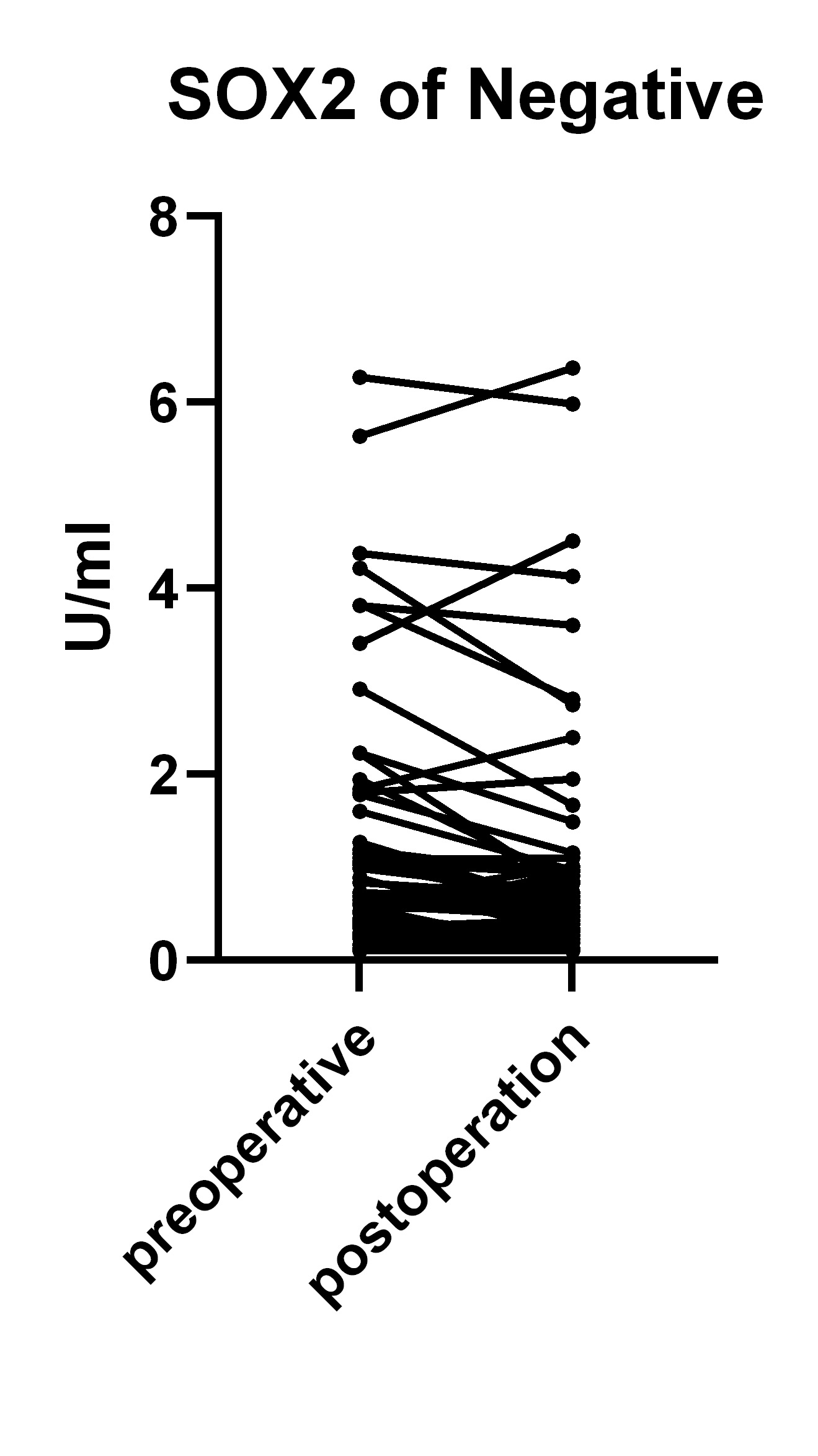

Supplement: Supplementary file 49 — Supplementary Material 49 [file 12890_2024_3060_MOESM49_ESM.png]
